# Modelling Red–Crowned Parrot (Psittaciformes: *Amazona viridigenalis* [Cassin, 1853]) distributions in the Rio Grande Valley of Texas using elevation and vegetation indices and their derivatives

**DOI:** 10.1371/journal.pone.0294118

**Published:** 2023-12-06

**Authors:** Elise Varaela Voltura, James L. Tracy, J. Jill Heatley, Simon Kiacz, Donald J. Brightsmith, Anthony M. Filippi, Jesús G. Franco, Robert Coulson

**Affiliations:** 1 Department of Veterinary Pathobiology, Texas A&M University, College Station, Texas, United States of America; 2 Schubot Center for Avian Health, Texas A&M University, College Station, Texas, United States of America; 3 Department of Entomology, Texas A&M University, College Station, Texas, United States of America; 4 Department of Small Animal Clinical Sciences, Texas A&M University, College Station, Texas, United States of America; 5 Department of Ecology and Evolutionary Biology, Texas A&M University, College Station, Texas, United States of America; 6 Department of Geography, Texas A&M University, College Station, Texas, United States of America; 7 Rio Grande Joint Venture, American Bird Conservancy, McAllen, Texas, United States of America; University 20 Aout 1955 skikda, Algeria, ALGERIA

## Abstract

Texas Rio Grande Valley Red–crowned Parrots (Psittaciformes: *Amazona viridigenalis* [Cassin, 1853]) primarily occupy vegetated urban rather than natural areas. We investigated the utility of raw vegetation indices and their derivatives as well as elevation in modelling the Red–crowned parrot’s general use, nest site, and roost site habitat distributions. A feature selection algorithm was employed to create and select an ensemble of fine–scale, top–ranked MaxEnt models from optimally–sized, decorrelated subsets of four to seven of 199 potential variables. Variables were ranked *post hoc* by frequency of appearance and mean permutation importance in top–ranked models. Our ensemble models accurately predicted the three distributions of interest ($\bar{x}$ Area Under the Curve [AUC] = 0.904–0.969). Top–ranked variables for different habitat distribution models included: (a) general use–percent cover of preferred ranges of entropy texture of Normalized Difference Vegetation Index (NDVI) values, entropy and contrast textures of NDVI, and elevation; (b) nest site–entropy textures of NDVI and Green–Blue NDVI, and percent cover of preferred range of entropy texture of NDVI values; (c) roost site–percent cover of preferred ranges of entropy texture of NDVI values, contrast texture of NDVI, and entropy texture of Green–Red Normalized Difference Index. Texas Rio Grande Valley Red–crowned Parrot presence was associated with urban areas with high heterogeneity and randomness in the distribution of vegetation and/or its characteristics (*e*.*g*., arrangement, type, structure). Maintaining existing preferred vegetation types and incorporating them into new developments should support the persistence of Red–crowned Parrots in southern Texas.

## Introduction

There is growing interest in how distributions of species of conservation concern are affected by alarmingly rapid and intense global anthropogenic landscape modification [1–4], especially because it typically reduces their abundance as well as the size of their distributions [5–7]. However, some sensitive species of conservation concern have adapted relatively well to life in the city and/or suburbia [8]. Further study is needed to better understand the patterns and processes of suburban adaptation to develop strategies that support their persistence and make urban landscapes more attractive to a wider diversity of “desirable” species [9]. Interestingly, the populations of Endangered Red-crowned Parrots (*Amazona viridigenalis* [Cassin, 1853]) found in the southern United States of America (USA) are typically absent from natural areas but instead seem to prefer certain highly–modified urban areas [10–14] and are found in cities in least three states (*i*.*e*., California, Florida, Texas) [15]. Those found in the Rio Grande Valley of Texas are the focus of this study. There is debate over how the founders of the Texas Rio Grande Valley populations arrived and whether they are native to the USA [13, 16–19]; however, we accept the position of the United States Fish and Wildlife Service (USFWS) [12, 20] that Red-crowned Parrots are native to South Texas [18]. As such, we consider the current natural range of the Red-crowned Parrot to stretch between the south–central region of Veracruz in Mexico and just north of the Rio Grande River in Texas in the USA [12]. Factors including habitat degradation and/or loss, poaching of individuals (especially young birds) for sale in the illegal wildlife trade, and the culling of large numbers of individuals by farmers who perceive them as pests have synergistically resulted in its extirpation [14, 21–24] from over 75% of its historical range in Mexico [12, 14, 25, 26]. As such, those found in southern Texas are of particular interest to many people.

Like so many other parrot species (order Psittaciformes) around the world [27], the future of the Texas Rio Grande Valley Red–crowned Parrots is far from guaranteed. The United States’ track record of protecting its native parrot species (n = 5) is poor. While, the once abundant Carolina Parakeet (*Conuropsis carolinensis* [Linnaeus, 1758]) was declared extinct in 1939, and the Thick–billed Parrot (*Rhynchopsitta pachyrhyncha* [Swainson, 1827]) has been considered functionally extinct throughout its historical range within the USA since 1995 [28]. As such, Red-crowned Parrots of the Texas Rio Grande Valley are one of the few native Psittaciformes (order of parrots) that persist in their natural range within the USA in viable numbers today [13]. Since their populations are currently fairly stable and are composed of a reasonable number of individuals (*ca*. 700 as of 2018 [13]), Americans have a unique opportunity to choose a path that leads to a different outcome for one of the last of its native parrot species without the need to pour massive amounts of capital into conservation efforts to do so. The purpose of this study is to take an important step forward on this path by striving to improve our understanding of the unique habitat requirements of the Texas Rio Grande Valley Red-crowned Parrots [29].

As with most land-dwelling birds, vegetation is a key factor to parrots’ survival wherever they are found [30]. Large, mature trees and certain shrubs are particularly valuable to parrots because they provide sources of food, places to nest (*i*.*e*., hollow tree cavities) and roost as well as refuge from threats such as predators and inclement weather [31]. As urban-dwelling parrots, we suspect that Texas Rio Grande Valley Red-crowned Parrot presence is likely particularly closely related to not only the presence of the necessary types of vegetation but patterns in the manner in which it is distributed [32, 33]. While a similar suggestion has been made for the urban–dwelling Red–crowned Parrots of southern California, it has not been investigated empirically for either sets of populations [10, 11, 34]. Our general aim is to evaluate this hypothesis further using a correlative species distribution modelling approach.

Correlative species distribution models (SDMs) are often critical components of conservation planning and management efforts for a variety of sensitive species, including birds such as parrots [34–37] because they can help reveal how the presence (or absence) of a given species is related to the environmental conditions (and resources) necessary for its survival [38]. A wide array of variables has been employed in SDMs to elucidate what these conditions and resources might be from what is often a long list of possibilities. Common types of said variables include those that represent the effects of climate (*e*.*g*., temperature, precipitation, humidity, etc.), topography or bathymetry (*e*.*g*., elevation or depth, slope, aspect, etc.), biogeochemistry (*e*.*g*., soil, hydrology, irradiation, etc.), and land use/land cover (*e*.*g*., class, disturbance or change, patch characteristics, distance to/from critical type, etc.) on SDMs [39]. We focus on vegetation–based variables in our SDMs based on the potential aforementioned connection and because vegetation presence (or absence) is often strongly associated with environmental conditions (*e*.*g*., climate, biogeochemistry, topography) [40–43]. Furthermore, vegetation is often one of the primary characteristics of landscapes used to define different land-use land-cover (LULC) classes [44, 45], and LULC type variables are increasingly employed in SDMs. We do not employ LULC variables in our SDMs for two reasons. First, many of the pre-processing steps required to create LULC maps (*e*.*g*., geometric, radiometric, solar, and atmospheric corrections and orthometric rectification applied to satellite imagery followed by supervised or unsupervised classification) [46–49] add their own type and level of variability and error that impact the qualitative and quantitative value of the final product(s) [50]. This can impact their suitability for use in SDM-type applications [51]. Second, LULC maps are invariably products of how humans see the world, and evaluating predictor variables less affected by this bias was of great interest to us [52].

The primary goals of this study are to examine whether vegetation influences Red–crowned Parrot habitat suitability in the Texas Rio Grande Valley using predictive modelling tools and to create maps of the distribution of suitable habitat for the species in the region. Our first specific objective is to evaluate the utility of vegetation–based predictive variables (*i*.*e*., topography, raw vegetation indices and their derivatives) in three high–quality, fine–scale MaxEnt habitat distribution models for Texas Rio Grande Valley Red–crowned Parrots: (a) general use, (b) nest sites, and (c) communal roost sites [13]. These three habitat distributions are modelled separately because the Red–crowned Parrots’ use of the landscape changes seasonally [53]. For example, Red–crowned Parrots roost communally throughout the year; however, the size and social structure of the communal roosts varies seasonally. During fall and winter, roosts are large and are comprised of members of all ages but as spring approaches, the size of roosts decreases (but number of roosts increases) as separate groups begin to disperse into smaller flocks; these smaller roosts subsequently shrink further as individual pairs disperse to nest [54]. Nest sites are only occupied during the breeding season, which occurs from March to July (personal observation, Simon Kiacz). Our second specific objective is to create and study a series of projections of each of the three habitat distributions.

To achieve our first objective, we will develop a series of variables derived from aerial imagery that we will use to study the presence of and patterns in vegetation in the region of interest in a practical, low–cost manner at relatively high resolutions [55]. We will explore vegetation indices as predictor features in our SDMs as they are among the most common tools used to detect and study vegetation using spectral data extracted from aerial imagery [56–59]. Vegetation indices have been used in an increasing number of SDMs for various taxa in recent years, including birds [60–65], however, this is one of the first involving parrots. In addition to the simple presence or absence of vegetation (or certain characteristics of vegetation), the distribution of birds can be influenced by patterns in its distribution and/or those of its defining characteristics (*e*.*g*., arrangement, composition, diversity, structure, etc.) [66–68]. As such, our list of environmental predictor features will include certain derivatives of raw vegetation indices which contain information about said patterns as seen from the perspective of the parrots themselves (*i*.*e*., percent cover of preferred ranges of vegetation index values, texture of raw vegetation indices, and percent cover of preferred ranges of vegetation index textures). We will use the cross–validated random subset feature selection algorithm (RSFSA–CV) [69] to identify top–performing MaxEnt models [70–72] and rank the performance of variables. Finally, we will use a set of 12 randomly selected top-performing models to create frequency of consensus projections for each suitable habitat distribution of interest and study their similarities and differences.

## Materials and methods

### Ethics statement

All experimental procedures were evaluated and approved by the Institutional Animal Use Care and Committee (IACUC) of Texas A&M University (TAMU) as well as by the ethics committee of the Texas Wildlife Research Program for the Texas Parks and Wildlife Department (TPWD). This study was conducted under IACUC Animal Use Protocol number 2018–0089 and TPWD Scientific Research Permit number 0418–138. Researchers made every effort to observe the parrots that were being studied for this project in a manner that caused as little disturbance to them as possible. All observations of the birds whose locations and activities were monitored for this study were obtained visually. This study was thus conducted without any need to have physical contact with the parrots of interest.

### Species of interest

**Fig 1:**
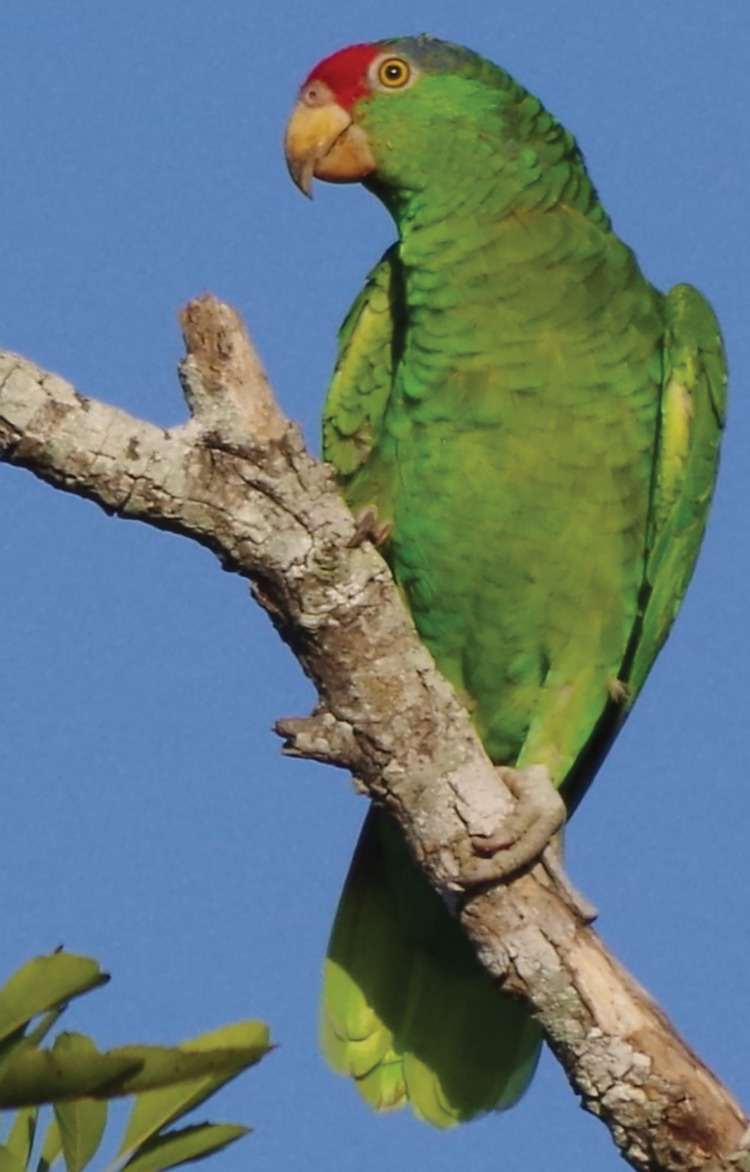
The species of interest–Red–crowned Parrot (*Amazona viridigenalis*). A Red–crowned Parrot perches on a tree branch in the Rio Grande Valley of Texas. Photo by Simon Kiacz *ca*. 2017.

The focal species is the Red–crowned Parrot (Fig 1). It is approximately 12 in long with a short, rounded tail. Their color is described as being mostly a dull yellowish–green that is somewhat “scaly”. They can be distinguished from other *Amazona* species by the combination of their distinct red crown and forehead, pale green cheeks, and flashy red wing patches [54].

### Study area

**Fig 2:**
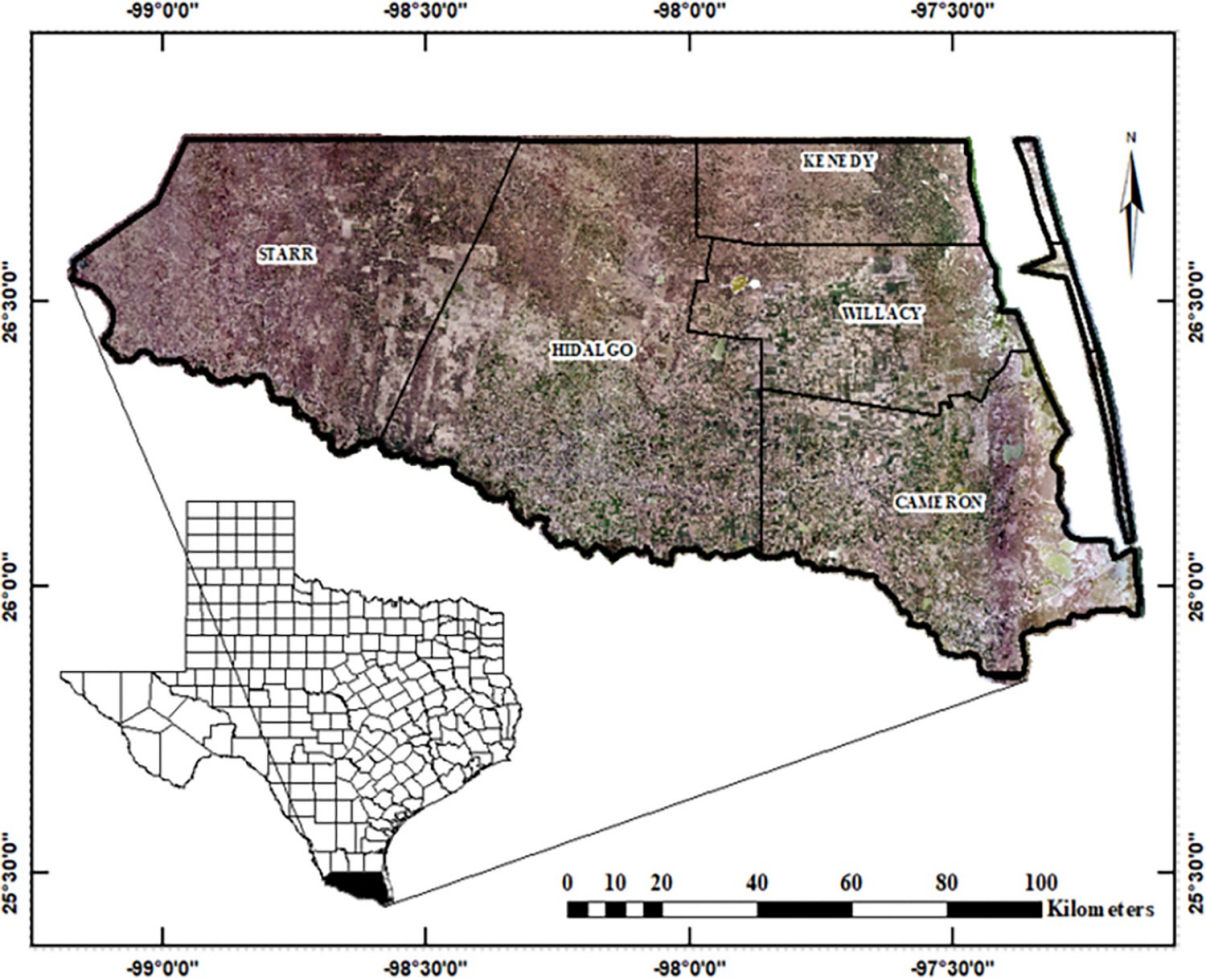
The study area–Rio Grande Valley of Texas. The Rio Grande Valley of Texas, USA includes Starr, Hidalgo, Cameron, and Willacy counties as well as the southern third of Kenedy County. The southern border of the study area is the Rio Grande River, which separates the USA from Mexico.

The Rio Grande Valley of Texas, the area of interest, is located at the southernmost tip of the state. It is typically defined as the region encompassed by Starr (1,383 km^2^), Hidalgo (4,100 km^2^), Willacy (1,530 km^2^) and Cameron (3,305 km^2^) counties and the southern third of Kenedy County (3,776 km^2^; Fig 2).

### Red–crowned Parrot presence points

**Fig 3:**
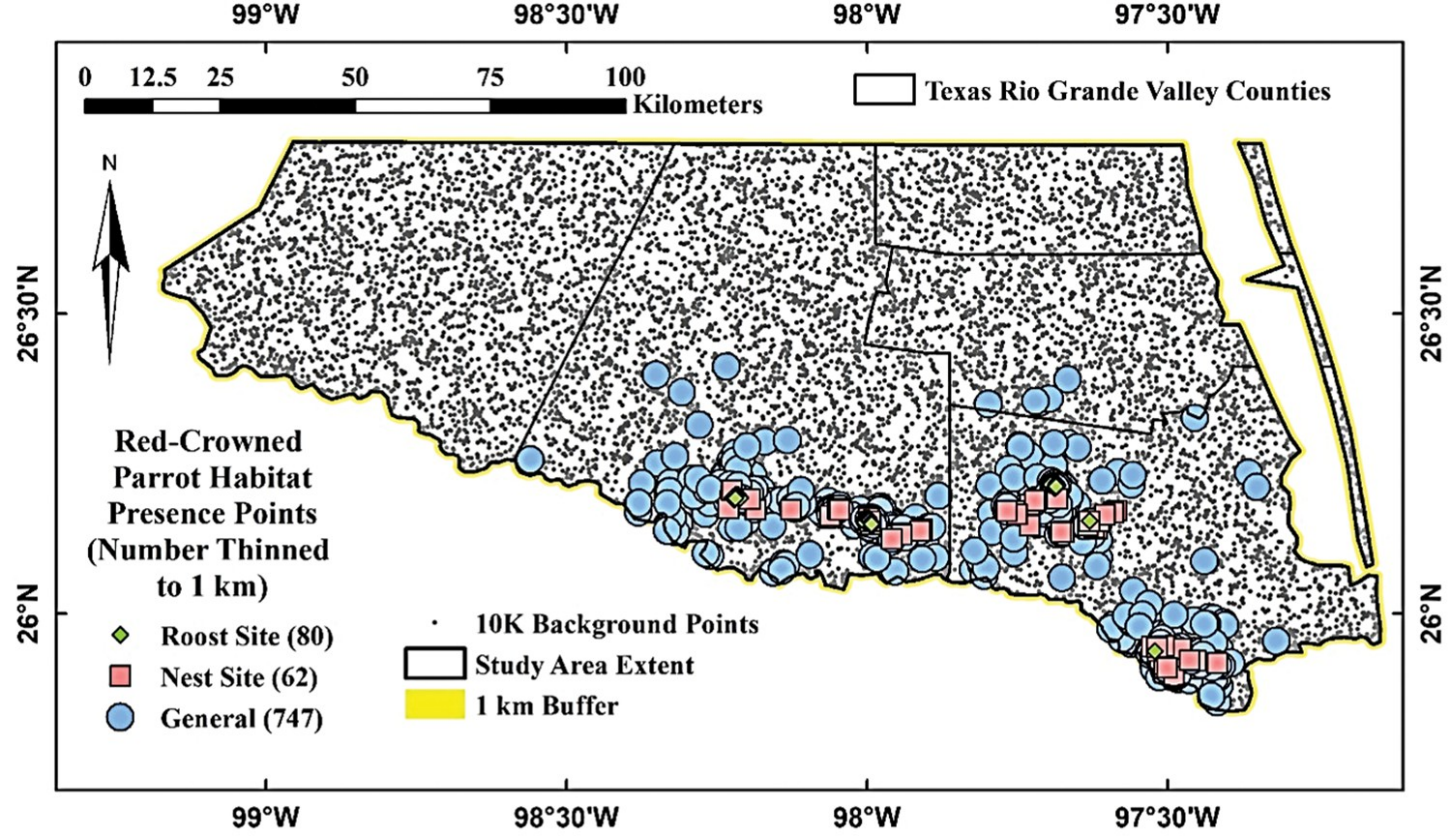
Texas Rio Grande Valley Red–crowned Parrot general use, nest site, and roost site presence points and background points used in habitat distribution models. Green diamonds indicate presence at roost sites. Pink squares indicate presence at nest sites. Blue circles indicate presence at uncategorized locations. Black dots indicate randomly generated background points.

Red–crowned Parrot presence points (n = 967) from the study area used for the general habitat distribution models (without regard to season or behavior) were obtained from eBird and iNaturalist prior to March 2021, as well as from the Tejano Parrot Project between 2014 and 2019 (Fig 3) [73–75]. Presence points used to develop the nest site (n = 61) and communal roost site (n = 79) habitat distribution models were acquired via surveys conducted by the Tejano Parrot Project between 2014 and 2019 (Fig 3) [74]. Only presence points with a positional accuracy of an arbitrarily selected 100 m or less were used for modelling (*i*.*e*., we had high confidence that the true location of any given point was located in a pixel included within the area of a circle of a diameter of 100 m or less where the point was the circle center). For eBird data, this meant we only utilized data collected using “Incidental”, “Stationary”, and “My Yard Counts” sampling protocols [76]. Eighty percent of the presence points representing sightings of Red-crowned Parrots within general use habitat (n = 747) were allocated to modelling and 20% (n = 220) were withheld for calculating new variables based upon parrot-preferred ranges of other variables (described below). These points were withheld from modelling to avoid confounding points used in variable calculations with points used in the MaxEnt models. All nest site and roost site presence data were allocated to modelling. Finally, presence points allocated for modelling were spatially filtered such that only a single random point within an area of a circle with a diameter of 100 m was used wherever a cluster of points occurred to reduce spatial autocorrelation (*e*.*g*., Boria et al. [77]) using ArcMap [78].

Presence data will not be made generally available to the public to protect the parrots from disturbance and poaching; however, the data can be requested via the eBird [79] and iNaturalist [80] websites or by contacting Dr. Donald J. Brightsmith, the director of the Tejano Parrot Project [74].

### Background and pseudoabsence points

We generated 10,000 background points, the MaxEnt default [81], separated from one another by a distance of at least 100 m to reduce spatial autocorrelation using ArcMap (Fig 3). Pseudoabsence was represented by a set of 10,000 points that were generated in the same fashion as background points with the exception that we filtered out all pseudoabsence points located within 200 m of any true presence point (n = 9,814).

### Environmental predictor features

We considered 199 environmental predictor features (variables), each of which falls into one of seven categories, in our SDM efforts: (1) *topography* (n = 1), (2) *raw vegetation index* (n = 8), (3) *multitemporal vegetation index difference* (n = 4), (4) *percent cover of preferred ranges of raw vegetation index values* (n = 24), (5) *raw vegetation index textures* (n = 48), (6) *texture of binary preferred/non–preferred ranges of raw vegetation index values* (n = 48), and (7) *percent cover of preferred ranges of vegetation index texture values* (n = 66; total N = 199; S1 Table). Detailed descriptions of the features in each of these categories are provided below.

Each predictor feature was a 9.5 m resolution raster projected to the 1983 North American Datum (NAD83) that covered the study area plus a one km buffer, which was added to eliminate edge effects introduced by the focal neighborhood calculations used to derive certain feature types. Feature rasters were converted from floating-point to integer format with a maximum of seven single digits to reduce raster size and computational complexity. Additionally, we applied a few additional non–linear transformations to certain feature rasters to magnify differences between pixel values (S1 Table). All feature rasters were clipped to match a study extent raster to ensure they were perfectly aligned with one another, a vital step in preprocessing variables used in MaxEnt modelling, and then converted to Tag Image File Format (TIFF) files using ArcMap for further processing in R Studio, an integrated develop environment software [82].

#### Topography

Since topography can have a strong influence on the distribution of vegetation, we elected to assess the utility of a single representative feature, a simple digital elevation model in our SDMs [83–85]. We downloaded each of the USGS National Elevation Dataset 10 m resolution rasters for the study area [86] and mosaiced them (*e*.*g*., pieced together like a puzzle) using ArcMap. Finally, we resampled the resulting raster to 9.5 m to create the *elevation* feature (S1 Fig).

#### Raw vegetation indices

Vegetation indices can be used to qualitatively and quantitatively study a variety of characteristics of vegetation (*e*.*g*., type, presence of vegetative cover versus non–cover, vigor, growth dynamics, etc.) using information derived from spectral reflectance data [56, 59]. Hundreds of different vegetation indices have been developed over the past few decades [87]. Each vegetation index is derived by applying unique calculations to the spectral bands of images in ways that enhance different spectral signatures (and thus different characteristics of vegetation) and/or reduce noise from sources such as soil and the atmosphere that can distort remotely–sensed aerial imagery. We chose to consider the utility of a relatively large number of vegetation indices compared to previous SDMs to explore as many different characteristics of vegetation as possible within reason given constraints on available computing resources.

The first vegetation index we chose to include was Normalized Difference Vegetation Index (NDVI). The basic function of NDVI is evaluate the contrast between vegetative canopy cover and non–vegetative cover reflectance using the near–infrared and red spectral bands [88]. It can be used to detect the presence and health of vegetation, estimate biomass, calculate leaf area index (LAI) etc., and measure vegetation productivity while minimizing topographic noise [89, 90]. One weakness of this vegetation index, however, is that it does not account for the invariable background brightness (*e*.*g*., spectral noise) associated with reflections that bounce off non–vegetative surfaces such as soil and particles in the atmosphere within the spectral bands of interest (*ibid*.). As such, we chose to consider Soil–and Atmospherically–resistant Vegetation Index (SARVI), a vegetation index that was specifically designed to reduce the effects of this noise, and thus because it effectively improves vegetation signal sensitivity [91]. Next, we chose to include Green–Blue NDVI (GBNDVI) because it is not as strongly impacted by saturation as NDVI when LAI values are high [92, 93], and it can thus be useful for detecting vegetation [94] and greenspaces [95] in urban areas. We chose to include Blue NDVI (BNDVI) because it is particularly valued for effectively discriminating between pervious and impervious surfaces in urban landscapes [96]. Furthermore, BNDVI is reportedly better at distinguishing vegetation from various other non–vegetative surfaces in urban landscapes than either NDVI or Green–Normalized Difference Vegetation Index (GNDVI) [97]. Lastly, we chose to include GRNDI because it represented a type “vegetation” index (technically, it is actually a simple “spectral” index rather than a true vegetation index [98] because it does not require a near infrared (NIR) band to calculate). This was of interest because not all those interested in creating SDMs will have access to imagery with the NIR band needed to derive so–called true vegetation indices. Although GRNDI might initially seem inferior to other vegetation indices for this reason, GRNDI (as Normalized Difference Green Index, NDGI) is reportedly useful for detecting urban greenspaces in landscapes [95]. The unique capabilities of GBNDVI, BNDVI, and GRNDI made them attractive for our purposes because the Texas Rio Grande Valley Red–crowned Parrots appear to prefer to use certain urban greenspaces (personal observation, Simon Kiacz) that are scattered throughout the greater urban matrix, which is otherwise comprised of relatively large swaths of non–vegetated land cover types (*e*.*g*., impervious surfaces). For example, many of the Washingtonian palm trees (*Washingtonia* spp. [Wendl]) they often prefer to nest in [13] are found in relatively small urban greenspaces that are abutted by paved areas (*e*.*g*., roads, parking lots, sidewalks, etc.) or other man–made structures (*e*.*g*., buildings, etc.).

Each of the vegetation indices considered in this study were derived from National Agricultural Imagery Program (NAIP) images. We downloaded two sets of image tiles for each county in the study area that included an infrared band (*i*.*e*., collected on April, 29 2008 with red, green, blue, infrared bands and April 23 to 30, 2010 with red, green, infrared bands) from the Texas Natural Resource Information System (TNRIS) website [99]. The image tiles were mosaiced using ArcMap to create two continuous images (*i*.*e*., 2008, 2010) consisting of three bands each (*e*.*g*., red, green, blue, infrared) that covered the study area. The three bands from each of these images were exported as individual rasters and used the equations found in Table 1 to calculate five vegetation indices in R Studio: NDVI (2008, 2010), BNDVI (2008), GBNDVI (2008), SARVI (2008), and GRNDI (2008, 2010).

**Table 1:** Equations used to calculate vegetation index values for individual pixels using various spectral bands extracted from National Aerial Imagery Program (NAIP) imagery.

Vegetation Index	Equation
Normalized Difference Vegetation Index (NDVI)	(*NIR*–*Red*)/(*NIR*+*Red*+0.00001)
Blue Normalized Difference Vegetation Index (BNDVI)	(*NIR*–*Blue*)/(*NIR*+*Blue*+0.00001)
Green–Blue Normalized Difference Vegetation Index (GBNDVI)	(*NIR*–(*Green*+*Blue*))/(*NIR*+(*Green*+*Blue*+0.00001))
Green–Red Normalized Difference Index (GRNDI)	((*Green*–*Red*)/(*Green*+*Red*+0.00001))
Soil–and Atmospherically–Resistant Vegetation Index (SARVI)	(1+L) (*NIR*–(*Red*–*RB*)/(*NIR*+*RB*+L))

*NIR*, Near Infrared; RB, *RedBlue*; L, soil conditioning index (improves sensitivity of index by reducing spectral noise caused by soil).

*RB* = *Red*–y(*Blue*–*Red*) where y *=* 1.

L = 0.5.

The nomenclature for this type of feature should be read as follows: “*vegetation index abbreviation”“abbreviated year of imagery collection”*. For example, “*bndvi08*” (S2 Fig) depicts raw BNDVI values derived using bands from the mosaiced 2008 NAIP image.

#### Multitemporal raw vegetation index difference

This novel variable type, a simple vegetation index derivative, contained information about change in raw vegetation index values over the period time. Most terrestrial birds are sensitive to changes in vegetation distributions across the landscapes they occupy (*e*.*g*., patterns such as composition and structure); such changes can have strong impacts on bird diversity and distributions [100]. As with other terrestrial birds [101] including parrots [102], the stability of the vegetation used by Texas Rio Grande Valley Red–crowned Parrots is likely important to their persistence in the region, and as such, we included variables that depicted a measure of change in vegetation over time in our SDMs.

We calculated two types of multitemporal vegetation index difference features. We calculated simple multitemporal vegetation index difference features by subtracting 2008 raw NDVI or GRNDI values (*e*.*g*., *b* = *ndvi08* or *grndi08*) from 2010 raw NDVI or GRNI values (*e*.*g*., *a* = *ndvi10* or *grndi10* [y = *a*–*b*])(104). Normalized multitemporal vegetation index difference features were calculated as y = (*a*–*b*)/(*a*+*b*). Although the simple multitemporal vegetation index difference type features may have been used in SDMs previously [103], the normalized multitemporal vegetation index difference type feature is novel to our knowledge.

The nomenclature for this type of feature should be read as follows: “(n)*delt”* "*abbreviated name of vegetation index” “year two of imagery collection_year one of imagery collection”*. For example, “*deltgrn10_08*” (S3 Fig) depicts the simple difference between 2008 and 2010 raw GRNDI values. Similarly, “*ndeltgrn10_08”* depicts the normalized difference between raw 2008 and 2010 GRNDI values.

#### Percent cover of preferred ranges of raw vegetation index values

This novel variable type, a simple vegetation index derivative, contained information about what portion of an area of a given size was associated with pixels values that fell within certain ranges of raw vegetation index values. To derive it, we first sampled raw vegetation index values for the set of Red–crowned Parrot presence points withheld from modelling. Second, we used Microsoft Excel’s [104] percentile ranking function to determine two different “preferred” ranges of raw vegetation index values. This involved calculating median central tendencies about these presence points. We arbitrarily chose a standard median central range of 50% and a slightly wider central range of 70% to explore how different range sizes affected the utility of variables (S4 Fig). Third, these two ranges were used to develop two separate binary preferred/non–preferred rasters in ArcMap for each vegetation index of interest where a value of “1” was assigned to a pixel in the target raster if the value of its twin pixel in the raw vegetation index raster fell within the “preferred” range and “0” if not.

Animals can have varying functional grains (perceptual scales) of the landscape in relation to different resource patches, such as foraging and nesting and roosting sites [105], making variables defined over different spatial scales valuable for capturing this variability in functional grain for developing SDMs (*e*.*g*., Bellamy et al. [106]). As such, we applied the ArcMap focal statistics tool to the binary preferred/non–preferred rasters to calculate the percent cover of area associated with preferred ranges of raw vegetation index values within two different rectangular focal window sizes: 310 by 310 m (*i*.*e*., 9.61 ha) and 990 by 990 m (*i*.*e*., 98.01 ha) to evaluate the effects of the Red–crowned Parrots’ perception of the overall spread of available potential habitat patches. These window sizes were chosen to explore how the spread of resources might influence habitat suitability.

The nomenclature for this type of feature should be read as “*abbreviated name of vegetation index*” “*abbreviated year of imagery collection*_*median tendency range*_ *size of focal window*”. For example, “*bnd08_70_990*” (S5 Fig) depicts the percentage of pixels around each pixel as a centroid within a 990 m focal window whose values fell within the “preferred” range of raw 2008 BNDVI values (*i*.*e*., central 70% of values about the median value associated with Red–crowned Parrot presence points).

#### Raw vegetation index textures

This variable type, a complex vegetation index derivative, contained information about the texture of raw vegetation index values. Image texture analysis can generally be described as evaluating how the values (*e*.*g*., intensity, brightness of grayscale or color values) of image pixels vary within a moving window analysis [107]. Although image texture analysis can be performed using a variety of either statistically or modelling–based methods [108], we focused on a single statistically–based method, the Grey–Level Co–occurrence Matrix (GLCM). This type of texture analysis involves using a series mathematical functions to calculate how often pairs of pixels with certain values and spatial arrangements within an area of a given size co–occur in an image (a GLCM) and then extracting certain statistical information (*e*.*g*., mean, standard deviation, entropy, contrast, homogeneity, etc.) [109] from the resulting GLCM. The output from each type of statistical analysis can be used to characterize different information about the texture of the image of interest (*i*.*e*., roughness, coarseness, directionality).

We utilized the R package R GLCM Texture package [110] to calculate four GLCM textures (*i*.*e*., 2^nd^–order mean, variance, contrast, and entropy) for each of the vegetation indices of interest using both 310 and 990 m focal windows sizes in R Studio (see Haralick et al. [111] for GLCM texture descriptions and equations used to calculate them). These particular textures were selected to minimize the amount redundant information among predictor variables being entered into models.

The nomenclature for this feature type should be read as “*abbreviated name of vegetation index*” “*abbreviated year of imagery collection*” “*abbreviated name of texture type” “size of focal window*”. For example, “*bnd082c990*” (S6 Fig) depicts the GLCM 2^nd^–order contrast texture of raw 2008 BNDVI values for each pixel as a centroid of a 990 by 990 m focal window.

#### Texture of binary preferred/non–preferred ranges of raw vegetation index values

This novel variable type, a complex vegetation index derivative, contained information about different patterns in binary preferred/non–preferred ranges of raw vegetation index values. We used the GLCM Texture package to calculate 2^nd^–order GLCM mean, variance, contrast, and entropy textures of the binary preferred/non–preferred raw vegetation index rasters (described above) using square 310 and 990 m focal window sizes in R Studio.

The nomenclature for this type of feature should be read as “*abbreviated name of vegetation index*” “*abbreviated year of imagery collection*” “*abbreviated name of texture type” “size of focal window*” “*median tendency range”*. For example, “*bnd082m31070*” (S7 Fig) depicts the GLCM 2^nd^–order mean texture of the 70% median central tendency raw 2008 BNDVI binary preferred/non–preferred range raster of each pixel as a centroid of a square 310 by 310 m focal window.

#### Percent cover of preferred range of vegetation index texture values

This novel variable type, a complex vegetation index derivative, contained information about what portion of an area of a given size was associated with pixels values that fell within certain ranges of raw vegetation index texture values. We derived it using a process similar to that used to create percent cover of preferred ranges of raw vegetation index values type features (see above). We experimented with several median central tendency ranges (*i*.*e*., 25 to 95% of values associated with parrot presence about the median value) and ultimately selected two to four ranges (*i*.*e*., 70%, 80%, 85%, and/or 90%). Our selection of these particular ranges was somewhat subjective but was driven in part by a visual estimation of the approximate threshold that separated preferred from non–preferred ranges of values.

The nomenclature for this type of feature should be read as “*abbreviated name of vegetation index*” “*abbreviated year of imagery collection*” “*abbreviated name of texture type” “abbreviated size of focal window*” “*median tendency value”*. For example, “*bnd082c9985*” (S8 Fig) depicts the percentage of pixels around each pixel as a centroid in a square 990 by 990 m window whose values fell within the “preferred” range of 2^nd^–order GLCM contrast texture of raw 2008 BNDVI values (*i*.*e*., central 85% of values about the median value associated with Red–crowned Parrot presence).

#### Feature selection and ranking

Rather than relying on single subset of highly–ranked individual features to create a final SDM (an approach commonly used to develop SDMs [112, 113]), we opted to use the RSFSA approach, which involves selecting multiple random feature subsets that produce higher performing models and then randomly selecting a set of these models to create a consensus ensemble (described below) that represents the final SDM [69]. One benefit of the RSFSA approach is that it allows users to explore the utility of different variables in terms of their synergistic value in certain feature subsets. Other SDM methods that involve creating a single model from a single subset of variables might exclude certain variables that perform poorly in some feature subsets or by themselves without being aware that they may work synergistically with other variables in different feature subsets (*e*.*g*., Guyon and Elisseeff [114]). Another benefit of the RSFSA approach is that it limits collinearity via the use of a correlation filter prior to generating the original feature subsets. Sets of variables with a level of correlation greater than a specific threshold, which was set to a more restrictive level of |0.5| rather than the typical |0.7| for this study (*i*.*e*., feature subsets were composed of features that were less correlated to each other), cannot co–occur in any given feature subset.

**Fig 4:**
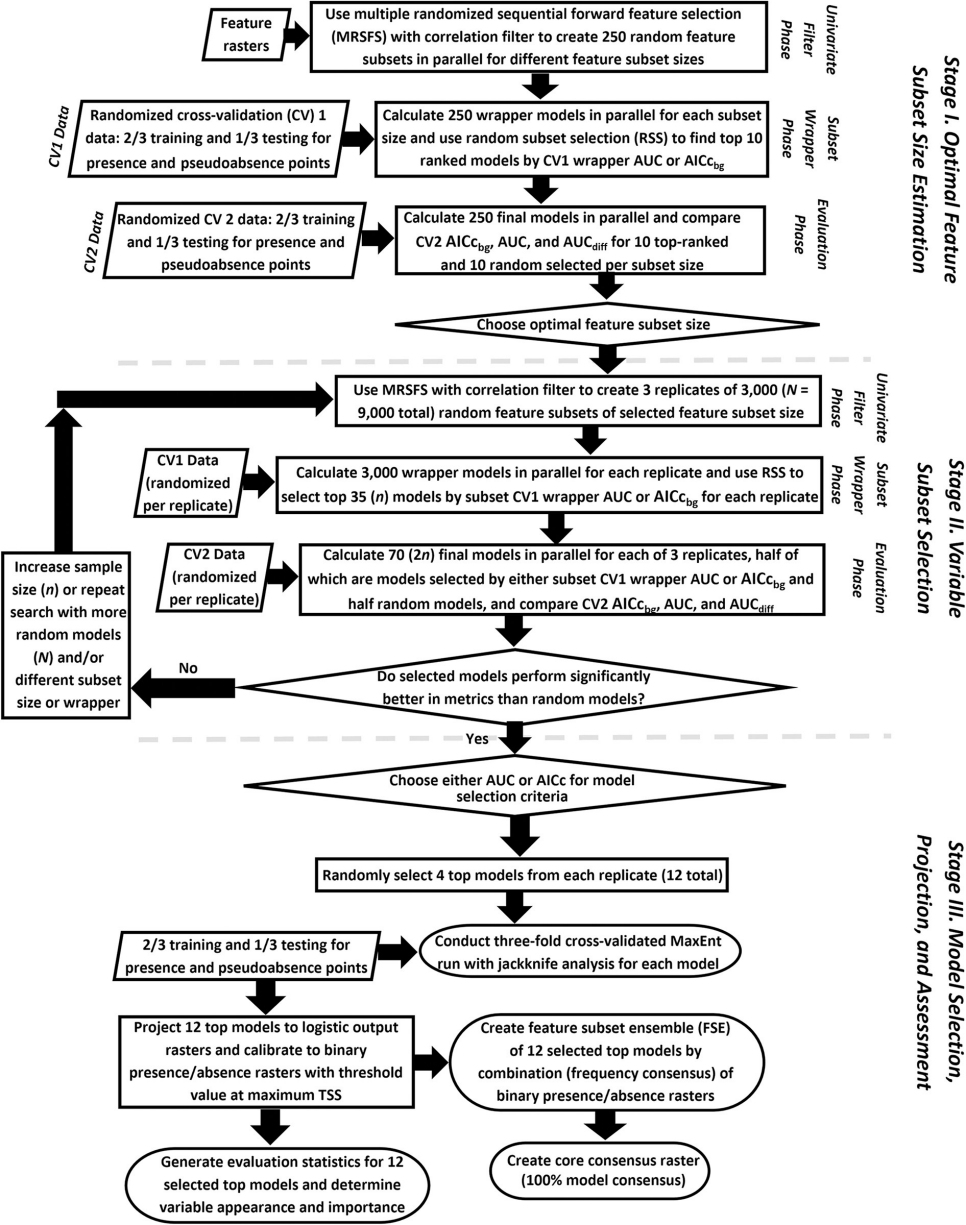
A workflow diagram for the Cross–Validated Random Subset Feature Section Algorithm (RSFSA–CV) was used to develop the habitat distribution models in this study. The basic procedures used to create the three predictive MaxEnt habitat distribution models for the Texas Rio Grande Valley Red–crowned Parrots. See Tracy et al. [69] for more detailed information about this method.

We employed an updated version of the RSFSA used in Tracy et al. [69], the cross–validated RSFSA (RSFSA–CV) [115] in this study. The primary difference is that RSFSA–CV involves re–randomizing the presence and pseudoabsence data sets to cross–validate both the training and testing data sets in the subset wrapper and evaluation phases (Fig 4). Presence data can thus be used more efficiently, which is especially important when using smaller presence data sets such as was the case with our nest site and roost site presence data sets.

The MaxEnt RSFSA–CV has three primary stages (Fig 4). In the first stage, 250 models are run using the rapid MaxEnt Samples with Data (SWD, non-raster producing) format for various variable subset sizes (one to ten variables) to find the top ten performing variable subsets (compared to randomly–selected models) of a given subset size. In both the first and second stages, model performance is evaluated using three criteria: (1) higher accuracy as indicated by pseudoabsence AUC (AUC_psa;_ calculated with pseudoabsence points as absences), (2) lower complexity and higher information content as indicated by background corrected Akaike Information Criterion (AICc_bg_, calculated using background points) [116], and lower overfitting as indicated by pseudoabsence difference AUC, which is calculated by subtracting pseudoabsence test AUC from pseudoabsence training AUC (AUC_psa___diff_ = AUC_psa_train_–AUC_psa_test_) [69, 117]. The lowest subset size producing high performance models is then chosen for the second stage of RSFSA-CV. In the second stage, three replicates of about 3,000 MaxEnt models each of the optimal variable subset size are run with SWD format to identify the top 250 of 3,000 models compared to 250 randomly–selected models according to the three above evaluation criteria (Welch’s t–test; α = 0.05). The higher performing selection method (e.g., AUC_psa_ or AICc_bg_) is used to rank the 250 top models in each of the three replicates. In the third stage, four of the 250 top-ranked models from each of the three replicates (750 models total) are chosen to create a feature subset ensemble consisting of 12 models (these 12 models are not necessarily better performing than any of the other 750 selected models). The 12 top–selected models are run using SWD format in MaxEnt evaluation mode (k–fold cross–validation; *k =* 10) to produce variable response curves and derive final metrics to assess model performance (*i*.*e*., AUC_psa_, background presence AUC [AUC_bgp_, calculated using background and presence points as absences], AUC_psa_diff_, AICc_bg_, pseudoabsence True Skill Statistic [TSS_psa_, calculated using pseudoabsence points as absences], and background True Skill Statistic [TSS_bg_, the MaxEnt default; *c*.*f*., Tracy et al. [69]]). The top-selected 12 models are projected to develop a feature subset ensemble model (see below). Finally, the performance of predictor features in the top 250 models from all three RSFSA-CV replicates is ranked (see below) and the effects of top–ranking variables on the probability of species presence are evaluated using their response curves (*i*.*e*., individual, marginal). A marginal response curve is a graph–based depiction of MaxEnt cloglog output for probability of presence (y–axis) plotted against the values of the given predictor variable values vary (x–axis) while the other predictor variable values are kept at their average sample value [81].

#### Variable ranking

We used the updated methodology of Tracy et al. [115] to rank variables. Variable performance was ranked using weights of 60% and 40% for MaxEnt model mean permutation importance (*i*.*e*., novel information contributed by a given variable added to each of the 250 top–performing models it appeared in over three replications) and total frequency of appearance in models (*i*.*e*., how often the variable appeared in the 250 top–performing models), respectively. While both of these measures are important for evaluating variable performance, we gave more weight in the rankings to mean permutation importance as it is somewhat more relevant. We reported the ranking and correlation group number of each set of predictor variables (top 30) associated with their respective set of 250 top–performing models. Variable correlation groupings were formed under top ranking variables to include other closely ranked variables correlated at |0.5| or higher by placing variables in multiple correlation groups where they have the closest ranking below the top ranked group member.

### Feature subset ensembles

The 12 top–selected models for each habitat distribution selected using the RSFSA-CV (see above) were calibrated to binary presence/absence format using a threshold of maximum TSS_psa_ [118]. These models were then used to create a series of feature subset ensembles (FSE; frequency of consensus between all 12 of the binary presence/absence calibrated MaxEnt models) from which our final projected habitat distributions (maps) were derived. The color red in the FSE frequency consensus map indicated agreement among all 12 models, with other colors showing a lower level of agreement, indicating lower levels of confidence that they are actually part of the true habitat distribution. In addition to creating a frequency of consensus projection for each habitat distribution of interest, we used the calibrated binary presence/absence MaxEnt models to create “core” and “semi–core” projections. The core maps show the predicted habitat distributions as determined according to areas where there was 100% and 50% or greater agreement between the 12 top–selected models, respectively. Note that while the information depicted by these maps would change somewhat if a different set of randomly selected top-performing models was used to create the feature subset ensembles, the frequency of consensus method captures provides a reasonable snapshot of this variability.

### Habitat distribution extents and land–use/land–cover compositions

ArcMap was used to calculate the areal extents of the predicted general use, nest site, and roost site habitat distributions as identified by the core and semi–core projections. An LULC map for the State of Texas was downloaded from the Texas Parks and Wildlife Department (TPWD) website [119] and resampled to 9.5 m. The percentages of the LULC types that overlapped the areas that made up each of the core projections were also calculated in ArcMap.

## Results

### Suitable general use habitat distribution

#### Feature selection performance

**Fig 5:**
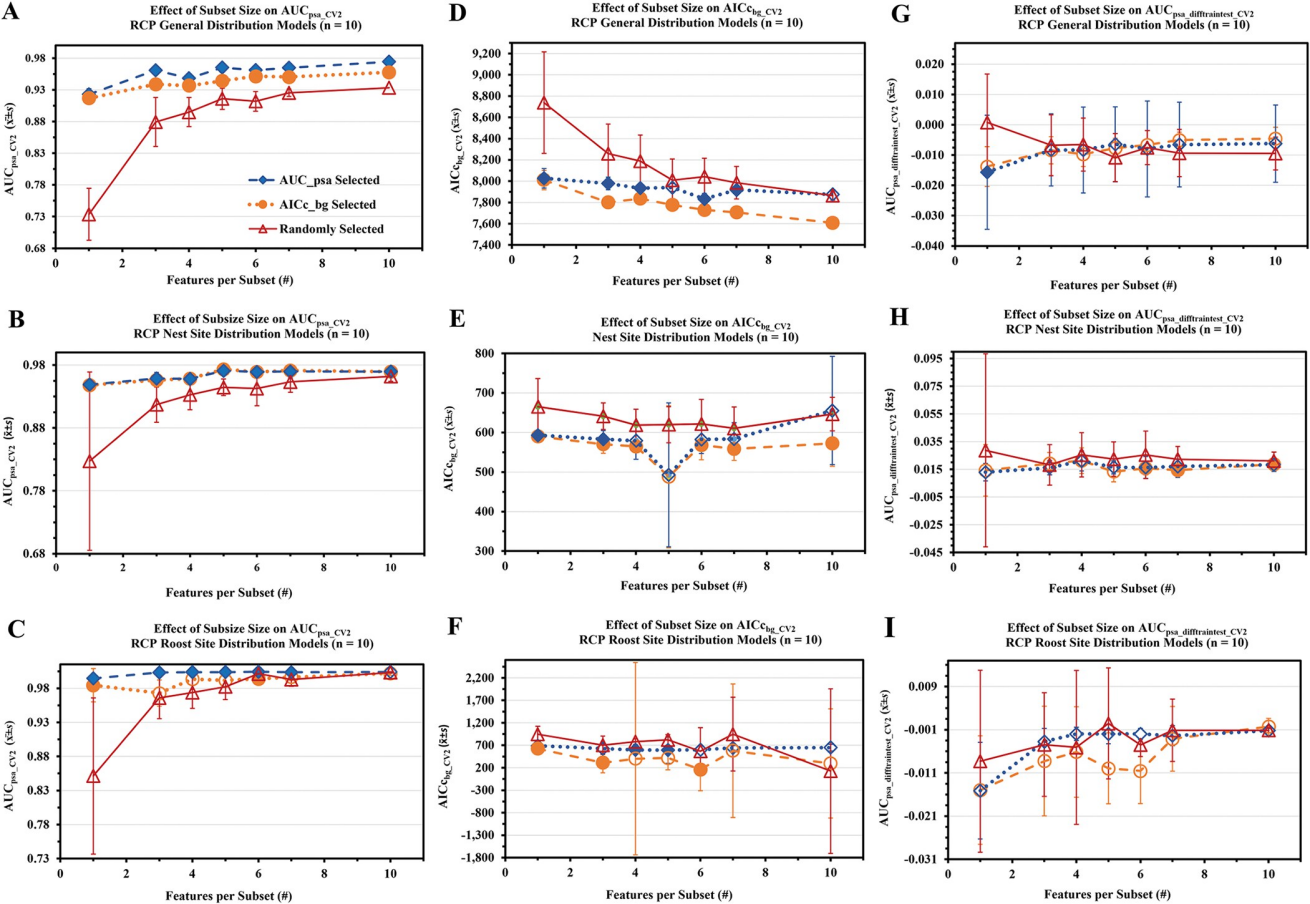
Stage I results of the MaxEnt version of the Cross–Validated Random Subset Feature Selection Algorithm (RSFSA–CV). Texas Rio Grande Valley Red–crowned Parrot general use (A, D, G), nest site (B, E, H), and roost site (C, F, I) habitat distribution models were derived from top ten performing AUC_psa_−or AICc_bg_–selected feature subsets compared to ten random subsets out of 250 randomly generated subsets as determined using the RSFSA–CV. Model performance evaluation statistics shown include: AUC_psa_CV2_ (A to C), AICc_bg_CV2_ (D to F), and AUC_psa_diff_CV2_ ((G to I)). Filled markers indicate AICc_bg_−or AUC_psa_–selected models performed significantly better than randomly selected models (Welch’s t–test, *p* < .05).

**Fig 6:**
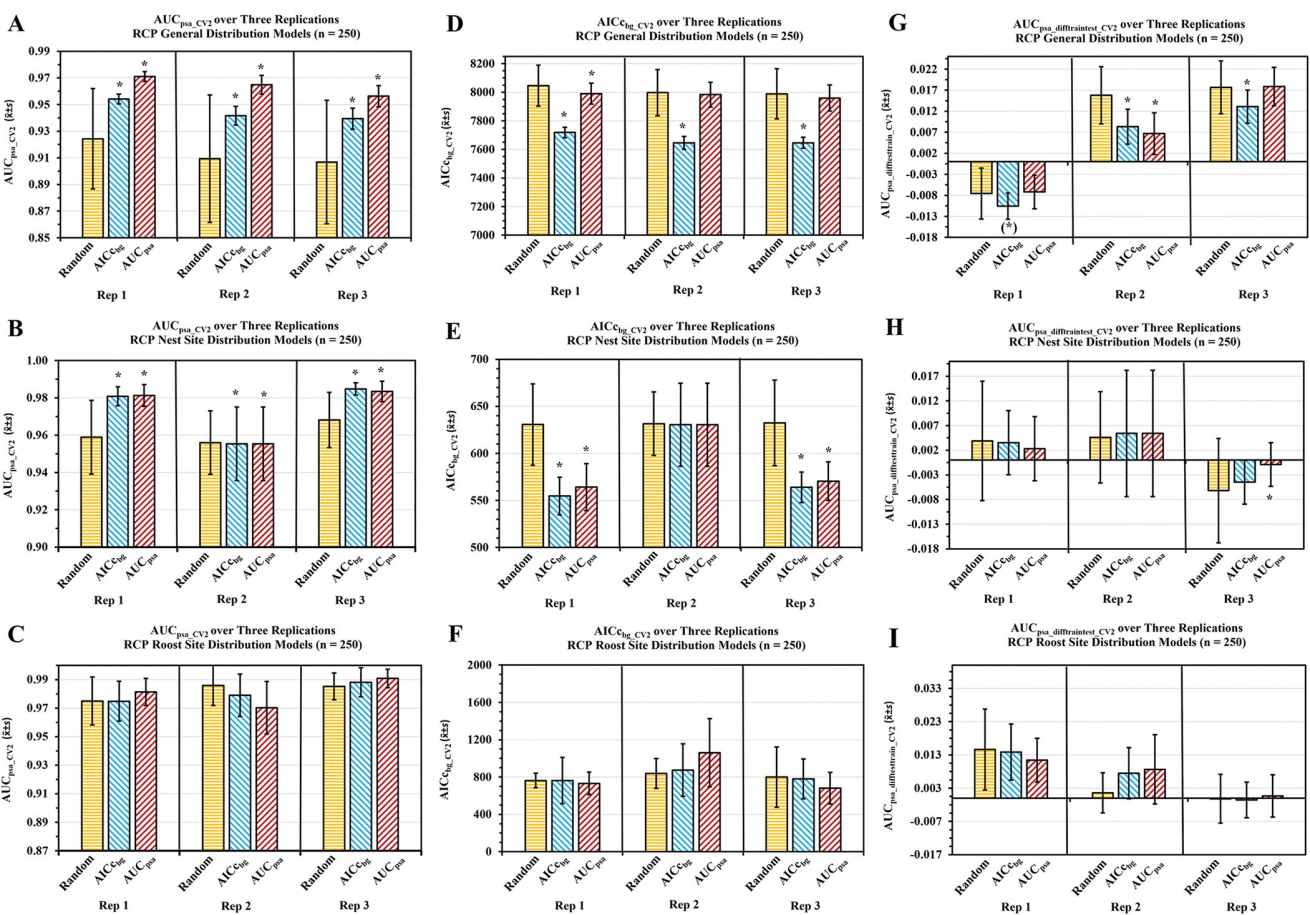
Stage II results of the MaxEnt version of the Cross–Validated Random Subset Feature Selection Algorithm (RSFSA–CV). Top AUC_psa_−or AICc_bg_−selected 250 Texas Rio Grande Valley Red–crowned Parrot general use (A, D, G; seven variable feature subsets), nest site (B, E, H; five variable feature subsets), and roost site (C, F, I; four variable feature subsets) habitat distribution models performance compared to randomly selected models as determined via the RSFSA–CV (Welch’s t–test, *p* < .05). Performance determined using mean evaluation statistics ($\bar{x}$±*s* of AUC_psa_CV2_ (A to C), AICc_bg_CV2_ (D to F), AUC_psa_diff_CV2_ (G to I)); 3,000 feature subsets per three training replicates. Asterisks indicate AICc_bg_−or AUC_psa_–selected models performed significantly better than randomly selected models (Welch’s t–test, *p* < .05).

A feature subset size of seven variables with a model selection criterion of AICc_bg_ was optimal for producing high performing models (Figs 5 and 6). The 12 top–selected models had high accuracy with an AUC_psa_ of 0.942±0.005, which was markedly higher than the MaxEnt default AUC_bgp_ (*i*.*e*., 0.904±0.004). A similar trend was observed with TSS (*i*.*e*., TSS_psa_ = 0.779±0.013 *vs*. TSS_bgp_ = 0.716±0.013; Table 2).

**Table 2:** Stage III results from the Maxent Cross–Validated Random Feature Subset Selection Algorithm (RSFSA–CV) for Texas Rio Grande Valley Red-crowned Parrots.

Habitat Distribution FSE	Selection Criteria	Data Used	Feature Subset Size	Number of Features Used		True Skill Statistic		Area Under the Curve	
*Type*	*Type*	*Type*	*#*	$\bar{x}$	*s*	$\bar{x}$	*s*	$\bar{x}$	*s*
General Use	AICc_bg_	Test_psa_	7	6.75	0.452	0.779	0.0130	0.942	0.005
General Use	AICc_bg_	Test_bpg_	7	6.75	0.452	0.716	0.0130	0.904	0.004
Nest Site	AICc_bg_	Test_psa_	5	4.917	0.289	0.889	0.062	0.969	0.019
Nest Site	AICc_bg_	Test_bpg_	5	4.917	0.289	0.883	0.059	0.965	0.018
Roost Site	AICc_bg_	Test_psa_	4	3.833	0.39	0.86	0.06	0.967	0.019
Roost Site	AICc_bg_	Test_bpg_	4	3.833	0.39	0.85	0.06	0.962	0.019

$\bar{x}$, arithmetic mean; *s*, standard deviation of the mean; AICc, corrected Akaike information criterion; psa, pseudoabsence; bg, background; bpg, background presence; FSE, feature subset ensemble.

The average performance over three replications of the final 12 top–selected models used to predict general use, nest site, and roost site habitat distributions.

#### Variable rankings in 250 top–performing models

The top 15 ranked variables in the feature selected general use habitat models were dominated by percent cover of preferred ranges (*i*.*e*., 70%) of 2^nd^–order GLCM entropy and contrast textures of raw 2010 NDVI values (*ndv102e99pc70*) followed by 2^nd^–order GLCM entropy and contrast textures of raw 2010 NDVI values derived using a 990 m focal window (*ndv102e990*, *ndv102c990*; Table 3; S1 Table). Probability of parrot occurrence increased gradually in a manner resembling a convex curve from low to high values of *ndv102e99pc70*, but the response curve was mostly flat over low and medium values of *ndv102e990*, increasing sharply in a sigmoidal fashion at the highest presence values (S9 Fig). *Elevation* occurred among the list of the top five top–ranked variables (Table 3). The probability of parrot occurrence peaked at low values of *elevation*, sharply decreasing in a manner resembling a concave curve to zero at relatively medium to high elevations (*i*.*e*., 30 to 50 m; S9 Fig). Variables ranked from ten to 20 were dominated by 2^nd^–order entropy textures of raw 2008 GBNDVI, BNDVI, and NDVI derived using both focal window sizes (*e*.*g*., *ndv082e990*; Table 3). The probability of parrot occurrence exhibited a gradual sigmoid increase to high values of *ndv082e990* (S9 Fig). Raw vegetation indices, including raw 2010 NDVI and GRNDI and multitemporal vegetation index differences variables (*i*.*e*., *deltgrn10_08*), ranked poorly and generally had a low mean permutation importance; however, they did occasionally appear in top–performing models (S9 Fig and S2 Table). Additional information about variables in the 12 top–selected models can be found in the Supporting Information section (S1 and S2 Tables).

**Table 3:** The 30 top–ranked variables that occurred in feature subsets of each of the 250 top–performing models used to derive the general use, nest site, and roost site habitat distribution projections for Texas Rio Grande Valley Red–crowned Parrots.

Variable Rank	*Variable_Name* ^*a*^		
	(Variable Correlation Group Number)^b^		
	[Variable Occurrence in 12 Top–Selected Models]		
	General Use Habitat	Nest Site Habitat	Roost Site Habitat
	*7 Variables per Model* ^*c*^	5 V*ariables per Model*^*c*^	4 V*ariables per Model*^*c*^
1	*ndv102e99pc70* (1)[4]	*ndv102e310* (1)[0]	*ndv102c99pc85* (1)[1]
2	*ndv102e990* (2)[3]	*gbn082e310* (2)[1]	*ndv102c990* (1)[0]
3	*ndv102c990* (2)[0]	*ndv102e990* (1)[0]	*ndv102e99pc70* (1)[0]
4	*ndv102e99pc80* (1)[2]	*ndv102e99pc80* (3)[0]	*grn102e310* (2)[0]
5	*elevation* (3)[5]	*ndv102e99pc70* (3)[1]	*ndv102c310* (2)[0]
6	*ndv102c99pc70* (1)[1]	*elevation* (4)[1]	*ndv102c99pc90* (1)[0]
7	*ndv102c99pc85* (1)[1]	*ndv102e31pc70* (1)[1]	*grn102m99pc70* (3)[0]
8	*ndv102c99pc90* (2)[1]	*bnd082e310* (2)[1]	*grn102c31070* (4)[2]
9	*ndv102e310* (2)[0]	*ndv102c99pc70* (3)[0]	*bnd082c310* (5)[1]
10	*gbn082e990* (4)[0]	*ndv102c99pc85* (3)[1]	*grn102c99pc90* (6)[0]
11	*bnd082e990* (4)[0]	*ndv102c99pc90* (3)[1]	*ndv102c99pc70* (1)[0]
12	*ndv102m310* (5)[0]	*ndv082e310* (2)[1]	*ndv102e310* (5)[0]
13	*ndv102m990* (5)[1]	*ndv102c990* (1)[0]	*grn102m31pc70* (3)[1]
14	*ndv102c99070* (6)[0]	*ndv102c31pc70* (5)[0]	*ndv102e99070* (7)[0]
15	*ndv082e990* (4)[1]	*ndv082e990* (2)[0]	*grn102v31pc70* (8)[1]
16	*bnd082e310* (4)[0]	*sar082e990* (6)[0]	*bnd082e310* (5)[0]
17	*gbn082e310* (4)[0]	*sar082c310* (6)[0]	*gbn082c99pc70* (9)[0]
18	*ndv102v990* (5)[2]	*gbndvi08* (7)[1]	*ndv102c31070* (7)[0]
19	*sar08_50_990* (7)[0]	*gbn082v310* (7)[0]	*grn102c990* (2)[1]
20	*sar082m99070* (7)[1]	*bnd082e99pc70* (8)[0]	*ndv102m99pc70* (10)[0]
21	*ndv102v310* (5)[0]	*ndv082m990* (7)[0]	*grn102e31070* (4)[0]
22	*bnd082c99070* (4)[0]	*gbn082e990* (8)[1]	*grn10_70_990* (11)[0]
23	*gbn082e99pc80* (4)[0]	*ndvi08* (7)[0]	*grn102v99pc70* (3)[0]
24	*sar082e99pc80* (8)[0]	*bnd082e99pc80* (8)[0]	*bnd082c99pc70* (9)[1]
25	*ndv102e31pc70* (2)[0]	*sar082m31pc70* (9)[1]	*ndv102v99pc70* (10)[0]
26	*sar08_50_310* (7)[0]	*sar082e310* (6)[0]	*ndv102c31pc70* (1)[0]
27	*sar082v99070* (7)[0]	*sar082e99pc80* (6)[1]	*gbn082e310* (5)[0]
28	*grn102c99070* (6)[1]	*sar08_50_990* (10)[0]	*ndv102e31070* (7)[0]
29	*ndv082v990* (9)[1]	*ndv082c990* (8)[0]	*bnd082c990* (9)[0]
30	*sar082c99pc70* (8)[0]	*bnd082e99070* (11)[0]	*gbn082c990* (9)[0]
**Percent of top 30 variables from all 250 top–performing models occurring in 12 top–selected models**			
	43% (13/30)	40% (12/30)	23% (7/30)

Ranks determined using percent mean permutation importance (60% weight) and frequency of occurrence (40% weight) over three replications (3,000 models each). Shaded cells denote variables occurring in at least one of the 12 top–selected models used to derive final projections.

^a^See S1 Table in Supporting Information section for variable nomenclature.

^b^Variables within a correlation group cannot co-occur in a feature subset (see Methods).

^**c**^Optimal number of variables per model type determined via feature selection (see Results).

### Predicted extent and LULC composition

**Fig 7:**
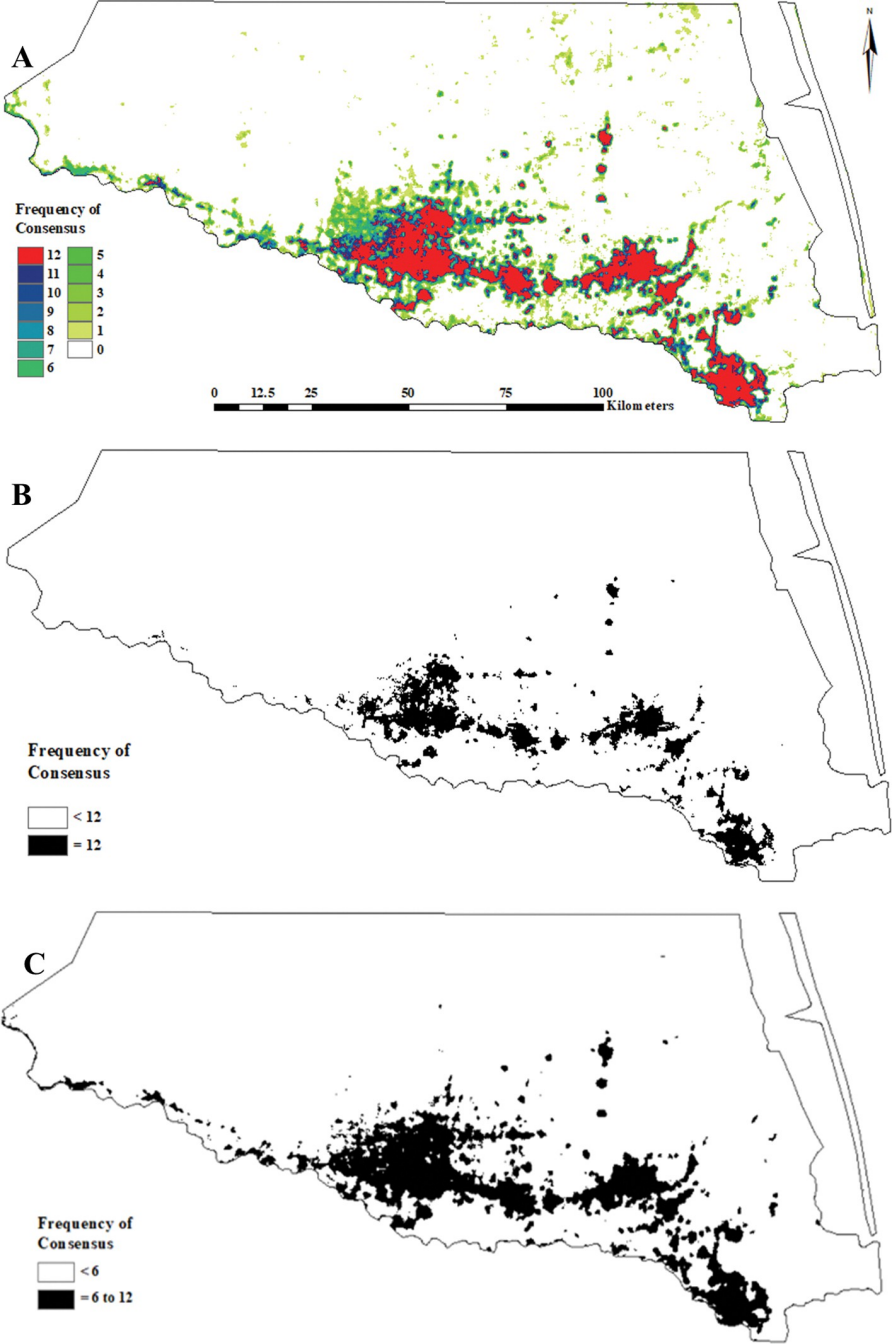
Texas Rio Grande Valley Red–crowned Parrot predicted general use habitat distribution as determined via a consensus-based feature ensemble of 12 calibrated (binary presence/absence) MaxEnt models. (A) complete graduated (frequency of consensus between the 12 top-selected models), (B) 100% consensus (“core” = 12 models), and (C) 50% consensus (“semi–core” ≥6 models).

The areal extents of the general use habitat distribution core and semi–core projections were 546.5 and 1195.1 km^2^, respectively (Fig 7). The LULC types associated with the area encompassed by the core projection were: *Low Urban*–53.9%, *Disturbance Grassland*–15.4%, *High Urban*–12.0%, *Row Crops*–5.7%, *Tamaulipan Mixed Deciduous Thornscrub* (*i*.*e*., *Clayey Blackbrush Mixed Shrubland*)–3.0%, *Sandy Mesquite Dense Shrubland*–2.1%, *Open Water*–1.1%, and others–<1.0%.

### Suitable nest site habitat distribution

#### Feature selection performance

Five–variable subsets with a selection criterion of AICc_bg_ were optimal for producing high performing models (Figs 5 and 6). High accuracy in the 12 top–selected models was reflected in both AUC measures (*i*.*e*., AUC_psa_ = 0.969±0.019, AUC_bgp_ = 0.965±0.018). The two TSS measures were approximately equal (*i*.*e*., TSS_psa_ = 0.889±0.062; TSS_bgp_ = 0.883±0.05; Table 2).

#### Variable rankings in 250 top–performing models

The top 15 ranked variables in feature selected nest site habitat distribution models were dominated by six feature type correlation groups (correlation group number): (1) 2^nd^–order GLCM entropy and contrast textures of raw 2010 NDVI values derived using both focal window sizes and precent cover of preferred ranges of 2^nd^–order GLCM entropy textures of raw 2010 NDVI values (*i*.*e*., 70/85/90%) derived using a 310 m focal window (*e*.*g*., *ndv102e31pc70*); (2) 2^nd^–order entropy textures of raw 2008 GBNDVI, BNDVI, and NDVI values derived using both focal window sizes (*e*.*g*., *gbn082e310*); (3) percent cover of preferred ranges (*i*.*e*., 70%) of 2^nd^–order GLCM entropy and contrast textures of raw 2010 NDVI values derived using a 990 m focal window; and (4) *elevation* (Table 3). The probability of parrot occurrence increased gradually in a convex curve from low to high values of *ndv102e31pc70*, but it increased in a gradual sigmoidal fashion at higher values of *gbn082e310* (S10 Fig). Two raw 2008 vegetation indices, GBNDVI and NDVI, occurred among the list of the top 30 variables; however, this type of variable, along with the even lower ranked multitemporal vegetation index difference type variables (*e*.*g*., *deltgrn10_08*) had relatively low mean permutation importance whenever they did appear in our 12 top–selected models (Table 3). Additional information about variables in the 12 top–selected models can be found in the Supporting Information section (S1, S2 Tables).

#### Predicted extent and LULC composition

**Fig 8:**
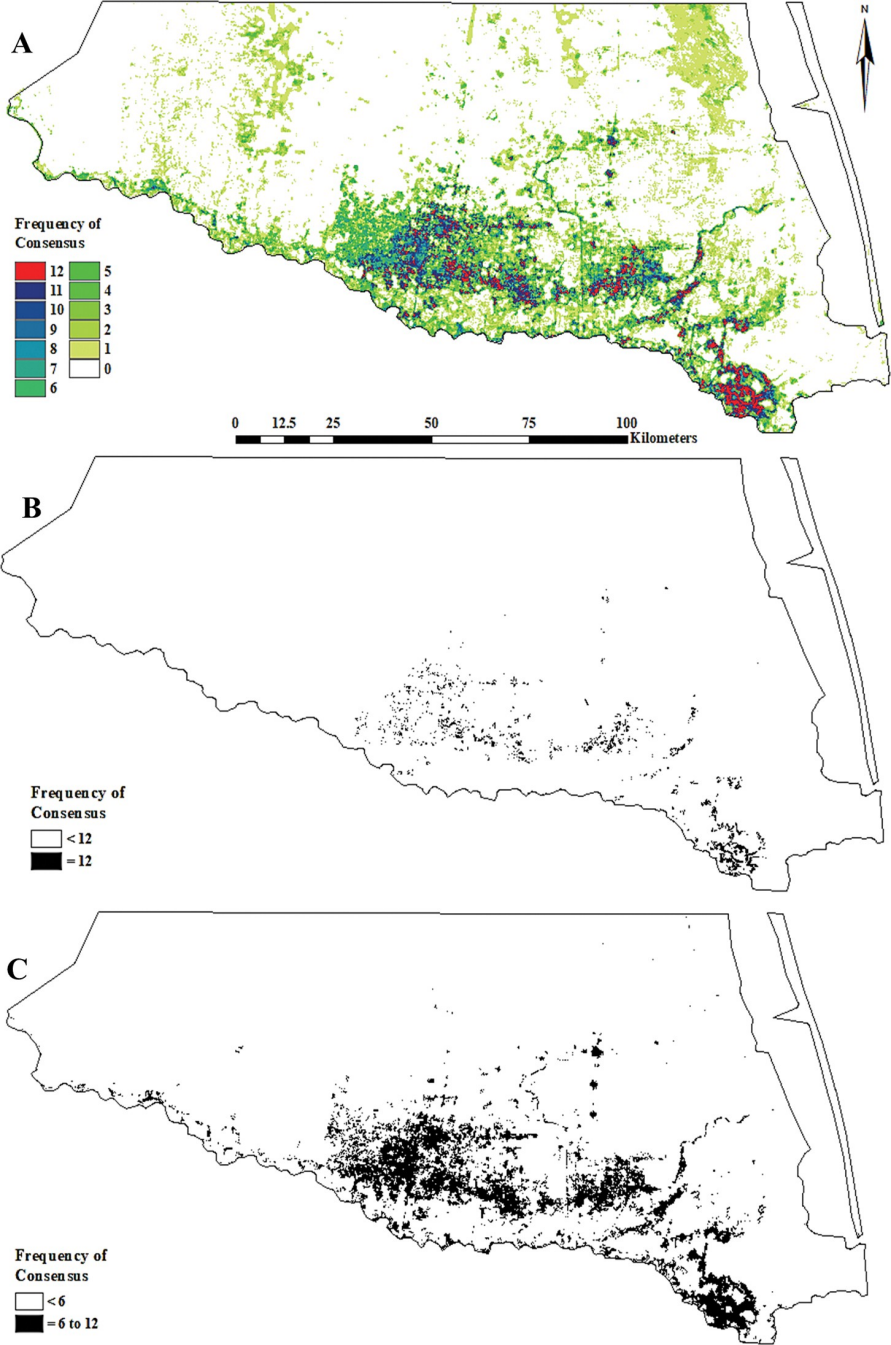
Texas Rio Grande Valley Red–crowned Parrot predicted nest site habitat distribution as determined via a consensus-based feature ensemble of 12 calibrated (binary presence/absence) MaxEnt models. (A) complete graduated (frequency of consensus between the 12 top-selected models), (B) 100% consensus (“core” = 12 models), and (C) 50% consensus (“semi–core” ≥6 models).

The areal extents of the predicted nest site habitat distribution core and semi–core projections were 118.2 and 871.1 km^2^, respectively (Fig 8). The LULC types associated with the core projection were: *Low Urban*–61.6%, *Disturbance Grassland*–13.6%, *Tamaulipan Mixed Deciduous Thornscrub* (*i*.*e*., *Clayey Blackbrush Mixed Shrubland*)–5.2%, *High Urban*–4.0%, *Sandy Mesquite Dense Shrubland*–3.6%, *Row Crops*–2.7%, *Floodplain Evergreen Forest and Woodland*–1.2%, *Floodplain Herbaceous Wetland*–1.2%, *Orchard*–1.0%, and others–1.0%.

### Suitable roost site habitat distribution

#### Feature selection performance

Feature subsets of four variables and a model selection criterion of AICc_bg_ was optimal for identifying high performing models of the roost site habitat distribution (Figs 5 and 6). High accuracy in the 12 top–selected models was reflected in values of both AUC_psa_ and AUC_bgp_ at 0.967±0.019 and 0.962±0.019, respectively. The two TSS measures were approximately equal (*i*.*e*., TSS_psa_ = 0.86±0.06, TSS_bgp_ = 0.85±0.06; Table 2).

#### Variable rankings in 250 top–performing models

The 15 top–ranked variables in feature selected roost site habitat distribution models were dominated by percent cover of preferred ranges of 2^nd^–order GLCM entropy and contrast textures of raw 2010 NDVI values (*ndv102c99pc85*, *ndv102e99pc85*) and 2^nd^–order GLCM contrast texture of raw 2010 NDVI (*ndv102c990*; Table 3). The probability of parrot presence increased gradually from zero to peak levels at a moderate value of *ndv102c99pc85* before gradually declining in a concave fashion at high values (S11 Fig). Other highly ranked variable types in the 30 top–ranked variables included 2010 2^nd^–order GLCM entropy and contrast textures of raw GRNDI and NDVI values derived using a 990 m focal window (*e*.*g*., *grn102c990*) and percent cover of preferred (central 70%) range of 2^nd^–order GLCM mean textures of raw 2010 GRNDI values derived using both focal window sizes (*e*.*g*., *grn102m31pc70*; Table 3). The probability of parrot occurrence sharply peaked at a lower value for *grn102c990* and gradually declined to zero at a medium value. The probability of occurrence slowly increased in a concave fashion before peaking at high values for *grn102m31pc70* (S11 Fig). Four percent cover of preferred ranges of raw vegetation index values type features, such as *ndv08_50_310*, appeared in the 12 top–selected models, but they had medium to low mean permutation importance and generally ranked lower overall (Table 3; S2 Table). Raw vegetation index type features (*e*.*g*., *grndi08*, *gbndvi08*) and multitemporal vegetation index difference type features (*e*.*g*., *deltndvi10_08*) were also low ranking, and their mean permutation importance ranged from zero to mid–range values (S2 Table). They did appear in some of our 12 top–selected models (S1, S2 Tables). Additional information about variables in the 12 top–selected models can be found in the Supporting Information section.

#### Predicted extent and LULC composition

**Fig 9:**
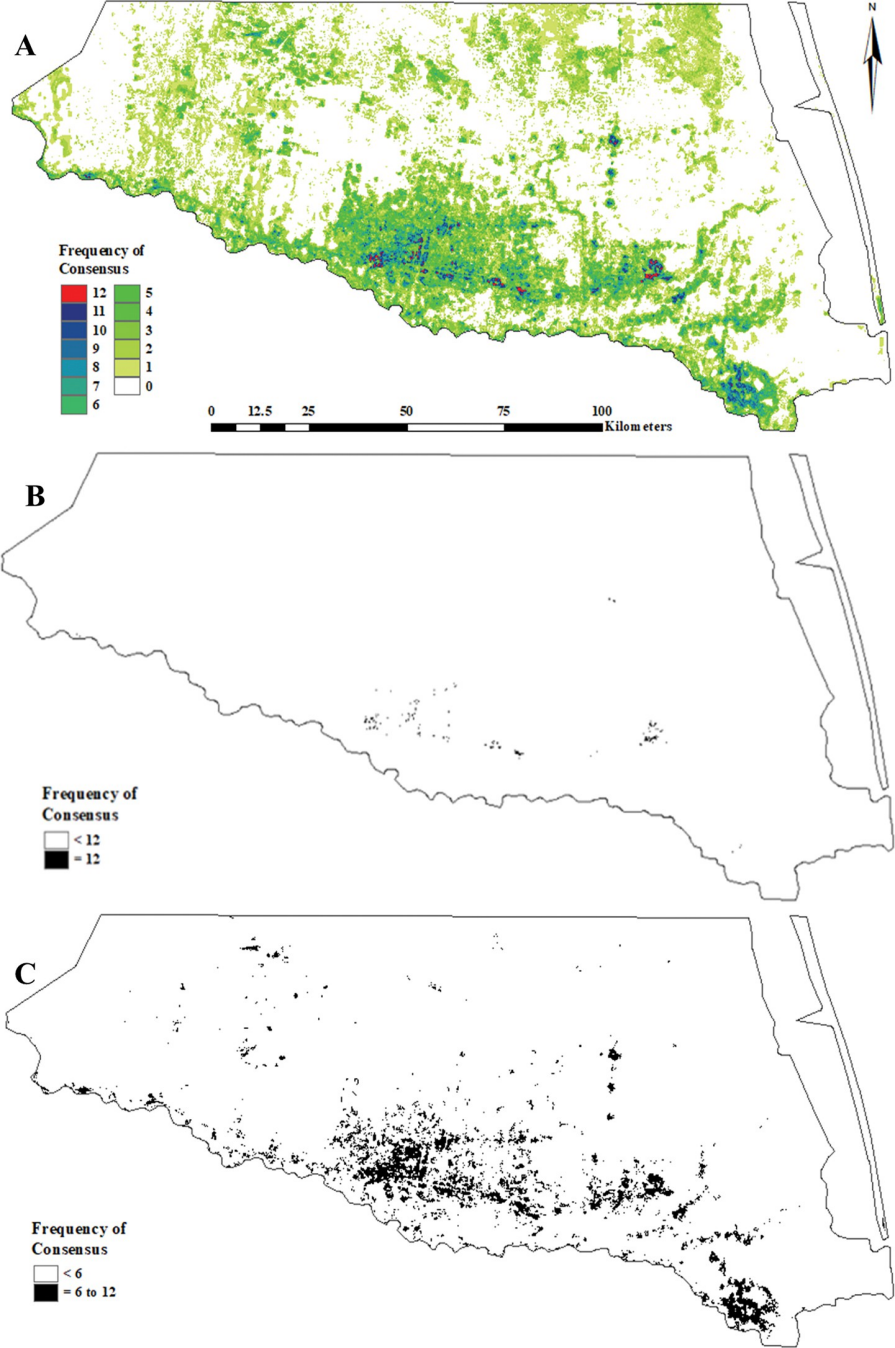
Texas Rio Grande Valley Red–crowned Parrot predicted roost site habitat distribution as determined via a consensus-based feature ensemble of 12 calibrated (binary presence/absence) MaxEnt models. (A) complete graduated (frequency of consensus between the 12 top-selected models), (B) 100% consensus (“core” = 12 models), and (C) 50% consensus (“semi–core” ≥6 models).

The areal extents of the roost site habitat distribution core and semi–core projections were 10.4 and 494.4 km^2^, respectively (Fig 9). The LULC types associated with the area encompassed by the core projection were: *Low Urban*–97.3%, *High Urban*–1.8%, and various others–0.9%.

### Overlap and differences between suitable habitat distributions

The combined areal extent of the core and semi core projections of the general use, nest site, and roost site habitat distributions was 1583.0 km^2^ and 5840.5 km^2^, respectively.

**Fig 10:**
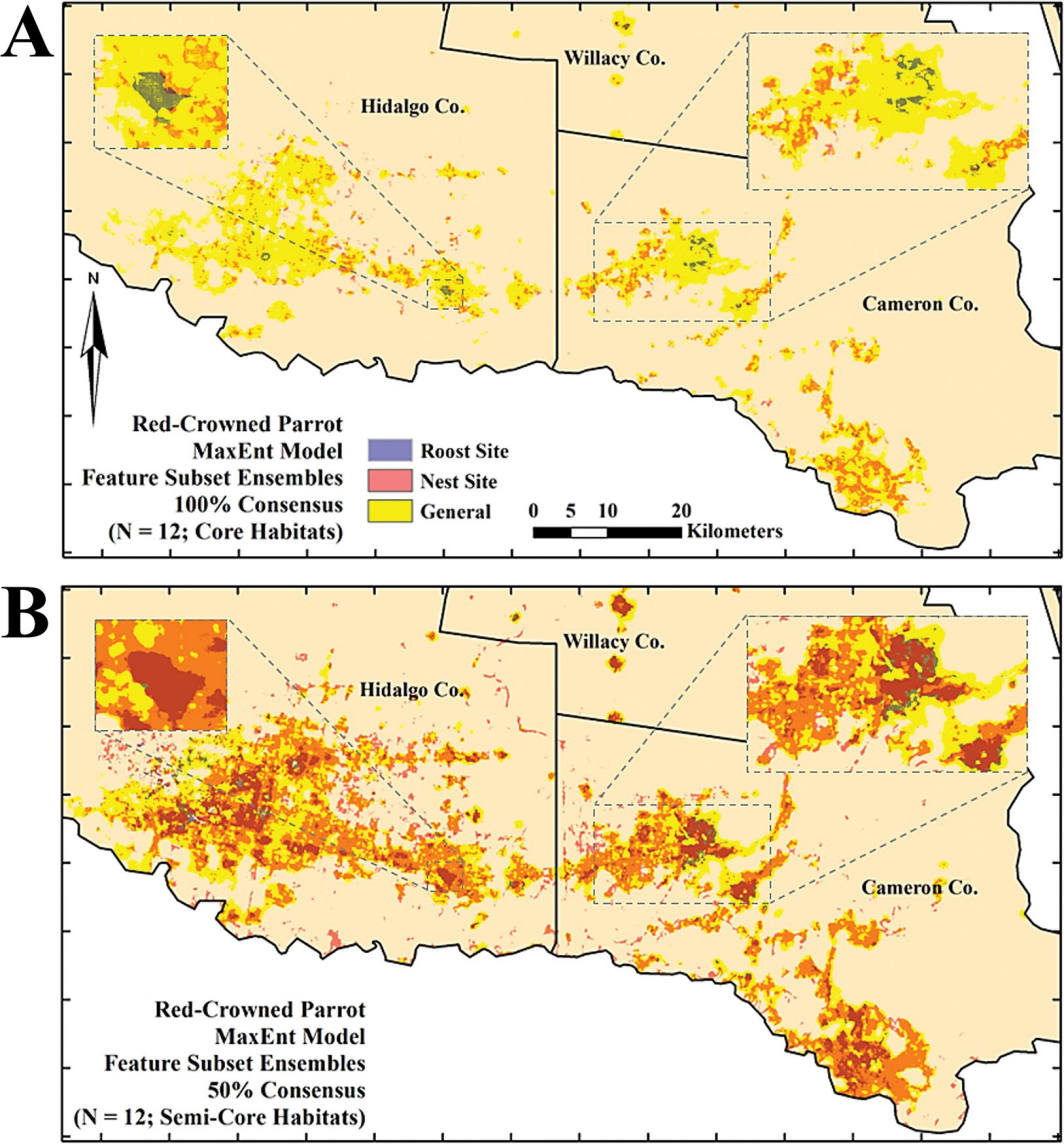
The overlap of Texas Rio Grande Valley Red–crowned Parrot predicted general use, roost site, and nest site habitat distributions as determined using consensus-based feature ensembles of separate sets of 12 calibrated (binary presence/absence) MaxEnt models. (A) 100% consensus (“core”); (B) 50% consensus (“semi–core”). Insets depict certain parts of the predicted habitat distributions in more detail.

Most of the combined areal extents of the core projections of the roost and nest site habitat distributions fell within the bounds of that of the general use habitat distribution (*i*.*e*., 128.1 km^2^ [88.0%]; Fig 10). Most of the areal extent of the semi–core projection of the nest site habitat distribution also fell within the bounds of that of the general use habitat distribution (*i*.*e*., 837.2 km^2^ [82.4%]). The same was true for the areal extent of the semi–core projection of the roost site habitat distribution (*i*.*e*., 296 km^2^ [94.1%]; Fig 10). There was more overlap between the semi–core projections of the roost and nest site habitat distributions but little to no overlap between the core projections. The combined areal extent of the core and semi–core projections of nest site and roost site habitat distributions was 145 km^2^ and 1058.4 km^2^, respectively. Approximately 1.06 km^2^ (0.73%) and 272.9 km^2^ (25.8%) of their areal extents of their core and semi–core projection overlapped, respectively (Fig 10).

## Discussion

### Top–ranked variables in habitat distribution models

#### Topography

*Elevation* was the fifth and six top–ranked variable in the 250 top–performing general use and nest site habitat distribution models, respectively, but not in roost site habitat distribution models. It appeared relatively frequently in the general use and nest site feature subsets (S1, S2 Tables); however, its mean permutation importance never exceeded approximately 50% in any of the 12 top–selected models. The frequent appearance of *elevation* was likely related to the fact that it was the only variable in its correlation group (and thus had a much higher chance of appearing in any of the feature subsets derived by the RSFSA-CV. *Elevation* was a top variable in a model of the distribution of Red–crowned Parrot populations in southern California [34]. Interestingly, although Red–crowned Parrot presence appears to be restricted to elevations between one to 70 m in the Rio Grande Valley of Texas, they occupy elevations upwards of 1,200 m in Mexico [120] and 400 m in California [121]. Given that the majority of the urban areas of the Rio Grande Valley are generally low–lying and unremarkable in terms of topographic variability [86], we suggest that while elevation is not a primary driver of Red–crowned Parrot presence in this area in and of itself, it may be important in that it influences the distribution of vegetation they rely on as it relates to different habitat types. For example, elevation may play a meaningful role in defining differences between roosting and foraging habitats as Red–crowned Parrots reportedly travel up and down elevational gradients between foraging and roosting areas in Mexico [122]. Unfortunately, we were unable to model their foraging habitat distribution in this study due to a lack of a sufficient number of unique foraging presence points.

#### Raw vegetation indices

Raw vegetation indices occasionally appeared in some of our top–performing models and among our list of top–ranked variables (Table 3; S1, S2 Tables), suggesting this feature type had some explanatory value in our final habitat distribution models overall. However, mean permutation importance for this variable type was usually relatively low in all three sets of our habitat distribution models. We were somewhat surprised that raw SARVI did not seem to have more explanatory power in our models than raw NDVI (which had relatively low explanatory power in and of itself). The mediocre performance of raw SARVI could have been caused by the fact that the value we chose for “*L*”, the soil correction factor (*i*.*e*., 0.5), might have been inappropriate for calculating this vegetation index using NAIP imagery because the calculations (and constants) used to derive SARVI were originally developed to use on Moderate Resolution Imaging Spectroradiometer (MODIS; a different satellite image acquisition platform that uses somewhat different ranges of spectral values for similarly named bands) imagery [123]. The additional non–linear transformations made on one version of raw SARVI (*i*.*e*., *sarvi08trans*; S1 Table) to bring out differences between varying pixel values in the original raster seemed to have little effect on its performance in models.

Despite its relatively poor performance in our models, we suggest the utility of raw vegetation indices, including SARVI, in SDMs deserves further research since they have been useful in other studies [60, 124]. This is supported by their utility in other SDMs for birds, including parrots. Raw NDVI was successfully used to determine the extent to which deforestation affected Neotropical parrot population occupancy rates [125]. Mean annual NDVI was the most important variable for modeling the distribution of the IUCN Critically Endangered Lilacine Parrot (*Amazona lilicana* [Lesson, 1844]) in Ecuador [36]. Raw vegetation indices were used to model the habitat distribution of the IUCN Vulnerable Red-fronted Parrotlet (*Touit costaricensis* [Cory, 1913]) in Costa Rica [126]. The spatial distributions of raw NDVI and EVI values associated with Clapper Rail (*Rallus longirostris yumanensis* [Dickey, 1923]) presence was not random [127]. Other vegetation indices that might be valuable for developing SDMs in urban landscapes are those that include the red and blue bands (*e*.*g*., squared Red-Blue NDVI [97]) because they can be used to effectively discriminate between vegetated and non–vegetated surfaces. The utility of very few other types of vegetation indices have been explored in SDMs to date. We strongly encourage thinking outside of the “NDVI” box when choosing which vegetation indices to include as predictor features in future SDMs.

#### Multitemporal raw vegetation indices

The multitemporal raw vegetation index difference type variables were generally poorly ranked in all three habitat distribution models. Only one of these variables had a small, but measurable permutation importance in one of the 12 top–selected models of our three habitat distributions (S1, S2 Tables). We suggest the changes in the landscapes’ vegetation that occurred between April 2008 and April 2010 were either relatively minor or had little ecological significance to the Red–crowned Parrots’ survival. Multitemporal vegetation index difference type features might be more useful in SDMs when a significantly greater or lesser period of time between the dates that the imagery used to create them is acquired has lapsed. For example, since parrots rely on different types and parts of vegetation throughout the year (*e*.*g*., seasonal availability) for various purposes [128], multitemporal vegetation index differences that consider differences between values of vegetation indices over different periods might improve the utility of this type of variable in SDMs for parrots. For example, considering changes in NDVI values between the months of April (spring) and July (summer) or December (winter) within a single year might highlight important differences in vegetation that have a stronger influence on habitat suitability of certain areas during different seasons throughout the year compared to differences between vegetation as it occurs during the same month after a single year has lapsed.

#### Percent cover of preferred ranges of raw vegetation index values

This type of variable appeared slightly more frequently in top–performing models than other types of simple vegetation index derivatives and was often more highly ranked; however, it was never the top–performing variable by mean permutation importance in any of the 250 top–performing models (Table 3; S1, S2 Tables). Percent cover of preferred ranges of raw SARVI and NDVI values appeared to have slightly more explanatory power than that of other vegetation indices we considered (*i*.*e*., BNDVI, GBNDVI, GRNI; S1 Table). The general shape of the response curves for this type showed strong decreases in probability of parrots being low at minimum and maximum values of percent cover and a dramatic increase somewhere in between). This implies that Texas Rio Grande Valley Red–crowned Parrots prefer moderate rather than high or low levels of percent cover of preferred vegetation types or characteristics. The Western Ground Parrot (*Pezoporus flaviventris* [North, 1911]) also prefers habitat with moderate, rather than high or low levels of vegetative cover [129]. While it makes sense that the probability of Red-crowned Parrot presence is minimal at low levels of percent cover of vegetation types or characteristics (since they are so strongly dependent on vegetation as previously mentioned), it was somewhat unclear why the probability of their presence decreases at maximal values. Another parrot species’ habitat use patterns provided us with some insight regarding this matter. Higher vegetative cover was associated with lower productivity at nest sites and higher rates of predation in two separate populations of Green-rumped Parrotlets (*Forpus asserines* [Linnaeus, 1758]), which also occupy vegetatively heterogenous habitats in Venezuela [130]. Although this variable type was typically not relatively well-performing in our models, we suggest it is still worth considering in future SDMs for birds to explore their preferences for vegetative heterogeneity.

#### Raw vegetation index textures

This variable type appeared frequently and was often highly ranked in the 250 top–performing models, including the 12 selected to derive all three habitat distribution projections (Table 3; S1, S2 Tables). Particularly valuable variables of this type included 2^nd^–order GLCM entropy and/or contrast textures of raw NDVI, GBNDVI, and BNDVI values. In addition, 2^nd^–order GLCM entropy textures of raw GRNDI were highly ranked in roost site habitat distribution models. In most cases, the marginal responses of this type of variable showed probability of parrot presence increased as their values increased. The 2^nd^–order GLCM entropy and contrast textures of raw NDVI have been correlated with heterogeneity of certain vegetation pattern characteristics (*e*.*g*., species richness, canopy height, etc.) [131]. As such, the combination of the strong performance of entropy and contrast textures of raw vegetation index–based features in all three sets of habitat distribution models and marginal response curves suggests Red–crowned Parrot presence is tied to high levels of heterogeneity in vegetative characteristics such as canopy structure and/or types of vegetation. This aptly describes the urban areas of the Texas Rio Grande Valley where the Red–crown Parrots can often be found. This assertion is particularly well–supported by our predicted roost site habitat distribution because their roosts in the area of interest have previously been described as a collective of small but well–vegetated suburban yards [13]. Vegetative diversity may be generally important to the persistence Red–crowned Parrot wherever it is found given that diverse deciduous tropical forest is the habitat type it is most commonly associated with in the Mexican portion of its historical range [132]. Vegetative diversity has also been found to be an important factor in defining the habitat distributions of other parrot species [133–135].

Although vegetation index texture type features are not yet commonly employed in SDMs, several studies provide support for the assertion that they are worth including in SDMs in the future. Various textures (*e*.*g*., entropy, contrast, homogeneity, dissimilarity) of raw vegetation indices (*e*.*g*., NDVI, Soil Adjusted Total Vegetation Index [SATVI], Enhanced Vegetation Index [EVI]) outperformed other variables in wide range of models (*i*.*e*., Critically Endangered Alaotran gentle lemur (*Hapalemur alaotrensis* [Rumpler, 1975]) habitat [62], bird species densities and avian species richness [136], avian species diversity in the Chihuahuan Desert [131], vegetation species richness in Argentinian mesquite (*Prosopis alba* [Griseb]) woodlands [137], vegetation canopy height and bird species richness [138], vegetation distribution and heterogeneity, spatial variation of different habitats, and predicting key biodiversity patterns [138, 139]). While we did not explore GLCM 2^nd^–order homogeneity or dissimilarity textures of raw vegetation indices in our models, we suspect that they might have performed a function similar to some of the GLCM 2^nd^–order textures we did explore (*i*.*e*., contrast, entropy) given their usefulness in detecting vegetation heterogeneity [131]. The utility of GLCM textures of vegetation indices should continue to be explored in future SDM efforts.

#### Texture of binary preferred/non–preferred ranges of vegetation index values

In general, this variable type was much lower ranked than other complex vegetation derivatives such as either texture of raw vegetation indices (see above) or percent cover of preferred ranges of vegetation index texture values (see below; Table 3). These variables were higher ranked for general and roost site habitat distribution models compared to nest site habitat models, where only one of these variables appeared in the top 30 ranked variables (Table 3). These variables commonly appeared in the selected 12 top–selected models used to derive the habitat distribution models for each habitat type; however, few had high permutation importance, even in some nest site habitat distribution models. Further study is needed to assess whether these variables would contribute more to models in the absence of higher ranked vegetation index texture–type variables and how overall model performance might be affected.

#### Percent cover of preferred ranges of vegetation index texture values

This variable type had one of the highest ranked performances of all the types; the top–ranked variable of both the top–performing 250 models for the general use and roost site habitat distributions was of this type (Table 3; S1, S2 Tables). Furthermore, they were the variable type most likely to be *the* top–performing variable in roost site habitat distribution models. Those derived using 2^nd^–order GLCM entropy and contrast textures appeared more frequently in models and were often more highly ranked than other texture types we considered. Unlike the response curves for percent cover of preferred ranges of raw vegetation index values, the marginal response curves for this variable type showed that the probability of parrot presence was either moderate and stable (*i*.*e*., mean, variance) or increased from nearly zero to close to maximum (*i*.*e*., contrast, entropy) as percent cover of the preferred range of values increased. This supports the suggestion that heterogeneity in vegetation or its characteristics is an important factor in how these parrots are distributed across the urban Texas Rio Grande Valley landscape. While it is tempting to gain a better understanding of what the landscape actually looks like as values of this variable type changes on the ground, we reiterate that the purpose of using this type of variable in SDMs is to identify mathematical patterns that might be important to the species of interest from their perspective rather than an anthropocentric one [52]. More research is needed to explore the utility of this type of variable in SDMs.

#### Additional notes regarding vegetation index type variables in SDMs

The vegetative cover varies across landscapes in both time and spaces because each plant species has its own unique life cycle and phenology, and as such, the manner in which Red–crowned Parrots (and many other animals) likely exploit whatever suitable vegetation is available in the areas they occupy at any given time of year [140]. As such, it is important to recognize that the utility of raw vegetation indices and their derivatives in our SDMs was limited by what vegetative cover could be detected during the two to three days when the aerial images from which said vegetation indices (and their derivatives) were created were captured [141]. The predictive value of vegetation indices in SDMs is probably highest when associated with presence data collected around the same period. Since the Red–crowned Parrot engages in resting, foraging, nesting, and roosting (much of which is associated with interactions with vegetation) during the time of year that the aerial imagery we used to derive our predictive features was collected, we are confident that our models are valid predictions of the habitat distributions of interest during the spring season; however, their predictive accuracy may not be as strong during other seasons. A time series of aerial (or satellite) imagery can be used to track target plant species in a more continuous manner and thereby improve the capacity to discriminate between different plant species [142]. This concept could be applied to a wide variety of aerial imagery products that could be used in SDMs, including vegetation indices (assuming a time series of the desired imagery is available).

### Focal window size

Variables derived using the larger focal window size (*i*.*e*., 990 m) made up higher percentages of top–ranking variables that made up the 250 top–performing models across all three habitat distributions (S2 Table). This suggests Red-crowned Parrot presence in the Rio Grande Valley of Texas appears to be more strongly correlated with vegetation patterns that occur over larger as opposed to smaller ones. Small patches (*i*.*e*., 10 to 100 m^2^) are likely to provide fewer food resources and potentially less cover from predators for birds that rely on specific habitat (*e*.*g*., woodlands) compared to large patches (*i*.*e*., >100 m^2^) [143]. Occupying areas that provide access to desired resources over relatively larger areas with high levels of heterogeneity in vegetative cover may allow the parrots to be choosier about deciding where to go to acquire what is needed or desired (*i*.*e*., food, mates, places to rest or nest) while simultaneously enabling them to avoid negative outcomes (*i*.*e*., predation) more effectively. Despite this, it is important to consider that any movement across the landscape involves making decisions based on a simple cost/benefit trade–off scheme as it relates energy use and predation risk [144]. As such, there is likely a maximum threshold for the size of the area that could feasibly be covered by the parrots because of the increasing cost of travel required to exploit increasingly dispersed patches with the desired resources and/or that provide effective protection from predators and less competition. This assertion is supported by the finding that large birds must travel farther when they occupy relatively homogenous landscapes [138]. Since the Red-crowned Parrot is considered a relatively large bird [132], we suspect this phenomenon could affect them. They may be able to persist in certain parts of the urban landscape that are fairly heterogeneous (*i*.*e*., a fair number of smaller patches of suitable habitat [*e*.*g*., appropriately vegetated] within reasonable distances of each other).

Red-crowned Parrots are highly mobile, strong fliers [23] that move readily between resource patches used for foraging, roosting, nesting, or other activities. Regular daily movements can exceed 20 km round–trip during the breeding season. They can make larger (~40 km), more irregular movements between roosting and foraging sites during the non–breeding season [23, 145] in addition to a number of shorter movements between daily foraging bouts. The focal window sizes considered in this study may have therefore been too small. We suggest future SDMs considering using predictor features that require neighborhood calculations explore a wider range of focal window sizes.

Variables that did not involve a focal neighborhood analysis were often less highly ranked than those that did; the exception was *elevation*. We suggest this indicates that the distributions of the various resource patches Red-crowned Parrots exploit across Rio Grande Valley are likely more important than the exact location of any single patch in isolation [146].

### LULC composition

The results of the LULC composition analyses confirm that the majority of all three of the Red–crowned Parrots’ habitat suitability distributions of interest are primarily composed of LULC types affected by urbanization (*i*.*e*., *Low Urban*, *High Urban*); however, it is important to recognize that urbanization in and of itself does not equate to Red-crowned Parrot presence because not all areas classified as being affected by urbanization fell within the boundaries of our predicted habitat distributions. Other LULC types that fell within their boundaries (*e*.*g*., grasslands, agriculture areas [*i*.*e*., *Row Crops*, *Orchards*], thornscrub, shrubland, woodlands, wetlands, etc.) were more closely related to those found in their habitat in Mexico and may somehow support the parrots’ persistence in the Texas Rio Grande Valley. The natural habitat of the endangered Red-crowned Parrot in Mexico consists of tropical lowland forests (*e*.*g*., deciduous, gallery, evergreen floodplain), semi–open woodlands (*e*.*g*., open pine–oak savannah), and occasionally open areas (*e*.*g*., coastal plains, cattle pastures) dotted with large trees [16, 17, 23, 147].

While the LULC analysis was of some use in supporting the basic hypotheses that these parrots use certain areas within urban landscape the Rio Grande Valley of Texas, we stress that it did not contribute much novel information to our understanding of the habitat distributions of the Red–crowned Parrot beyond this. Since LULC type maps are typically developed for certain types of human use at scales of interest to humans [148], they may not be as useful for developing SDMs of non–human animals species as currently perceived.

### Suitable habitat distribution overlap

The finding that the predicted projections of roost and nest site habitat distributions fell within the bounds of those of the general use habitat distribution was expected given that at least a small number of the presence points used to model the general use habitat distribution were undoubtedly representative of presence at nest or roost sites. The predicted nest and roost site distributions may overlap since they are both almost exclusively found in suburban yards throughout the study site. Additionally, Red–crowned Parrots are secondary tree cavity nesters (*i*.*e*., they rely on holes excavated by other species [122]) and typically roost in trees like most other parrots [31, 149–151] and it is likely that there are at least some trees suitable for either nesting or roosting within the area that comprises the general use habitat distribution. We suspect that areas where no overlap occurs between the general use habitat distribution and either of the other two habitat distributions could be areas used by the parrots use for activities such as foraging, resting, and/or predator evasion.

### Conservation implications and recommendations

An increasing number of parrot species, including those of conservation concern, can be found in cities around the world and it has been suggested that cities may be a novel niche parrots are starting to exploit more and more frequently [15, 152–154]. As such, the Texas Rio Grande Valley Red-crowned Parrot is far from the only sensitive parrot species to find a home in urbanized landscapes. For example, the International Union for the Conservation of Nature (IUCN) Vulnerable Hispaniolan Parrot (*Amazona ventralis* [Muller, 1776]) and Hispaniolan Parakeet (*Psittacara chloropterus* [de Souancé, 1856]) as well as the Vulnerable Forest Red-tailed Black Cockatoo (*Calyptorhynchus banksia naso* [Gould, 1837]) and the Critically Endangered Yellow–crested Cockatoo (*Cacatua sulphurea* [Gmelin, 1788]) are found in cities in the Dominican Republic, Western Australia, and Hong Kong, respectively [155–157]. Regardless of how or why parrots have been able to find a home in various cities around the world, it is far from certain that they will be able to persist in them in the future. Urban landscapes are associated with high levels of human activity, and as a result, often change and/or expand so rapidly that it can difficult for even the most hardy, well–established plants and animals to adapt quickly enough for them persist without some sort of help or protection from humans [158, 159]. The cities found in the Texas Rio Grande Valley are no exception to this phenomenon. The regions’ landscapes are becoming increasingly urbanized at an exceptional rate and there is no sign that this trend will change any time soon. The human population is estimated to double from 1.3 to 2.4 million by 2045 [160]. We suspect the area will likely grow even more rapidly than predicted and change in a dramatically different way than expected with the arrival of SpaceX’s headquarters to the Brownsville in 2014 [161]. Additionally, the launch of SpaceX rockets in the area will also likely impact the way animals such as birds are distributed and move around the area. Indeed, a static firing of its Super Heavy rocket booster in early 2023 “sent up a massive plume of smoke and dust as birds scattered around in the launch site” [162].

Our results suggest that vegetation heterogeneity is an important factor in the way Red–crowned Parrots are distributed across certain parts of the urban areas of the Texas Rio Grande Valley. An adequate network of green spaces, including appropriately vegetated yards of homes and businesses, where a diversity of vegetation can be found within the greater urban matrix in this region will likely be essential to the persistence of the existing Red–crowned Parrot populations found there in the future; such spaces likely also benefit numerous other animals, including other birds of conservation concern [163–165] Although some bird species require a network of fairly large patches of habitat undisturbed by humans in order to survive in urban areas [159, 166–168], the Red-crowned Parrot appears capable of utilizing a network of relatively small but abundant patches of green spaces with the right characteristics. The maintenance of remnants of native vegetation is essential for drawing and keeping native bird assemblages to urban areas [169]. Additionally, given that small portions of two of the three distributions of interest (*i*.*e*., general use and nest site habitat) were classified as agricultural land, grassland and/or a few other natural LULC types, small patches of these LULC types should be preserved within the urban matrix to increase the heterogeneity in vegetative cover across the greater landscape.

Well–designed green spaces in urban areas are beneficial to human health (*i*.*e*., provide ecosystem services, increase property values, improve physical and mental well–being, etc.) [170, 171]. In addition to maintaining the existing diversity of vegetation wherever the parrots already occur (especially those used to forage, nest, and roost), incorporating such vegetation into areas where the parrots are not currently found but desired should create new habitat for them and thus should allow existing populations to grow and/or expand [32, 172]. Areas where new development is occurring should plan to incorporate bird–friendly vegetation in their landscape architecture plans. Plant species commonly used for nesting, foraging, and roosting include: *Washingtonia* spp. palm, eucalyptus (*Eucalyptus* spp. [L’Hér]), mesquite (*Prosopis* spp.), fig (*Ficus* spp.), coma (*Bumelia laetevirens* [Hems]), Texas ebony (*Ebenopsis ebano* [Berland]), silver maple (*Acer saccharinum*), sycamore (*Platanus* spp.), and pecan (*Carya illinoinensis* [Wangenh]). A more expansive list of plant species consumed or used for nests by Red–crowned Parrots can be found elsewhere [10, 13, 23, 173].

Although the pressures on the populations of this species that are found in the Mexican portion of its range have been drastically reduced in recent years [174] thanks to extensive conservation efforts, we expect the range of the Red-crowned Parrot will continue to slowly creep northward because of the effects of climate change as has been observed in many other non-migratory avian species [175]. Since many bird species are reportedly not moving northward at the pace set by the effects of climate change [176], we suspect the Red-crowned Parrot populations that are currently well-established in the Rio Grande Valley of Texas, the northernmost aspect of their range, will only become more valuable to the species as a whole over time. We believe that protecting the Texas Red–crowned Parrots will require a commitment by a combination of interested parties (*e*.*g*., local residents, businesses, and governmental and non–governmental agencies) to develop and carry out a conscientious vegetation management plan (*i*.*e*., support heterogeneity in vegetation type and structure in area; see “Animal–Aided Design” model proposed by Weisser and Hauck [177]) that involves conserving existing green spaces in the region’s urban and suburban areas; new plans should be incorporated into new developments and renovations. These relatively simple actions should help these populations both endure and potentially even grow in size as the landscape changes in the near future [178, 179]. Failure to conscientiously plan for a future with Red–crowned Parrots could result in the decline or even collapse of existing populations.

### Study limitations

This study was limited by several factors. First, sample size would ideally be larger, especially regarding the number of general use and nest site habitat presence points. Nests are inevitably difficult to locate and undoubtedly some were missed despite the monumental efforts undertaken by S.K., D.J.B., and other members of the Tejano Parrot Project to locate as many as possible. Several additional vegetation indices (and likely a nearly unlimited number of derivatives) could have been used and/or created and explored; however, we had to keep a realistic time period in mind. Second, although newer NAIP imagery was available that we would have liked to use, we could not because the files were too large for our readily available computing resources to handle. Third, the feature selection methodology produces a set of 12 models that are randomly selected from a set of 250 top–performing models. Consequently, the 12 top–selected models used to create our final feature subset ensemble from which our final projected habitat distributions were derived may have been slightly different if different sets of top–performing models were selected for their derivation.

## Conclusions

We utilized MaxEnt with feature selection of a list of variables including elevation, vegetation indices and their simple and complex derivatives to create accurate, fine–scale general use, nest site, and roost site habitat distribution models for Texas Rio Grande Valley Red–crowned Parrots. This study revealed the utility of a series of novel variable types as well as variable types that are seldom used in SDMs for birds. Variables such as 2^nd^–order GLCM entropy and contrast textures of raw NDVI and percent cover of preferred ranges of 2^nd^–order GLCM textures of raw vegetation index values performed especially well in our models. The utility of novel types of variables that performed well in our models, as well as those variables that performed well but have previously been rarely used in SDMs efforts, should be further evaluated. We suggest that some of these variables may prove to be of greater value in SDM efforts that aim to use variables derived from higher–resolution aerial imagery (*i*.*e*., ≤10 m resolution; *c*.*f*., ≥30 m resolution imagery may not be as valuable).

Cities have often described as “biological deserts” [180]; however, a number of plants and animals (including those of conservation concern) have successfully adapted to the city life. While cities will never support all of the plants and animals that occupied the landscapes wherever cities are found before they were built [155], we might be able to support the development of novel ecosystems in cities that we can appreciate and that can support a number of “desirable” species by approaching the management of urban landscapes from a nature–positive perspective [181]. We found urbanization does not in and of itself equate to Red-crowned Parrot presence in the Texas Rio Grande Valley, but rather it is only those parts of the region’s cities that have a diverse array of vegetation (and/or certain defining characteristics) that have a relatively high likelihood of being associated with supporting their presence. The urban landscapes of the Texas Rio Grande Valley are changing and expanding so rapidly that it may be difficult for locally occurring flora and fauna to adapt and persist in them in the near future. The recent arrival of SpaceX’s headquarters is likely to magnify this issue. We suggest conservation–oriented management of the region’s urban landscapes may be critical for the continued survival of various bird species, including the Red-crowned Parrot, in the Rio Grande Valley of Texas. We recommend that parties interested in supporting the persistence of these parrots: (1) manage existing parrot–friendly vegetation found in urban areas in ways that preserve and otherwise protect it and (2) incorporate new parrot–friendly vegetation into parts of the landscape where existing developments occur where the parrots are not currently found and/or new developments as they are being planned.

Given the aforementioned threats the species continues to face in Mexico, the Texas Rio Grande Valley Red-crowned Parrots may very well end up being a critical genetic reservoir that ultimately ends up being vital to the long–term survival of the species [182, 183]. We argue they should be treated as the valuable resource they are today and could be in the future. The USA’s approach to how they choose to treat these parrots should therefore be one that focuses on protection and conservation. Parrots do not recognize political borders and as such, we suggest that parties from both the USA and Mexico must come together to effectively conserve the Red–crowned Parrot populations found in the Rio Grande Valley in order for them to persist for generations to come.

## Supporting information

S1 File Supplementary materials. This file contains the supplementary materials associated with the manuscript entitled “Modelling Red-crowned Parrot (Psittaciformes: *Amazona viridigenalis* [Cassin, 1853]) distributions in the Rio Grande Valley of Texas using elevation and vegetation indices and their derivatives”.
(DOCX)

S1 Fig A digital elevation model used to represent topography. This variable, *elevation*, was derived using parts of the 10 m United States Geological National Elevation Survey dataset.
(TIF)

S2 Fig An example of a type of a raw vegetation index type feature. The variable raw 2008 Blue Normalized Difference Vegetation Index (BNDVI), *bndvi08*, was created by performing a simple calculation to the information contained in each of the three spectral bands of National Aerial Imagery Program (NAIP) images for 2008 (Table 1).
(TIF)

S3 Fig An example of a type of multitemporal raw vegetation index difference type feature. This variable, *deltgrn10_08*, was derived by subtracting raw 2008 Green Red Normalized Difference Index (GRNDI; *grndi08*) from raw 2010 GRNDI (*grndi10*).
(TIF)

S4 Fig Frequency of Red-Crowned Parrot occurrences (n = 1,847 held out from niche modelling) in the Texas Rio Grande Valley associated with raw 2010 Normalized Difference Vegetation Index (NDVI) values. Blue dashed lines represent the central range (*i*.*e*., 50%) of raw 2010 Normalized Difference Vegetation Index (NDVI) values (*i*.*e*., -211 to 156) while red dotted lines represent the central range (*i*.*e*., 70%) of raw 2010 NDVI values (*i*.*e*., 191 to 155) of parrot presence points around the median raw 2010 NDVI value of 21.
(TIF)

S5 Fig An example of a type of a percent cover of preferred ranges of raw vegetation index values type feature. This variable, *bndvi_70_990*, depicts the percent cover of a preferred range of raw 2008 Blue Normalized Difference Vegetation Index (BNDVI; *bndvi08*) values (*i*.*e*., set of values associated with Red-crowned Parrot presence points about the median value). First, we calculated the preferred range of *bndvi08* values (70%; see S4 Fig), which was then used to create a binary preferred/non-preferred raster. The final raster was created by applying a focal window analysis to the binary raster using a square 990 m focal window.
(TIF)

S6 Fig An example of a type of raw vegetation index texture type feature. This variable, *bnd082c990*, represents the 2^nd^-order Gray Level Co-Occurrence Matrix (GLCM) contrast texture of raw 2008 Blue Normalized Difference Vegetation Index (BNDVI; *bndvi08*) values that was derived using a square 990 m focal window.
(TIF)

S7 Fig An example of a type of texture of binary preferred/non-preferred ranges of raw vegetation index values type feature. This variable, *bnd08m70310*, depicts the 2^nd^-order Grey Level Co-Occurrence Matrix (GLCM) mean texture of the binary preferred/nonpreferred range of raw 2008 Blue Normalized Difference Vegetation Index (BNDVI) values (*i*.*e*., central set of values associated with Red-crowned Parrot presence points about the median value). Deriving this variable involved (1) calculating the central range of raw 2008 BNDVI values (*i*.*e*., 70%; see S4 Fig), (2) creating a binary preferred/nonpreferred raster using the aforementioned ranges, and (3) calculating the 2^nd^-order GLCM mean texture for the binary raster using a square 990 m focal window.
(TIF)

S8 Fig An example of a type of percent cover of preferred ranges of vegetation index texture values type feature. This variable, *bnd082c99pc85*, depicts the percent cover of preferred ranges of 2^nd^-order Grey Level Co-Occurrence Matrix (GLCM) contrast texture of raw 2008 Blue Normalized Difference Vegetation Index (BNDVI) values (*i*.*e*., set of values associated with Red-crowned Parrot presence points about the median value). Creating this variable involved (1) calculating the central range (*i*.*e*., 85%) of 2^nd^-order GLCM contrast texture of raw 2008 BNDVI values derived using a square 990 m focal window, (2) creating a binary preferred/non-preferred raster from this range, and (3) calculating the percent cover of preferred areas from the binary raster using a square 990 m focal window.
(TIF)

S9 Fig Texas Rio Grande Valley Red-crowned Parrot general use habitat distribution jackknife analysis of Area under the Curve (AUC) accuracy values for individual features and response curves for individual features of the 12 top-selected MaxEnt models. The jackknife of AUC for species graph (left) and response curve (right) results for each of the final models used to create the feature subset ensemble for the suitable general use habitat distribution.
(TIF)

S10 Fig Texas Rio Grande Valley Red-crowned Parrot nest site habitat distribution jackknife analysis of Area under the Curve (AUC) accuracy values for individual features and response curves for individual features of the 12 top-selected MaxEnt models. The jackknife of AUC for species graph (left) and response curve (right) results for each of the final models used to create the feature subset ensemble for the suitable nest site habitat distribution.
(TIF)

S11 Fig Texas Rio Grande Valley Red-crowned Parrot roost site habitat distribution jackknife analysis of Area under the Curve (AUC) accuracy values for individual features and response curves for individual features of the 12 top-selected MaxEnt models. The jackknife of AUC for species (left) and response curve (right) results for each of the final models used to create the feature subset ensemble for the suitable roost site habitat distribution.
(TIF)

S1 TableVariable performance in 250 top-performing models and the subset of 12 models selected from these used to create the final projections for the predicted habitat distributions of Texas Rio Grande Valley Red-crowned Parrots.$\bar{x}$, arithmetic mean; *s*, standard deviation of the mean; Transf, transformation; Corr Group, correlation group; Imprt, importance; GLCM, Grey Level Co-occurrence Matrix; m, meters; b/w, between; diff, difference. For Corr Group: G, General Use; N, Nest Site; R, Roost Site. For Variable Rank, bolding indicates that the feature of interest appeared in Table 3 as one of the 30 top-ranked variables for its respective set of habitat distribution models. *Variable only appeared in one of three replicates of the 250 top-performing models for a given habitat distribution. **Variable only appeared in two of the three replicates of the 250 top-performing models for a given habitat distribution. Variables that belonged to the same correlation group did not appear in models together. Our correlation cutoff was |0.5|, which is more restrictive than previous instances where this version of RSFSA-CV has been used.(XLSX)

S2 Table Occurrence of variable types that appeared in the 250 top-performing models and subset of 12 selected to derive projections of predicted Texas Rio Grande Valley Red-crowned Parrot general use, nest site, and roost site habitat distributions. Includes the percentage of selected 12 top-selected models with at least one occurrence of variable type [% Models] and percentage among all 250 top-performing models with variable type being the top-performing variable by permutation importance (% Top). Percentage of variables used in top-selected 12 models does not include variables with 0.0% mean permutation importance (omitted from final models).
(XLSX)

## References

[1] Houghton RA. The worldwide extent of land-use change. Biosci. 1994;44(5):305–13. Available from: 10.2307/1312380.

[2] Lindenmayer DB, Fischer J. Habitat Fragmentation and Landscape Change: An Ecological and Conservation Synthesis. Washington, D.C.: Island Press; 2013.

[3] Gomes V, Ribeiro R, Carretero MA. Effects of urban habitat fragmentation on common small mammals: species versus communities. Biodivers Conserv. 2011;20(14):3577–90. Available from: 10.1007/s10531-011-0149-2.

[4] Wanger TC, Iskandar DT, Motzke I, Brook BW, Sodhi NS, Clough Y, et al. Effects of land‐use change on community composition of tropical amphibians and reptiles in Sulawesi, Indonesia. Conserv Biol. 2010;24(3):795–802. Available from: 10.1111/j.1523-1739.2009.01434.x. 20151989

[5] Alberti M. The effects of urban patterns on ecosystem function. Int Reg Sci Rev. 2005;28(2):168–92. Available from: 10.1177/0160017605275160.

[6] Di Giulio M, Holderegger R, Tobias S. Effects of habitat and landscape fragmentation on humans and biodiversity in densely populated landscapes. J Environ Manage. 2009;90(10):2959–68. Available from: doi: 10.1016/j.jenvman.2009.05.002 19493609

[7] McKinney ML. Effects of urbanization on species richness: a review of plants and animals. Urban Ecosyst. 2008;11(2):161–76. Available from: 10.1007/s11252-007-0045-4.

[8] Blair RB. Land use and avian species diversity along an urban gradient. Ecol Appl. 1996;6(2):506–19. Available from: 10.2307/2269387.

[9] Faeth SH, Bang C, Saari S. Urban biodiversity: patterns and mechanisms. Ann NY Acad Sci. 2011;1223(1):69–81. Available from: doi: 10.1111/j.1749-6632.2010.05925.x 21449966

[10] Froke JB. Populations, Movements, Foraging and Nesting of Feral *Amazona* Parrots in Southern California. Doctoral Dissertation. Humboldt State University; 1981.

[11] Hall LA. Habitat Variables which Influence the Dissemination and Colonization of Introduced Psittacines in Southern California. Master’s Thesis. San Diego State University; 1988.

[12] United States Fish and Wildlife Service. Endangered and Threatened Wildlife and Plants: Red-Crowned Parrot. In: Department of the Interior, editor. Washington, D.C.: Federal Register 2011; 76(194):62016–34. Available from: https://www.fws.gov/policy/library/2011/2011-25808.html.

[13] Kiacz S, Shackelford CE, Henehan AK, Brightsmith DJ. History, status, and productivity of the Red-crowned Amazon (*Amazona viridigenalis*) in the Lower Rio Grande Valley of Texas. Bird Conserv Intl. 2021:1–15. Available from: 10.1017/S0959270920000404.

[14] Red-crowned Amazon [Internet]. International Union for Conservation of Nature and Natural Resources (IUCN); [last updated 2019; cited 2021 July 28]. Available from: https://www.iucnredlist.org/species/22686259/152441187.

[15] Pruett-Jones S, editor. Naturalized Parrots of the World: Distribution, Ecology, and Impacts of the World’s Most Colorful Colonizers. Princeton, NJ: Princeton University Press; 2021.

[16] Clinton-Eitniear J. Status of the Green-cheeked Amazon in northeastern Mexico. Watchbird. 1986;12(6):22–5.

[17] Clinton-Eitniear J. Green-cheeked Amazon update. Watchbird. 1988;15(3):28–9.

[18] Neck RW. Expansion of Red-crowned Parrot, *Amazona viridigenalis*, into southern Texas and changes in agricultural practices in northern Mexico. Texas Ornithological Society. 1986;19:6–12.

[19] Ridgely RS. The current distribution and status of mainland Neotropical Parrots. In: Pasquier RF, editor. Conservation of New World Parrots. Washington, D.C.: Smithsonian Institution Press; 1980.

[20] United States Fish and Wildlife Service. Endangered and Threatened Wildlife and Plants; 12-Month Findings on Petitions to List Eight Species as Endangered or Threatened Species. In: Department of the Interior, editor. Washington, D.C.: Federal Register 2019; 84(5):13237–42.

[21] Monterrubio-Rico TC, Charre-Medellín JF, Pacheco-Figueroa C, Arriaga-Weiss S, de Dios Valdez-Leal J, Cancino-Murillo R, et al. Distribución potencial histórica y contemporánea de la familia Psittacidae en México. Rev Mex Biodivers. 2016;87(3):1103–17. Available from: 10.1016/j.rmb.2016.06.004.

[22] Sutton GM, Burleigh TD. A list of birds observed on the 1938 Semple Expedition to northeastern Mexico. Occ Pap Mus Zoo. 1939;1(3):15–36. Available from: 10.31390/opmns.003.

[23] Enkerlin-Hoeflich EC, Hogan KM. Red-crowned Parrot, *Amazona viridigenalis* In: Poole A, Stettenheim P, Gill FB, editors. Birds of North America. Philadelphia, PA: American Ornithologists’ Union and Academy of Natural Sciences of Philadelphia; 1997.

[24] Collar NJ. Order Psittaciformes, Family Psittacidae (Parrots). In: del Hoyo J, Elliot A, Sargatal J, editors. Handbook of the Birds of the World. 4: Sandgrouse to Cuckoos. Barcelona, Spain: Lynx Edicions; 1997.

[25] Macías-Caballero C, Enkerlin-Hoeflich EC. Evaluación del estado poblacional actual de dos especies Mexicanas de loro en peligro de extinción parte II–loro tamaulipeco (*Amazona viridigenalis*). Monterrey, Nuevo León, Mexico; 2003.

[26] Howell SNG, Webb S. A Guide to the Birds of Mexico and Northern Central America. Oxford, U.K.: Oxford University Press; 1995.

[27] Voltura EV, Brightsmith DJ, Cornejo J, Tizard I, Bailey CA, Heatley JJ. Parrot dietary habits and consumption of alternate foodstuffs: a literature review. J Avian Med Surg. 2022;In Press:N/A. 10.1647/20-00028 38363162

[28] Thick-billed Parrot [Internet]. International: National Park Service; [last updated 2018; cited 2023 May 30]. Available from: https://www.nps.gov/chir/thick-billed-parrot.htm.

[29] Rycken SJE. Movement Ecology of the Three Species of Threatened Black Cockatoo (*Calyptorhynchus latirostris*, *Calyptorhynchus baudinii*, *Calyptorhynchus banksii naso*) Endemic to Western Australia: Implications for the Species’ Conservation Management. Doctoral Dissertation. Murdoch University; 2019.

[30] Garcia D. Birds in ecological networks: insights from bird-plant mutualistic interactions. Ardeola. 2016;63(1):151–80. Available from: doi: 10.13157/arla.63.1.2016.rp7

[31] Renton K, Salinas-Melgoza A, De Labra-Hernández MÁ, de la Parra-Martínez SM. Resource requirements of parrots: nest site selectivity and dietary plasticity of Psittaciformes. J Ornithol. 2015;156(1):73–90. Available from: 10.1007/s10336-015-1255-9.

[32] Idilfitri S, Mohamad NHN. Role of ornamental vegetation for birds’ habitats in urban parks: case study FRIM, Malaysia. Proc Soc Behav Sci. 2012;68:894–909. Available from: 10.1016/j.sbspro.2012.12.275.

[33] Gray ER. Seasonal Use of Urban Forest Vegetation by Bush Birds. Master’s Thesis. University of Otago; 2014.

[34] Meseck KA. Habitat Distribution for Non-native *Amazona viridigenalis* within San Diego County using Maxent Predictive Model. Master’s Thesis. San Diego State University; 2013.

[35] Winchell CS, Doherty PF Jr. Restoring habitat for coastal California Gnatcatchers (*Polioptila californica californica*). Condor. 2018;120(3):581–95. Available from: 10.1650/CONDOR-17-221.1.

[36] Biddle R, Solis-Ponce I, Jones M, Marsden S, Pilgrim M, Devenish C. The value of local community knowledge in species distribution modelling for a threatened Neotropical parrot. Biodivers Conserv. 2021;30(6):1803–23. Available from: 10.1007/s10531-021-02169-9.

[37] Plasencia–Vázquez AH, Escalona–Segura G, Esparza–Olguín LG. Interaction of landscape variables on the potential geographical distribution of parrots in the Yucatan Peninsula, Mexico. Anim Biodiv Conserv. 2014;37(2):191–203. Available from: 10.32800/abc.2014.37.0191.

[38] Pearson RG. Species’ distribution modeling for conservation educators and practitioners. Less Conservat. 2007;3:54–89.

[39] Bradie J, Leung B. A quantitative synthesis of the importance of variables used in MaxEnt species distribution models. J Biogeogr. 2017;44(6):1344–61. Available from: 10.1111/jbi.12894.

[40] Bianchi TS. The evolution of biogeochemistry: revisited. Biogeochem. 2021;154:141–81.

[41] Stephenson NL. Climatic control of vegetation distribution: the role of the water balance. Amer Nat. 1990;135(5):649–70. Available from: 10.1086/285067.

[42] Florinsky IV, Kuryakova GA. Influence of topography on some vegetation cover properties. Catena. 1996;27(2):123–41. Available from: 10.1016/0341-8162(96)00005-7.

[43] Solon J, Degórski M, Roo-Zielińska E. Vegetation response to a topographical-soil gradient. Catena. 2007;71(2):309–20. Available from: doi: 10.1016/j.catena.2007.01.006

[44] Running SW, Loveland TR, Pierce LL, Nemani RR, Hunt ER Jr. A remote sensing based vegetation classification logic for global land cover analysis. Remote Sens Environ. 1995;51(1):39–48. Available from: 10.1016/0034-4257(94)00063-S.

[45] Comer PJ, Hak JC, Dockter D, Smith J. Integration of vegetation classification with land cover mapping: lessons from regional mapping efforts in the Americas. Veg Class Surv. 2022;3:29–43. Available from: doi: 10.3897/VCS.67537

[46] Young NE, Anderson RS, Chignell SM, Vorster AG, Lawrence R, Evangelista PH. A survival guide to Landsat preprocessing. Ecol. 2017;98(4):920–32. Available from: doi: 10.1002/ecy.1730 28072449

[47] Goslee SC. Analyzing remote sensing data in R: the landsat package. J Stat Softw. 2011;43:1–25. Available from: 10.18637/jss.v043.i04.

[48] Hubert-Moy L, Cotonnec A, Le Du L, Chardin A, Pérez P. A comparison of parametric classification procedures of remotely sensed data applied on different landscape units. Remote Sens Environ. 2001;75(2):174–87. Available from: 10.1016/S0034-4257(00)00165-6.

[49] Young NE, Anderson RS, Chignell SM, Vorster AG, Lawrence R, Evangelista PH. Erratum in. Ecol. 2021;102(11):e03508. Available from: 10.1002/ecy.3508.

[50] Briem GJ, Benediktsson JA, Sveinsson JR. Multiple classifiers applied to multisource remote sensing data. IEEE Transactions on Geoscience and Remote Sensing. 2002;40(10):2291–9. Available from: 10.1109/TGRS.2002.802476.

[51] Cánibe M, Titeux N, Domínguez J, Regos A. Assessing the uncertainty arising from standard land‐cover mapping procedures when modelling species distributions. Divers Distrib. 2022;28(4):636–48. Available from: 10.1111/ddi.13456.

[52] Coulson RN, Tchakerian MD. Basic Landscape Ecology. College Station, TX: KEL Partners; 2010.

[53] Benson KLP, Arnold KA. Red-crowned Parrot [Internet]. Texas A&M University System; [last updated 2020; cited 2020 Feb 23]. Available from: https://txtbba.tamu.edu/species-accounts/red-crowned-parrot/.

[54] Edwards EP. A Field Guide to the Birds of Mexico and Adjacent Areas: Belize, Guatemala, and El Salvador. Austin, TX: University of Texas; 1998.

[55] He KS, Bradley BA, Cord AF, Rocchini D, Tuanmu MN, Schmidtlein S, et al. Will remote sensing shape the next generation of species distribution models? Remote Sens Ecol Conserv. 2015;1(1):4–18. Available from: 10.1002/rse2.7.

[56] Bannari A, Morin D, Bonn F, Huete AR. A review of vegetation indices. Remote Sens Rev. 1995;13(1–2):95–120. Available from: 10.1080/02757259509532298.

[57] Silleos NG, Alexandridis TK, Gitas IZ, Perakis K. Vegetation indices: advances made in biomass estimation and vegetation monitoring in the last 30 years. Geocarto Int. 2006;21(4):21–8. Available from: 10.1080/10106040608542399.

[58] Kalaitzidis C, Heinzel V, Zianis D, editors. A review of multispectral vegetation indices for biomass estimation. Proc of the 29th Symposium of the European Association of Remote Sensing Laboratories; Chania, Greece: 2010.

[59] Xue J, Su B. Significant remote sensing vegetation indices: a review of developments and applications. J Sensors. 2017;2017(1353691):1–17. Available from: 10.1155/2017/1353691.

[60] Pettorelli N, Ryan S, Mueller T, Bunnefeld N, Jędrzejewska B, Lima M, et al. The normalized difference vegetation index (NDVI): unforeseen successes in animal ecology. Clim Res. 2011;46(1):15–27. Available from: 10.3354/cr00936.

[61] Shirley SM, Yang Z, Hutchinson RA, Alexander JD, McGarigal K, Betts MG. Species distribution modelling for the people: unclassified Landsat TM imagery predicts bird occurrence at fine resolutions. Divers Distrib. 2013;19(7):855–66. Available from: 10.1111/ddi.12093.

[62] Lahoz‐Monfort JJ, Guillera‐Arroita G, Milner‐Gulland EJ, Young RP, Nicholson E. Satellite imagery as a single source of predictor variables for habitat suitability modelling: how Landsat can inform the conservation of a critically endangered lemur. J Appl Ecol. 2010;47(5):1094–102. Available from: 10.1111/j.1365-2664.2010.01854.x.

[63] Soto GE, Pérez-Hernández CG, Hahn IJ, Rodewald AD, Vergara PM. Tree senescence as a direct measure of habitat quality: linking red-edge vegetation indices to space use by Magellanic Woodpeckers. Remote Sens Environ. 2017;193:1–10. Available from: 10.1016/j.rse.2017.02.018.

[64] Habel JC, Teucher M, Rödder D, Bleicher MT, Dieckow C, Wiese A, et al. Kenyan endemic bird species at home in a novel ecosystem. Ecol Evol. 2016;6(8):2494–505. Available from: 10.1002/ece3.2038. 27066236 PMC4797158

[65] Annorbah NND, Collar NJ, Marsden SJ. Trade and habitat change virtually eliminate the Grey Parrot *Psittacus erithacus* from Ghana. Ibis. 2016;158(1):82–91. Available from: 10.1111/ibi.12332.

[66] Cody ML. Habitat selection in birds: the roles of vegetation structure, competitors, and productivity. Biosci. 1981;31(2):107–13. Available from: 10.2307/1308252.

[67] Bersier L-F, Meyer DR. Bird assemblages in mosaic forests: the relative importance of vegetation structure and floristic composition along the successional gradient. Acta Oecol. 1994;15(5):561–76.

[68] Villard MA, Trzcinski MK, Merriam G. Fragmentation effects on forest birds: relative influence of woodland cover and configuration on landscape occupancy. Conserv Biol. 1999;13(4):774–83. Available from: 10.1046/j.1523-1739.1999.98059.x.

[69] Tracy JL, Trabucco A, Lawing AM, Giermakowski JT, Tchakerian M, Drus GM, et al. Random subset feature selection for ecological niche models of wildfire activity in western North America. Ecol Modell. 2018;383:52–68. Available from: 10.1016/j.ecolmodel.2018.05.019.

[70] Elith J, Kearney M, Phillips S. The art of modelling range‐shifting species. Methods Ecol Evol. 2010;1(4):330–42. Available from: 10.1111/j.2041-210X.2010.00036.x.

[71] Merow C, Smith MJ, Silander JA Jr. A practical guide to MaxEnt for modeling species’ distributions: what it does, and why inputs and settings matter. Ecography. 2013;36(10):1058–69. Available from: 10.1111/j.1600-0587.2013.07872.x.

[72] Elith J, Phillips SJ, Hastie T, Dudík M, Chee YE, Yates CJ. A statistical explanation of MaxEnt for ecologists. Divers Distrib. 2011;17(1):43–57. Available from: 10.1111/j.1472-4642.2010.00725.x.

[73] eBird occurence download for Red-crowned Parrot [Online Database]. Ithaca, NY: Cornell University; 2020 [cited 2020 Mar 9]. Available from: http://www.ebird.org.

[74] Brightsmith D. Welcome! [Internet]. International: Facebook; [last updated 2022; cited 2021 Feb 3]. Available from: https://www.facebook.com/TejanoParrots/.

[75] Occurrence Download for Red-crowned Parrot [Online Database]. Global Biodiversity Index Facility (GBIF); 2020 [cited 2020 Apr 15]. Available from: 10.15468/dl.xqrtkj.

[76] eBird. Guide to eBird Protocols [Internet]. Ithaca, NY: Cornell University; [last updated 2020; cited 2020 Apr 15]. Available from: https://support.ebird.org/en/support/solutions/articles/48000950859.

[77] Boria RA, Olson LE, Goodman SM, Anderson RP. Spatial filtering to reduce sampling bias can improve the performance of ecological niche models. Ecol Modell. 2014;275:73–7. Available from: doi: 10.1016/j.ecolmodel.2013.12.012

[78] ESRI. ArcGIS Advanced Desktop v. 10.32020. Redlands, CA: Environmental Systems Research Institute; 2020.

[79] eBird [Internet]. Ithaca, NY: Cornell University; [last updated 2023; cited 2022 May 15]. Available from: https://ebird.org/home.

[80] iNaturalist [Internet]. California Academy of Sciences and National Geographic Society; [last updated 2023; cited 2020 Mar 15]. Available from: https://www.inaturalist.org/.

[81] Phillips SJ. A brief tutorial on Maxent. 2017. Available from: https://biodiversityinformatics.amnh.org/open_source/maxent/Maxent_tutorial2017.pdf.

[82] R Core Team. R Studio v. 3.4.3. Vienna, Austria: Posit, PBC; 2021.

[83] Eisenlohr PV, Alves LF, Bernacci LC, Padgurschi MCG, Torres RB, Prata E, et al. Disturbances, elevation, topography and spatial proximity drive vegetation patterns along an altitudinal gradient of a top biodiversity hotspot. Biodivers Conserv. 2013;22(12):2767–83. Available from: 10.1007/s10531-013-0553-x.

[84] Zapata‐Rios X, Brooks PD, Troch PA, McIntosh J, Guo Q. Influence of terrain aspect on water partitioning, vegetation structure and vegetation greening in high‐elevation catchments in northern New Mexico. Ecohydrol. 2016;9(5):782–95. Available from: 10.1002/eco.1674.

[85] Liu L, Wang Y, Wang Z, Li D, Zhang Y, Qin D, et al. Elevation-dependent decline in vegetation greening rate driven by increasing dryness based on three satellite NDVI datasets on the Tibetan Plateau. Ecol Indicat. 2019;107:305–16. Available from: 10.1016/j.ecolind.2019.105569.

[86] National Elevation Dataset [Internet]. United States Geological Survey; [last updated 2002; cited 2020 Apr 15]. Available from: https://www.sciencebase.gov/catalog/item/505a61cde4b0c8380cd71b8d.

[87] Henrich V, Krauss G, Götze C, Sandow C. Index DataBase: A Database for Remote Sensing Indices [Internet]. Institute of Crop Science and Resource Conservation; [last updated 2023; cited 2023 Feb 18]. Available from: https://www.indexdatabase.de/db/i.php.

[88] Myneni RB, Asrar G. Atmospheric effects and spectral vegetation indices. Remote Sens Environ. 1994;47(3):390–402. Available from: 10.1016/0034-4257(94)90106-6.

[89] Rhyma PP, Norizah K, Hamdan O, Faridah-Hanum I, Zulfa AW. Integration of normalised different vegetation index and Soil-Adjusted Vegetation Index for mangrove vegetation delineation. Remote Sens App: Soc and Environ. 2020;17:100280. Available from: 10.1016/j.rsase.2019.100280.

[90] Young A, Selwood K, Benshemesh J, Wright J, Southwell D. Remotely sensed vegetation productivity predicts breeding activity and drought refuges for a threatened bird in semi‐arid Australia. Anim Conserv. 2022;25(4):566–81. Available from: 10.1111/acv.12763.

[91] Huete A, Justice C, Liu H. Development of vegetation and soil indices for MODIS-EOS. Remote Sens Environ. 1994;49(3):224–34. Available from: doi: 10.1016/0034-4257(94)90018-3

[92] Wang F-M, Huang J-F, Tang Y-L, Wang X-Z. New vegetation index and its application in estimating leaf area index of rice. Rice Sci. 2007;14(3):195–203. Available from: 10.1016/S1672-6308(07)60027-4.

[93] Huang Z, Liu X, Jin M, Ding C, Jiang J, Wu L. Deriving the characteristic scale for effectively monitoring heavy metal stress in rice by assimilation of GF-1 data with the wofost model. Sensors. 2016;16(3):340. Available from: doi: 10.3390/s16030340 26959033 PMC4813915

[94] Rumora L, Majić I, Miler M, Medak D. Spatial video remote sensing for urban vegetation mapping using vegetation indices. Urban Ecosyst. 2021;24(1):21–33. Available from: 10.1007/s11252-020-01002-5.

[95] Song Y, Huang B, Cai J, Chen B. Dynamic assessments of population exposure to urban greenspace using multi-source big data. Sci Total Environ. 2018;634(2018):1315–25. Available from: doi: 10.1016/j.scitotenv.2018.04.061 29710631

[96] Huck A, Hese S, Banzhaf E. Delineating parameters for object-based urban structure mapping in Santiago de Chile using QuickBird data. Int Arch Photogramm Remote Sens Spat Inf Sci. 2011;38(4/W19):131–6. Available from: 10.5194/isprsarchives-XXXVIII-4-W19-131-2011.

[97] Lee G, Hwang J, Cho S. A novel index to detect vegetation in urban areas using UAV-based multispectral images. Appl Sci. 2021;11(8):3472. Available from: 10.3390/app11083472.

[98] Spectral Indices [Internet]. NV5 Geospatial; [last updated 2023; cited 2023 May 26]. Available from: https://www.l3harrisgeospatial.com/docs/spectralindices.html.

[99] Hall-Beyer M. Practical guidelines for choosing GLCM textures to use in landscape classification tasks over a range of moderate spatial scales. Int J of Remot Sens. 2017;38(5):1312–38. Available from: 10.1080/01431161.2016.1278314.

[100] Coreau A, Martin J-L. Multi-scale study of bird species distribution and of their response to vegetation change: a Mediterranean example. Landsc Ecol. 2007;22(5):747–64. Available from: 10.1007/s10980-006-9074-2.

[101] Watson SJ, Luck GW, Spooner PG, Watson DM. Land-use change: incorporating the frequency, sequence, time span, and magnitude of changes into ecological research. Front Ecol Environ. 2014;12(4):241–9. Available from: 10.1890/130097.

[102] Baker-Gabb DJ, Hurley VG. National Recovery Plan for the Regent Parrot (eastern subspecies) Polytelis anthopeplus monarchoides. Melbourne, Australia: Department of Sustainability and Environment Melbourne; 2011.

[103] Mas JF. Monitoring land-cover changes: a comparison of change detection techniques. Int J of Remot Sens. 1999;20(1):139–52. Available from: 10.1080/014311699213659.

[104] Microsoft. Microsoft Office Professional Plus v. 17. Redmond, WA: Microsoft Corporation; 2020.

[105] Baguette M, Van Dyck H. Landscape connectivity and animal behavior: functional grain as a key determinant for dispersal. Landsc Ecol. 2007;22:1117–29. Available from: 10.1007/s10980-007-9108-4.

[106] Bellamy C, Scott C, Altringham J. Multiscale, presence‐only habitat suitability models: Fine‐resolution maps for eight bat species. J Appl Ecol. 2013;50(4):892–901. Available from: 10.1111/1365-2664.12117.

[107] Alobaidli S, McQuaid S, South C, Prakash V, Evans P, Nisbet A. The role of texture analysis in imaging as an outcome predictor and potential tool in radiotherapy treatment planning. Br J Radiol. 2014;87(1042):e20140369. Available from: doi: 10.1259/bjr.20140369 25051978 PMC4170870

[108] Materka A, Strzelecki M. Texture analysis methods–a review. Brussels: Technical University of lodz Institute of Electronics; 1998. Report No.: 4968.

[109] Hall-Beyer M. GLCM Texture: A Tutorial v. 3.0 [Internet]. University of Calgary; [last updated 2017; cited 2022 Mar 18]. Available from: https://prism.ucalgary.ca/bitstream/handle/1880/51900/texture20tutorial20v203_020180206.pdf?sequence=11.

[110] Zvoleff A. GLCM: An R Package v. Arlington, VA: Conservation International; 2020.

[111] Haralick RM, Shanmugam K, Dinstein IH. Textural features for image classification. IEEE Transact Systms, Man, and Cybernets. 1973;SMC-3(6):610–21. Available from: 10.1109/TSMC.1973.4309314.

[112] Schnase JL, Carroll ML, Gill RL, Tamkin GS, Li J, Strong SL, et al. Toward a Monte Carlo approach to selecting climate variables in MaxEnt. PLOS One. 2021;16(3):1–17. Available from: 10.1371/journal.pone.0237208. PMC7928495 33657125

[113] Räsänen O, Pohjalainen J, editors. Random subset feature selection in automatic recognition of developmental disorders, affective states, and level of conflict from speech. Proc of the Conference of Interspeech; Lyon, France: 2013 Aug 25–29.

[114] Guyon I, Elisseeff A. An introduction to variable and feature selection. J Machin Learn Res. 2003;3:1157–82. Available from: 10.1162/153244303322753616.

[115] Tracy JL, Kantola T, Baum KA, Coulson RN. Distribution and phenology of monarch butterfly larvae and their milkweed hosts in the South Central US. Biodivers Conserv. 2022;31(7):1797–827. Available from: 10.1007/s10531-022-02432-7.

[116] Bozdogan H. Model selection and Akaike’s information criterion (AIC): The general theory and its analytical extensions. Psychometrika. 1987;52(3):345–70. Available from: 10.1007/BF02294361.

[117] Warren DL, Seifert SN. Ecological niche modeling in Maxent: the importance of model complexity and the performance of model selection criteria. Ecol Appl. 2010;21(2):335–42. Available from: 10.1890/10-1171.1. 21563566

[118] Liu C, White M, Newell G. Selecting thresholds for the prediction of species occurrence with presence‐only data. J Biogeogr. 2013;40(4):778–89. Available from: doi: 10.1111/jbi.12058

[119] Elliott LF, Treuer-Kuehn A, Blodgett CF, True CD, German D, Diamond DD, cartographers. Ecological Systems of Texas: 391 Mapped Types. Phase 1–6, 10-meter resolution Geodatabase, Interpretive Guides, and Technical Type Descriptions. Austin, TX: Texas Parks & Wildlife Department and Texas Water Development Board; 2014.

[120] Stotz DF, Fitzpatrick JW, Parker TA III, Moskovits DK. Neotropical Birds: Ecology and Conservation. Chicago, IL: University of Chicago Press; 1996.

[121] Garrett KL. Population status and distribution of naturalized parrots in southern California. Western Birds. 1997;28(4):181–95.

[122] Enkerlin-Hoeflich EC. Comparative Ecology and Reproductive Biology of Three Species of *Amazona* Parrots in Northeastern Mexico. Doctoral Dissertation. Texas A&M University; 1995.

[123] Chen D, Huang J, Jackson TJ. Vegetation water content estimation for corn and soybeans using spectral indices derived from MODIS near-and short-wave infrared bands. Remote Sens Environ. 2005;98(2–3):225–36. Available from: 10.1016/j.rse.2005.07.008.

[124] Papeş M, Peterson AT, Powell GVN. Vegetation dynamics and avian seasonal migration: clues from remotely sensed vegetation indices and ecological niche modelling. J Biogeogr. 2012;39(4):652–64. Available from: 10.1111/j.1365-2699.2011.02632.x.

[125] Hille D. Conservation of Neotropical Parrots: Population Responses to Forest Cover Change and Trade Pressure and a Tool to Predict Sensitivity to Deforestation. Doctoral Dissertation. University of Oklahoma; 2022.

[126] Segura-Seqyeura D, editor Mapping the distribution range of the Red-fronted Parrotlet (*Touit costaricensis*) and evaluating its protection in Costa Rica. Proc of the Conference of the National Ornithological Association; Virtual: 2020.

[127] Sapiens MMG. Spatiotemporal variation of NDVI and EVI and its suitability to model Yuma Clapper Rail detections in the Cienega de Santa Clara, Sonora, Mexico. Doctoral Dissertation. University of Arizona; 2014.

[128] Matuzak GD, Bezy MB, Brightsmith DJ. Foraging ecology of parrots in a modified landscape: seasonal trends and introduced species. Wilson J Ornithol. 2008;120(2):353–65. Available from: 10.1676/07-038.1.

[129] Gibson L, Barrett B, Burbidge A. Dealing with uncertain absences habitat modelling: a case study rare ground-dwelling parrot. Divers Distrib. 2007;13:704–13. Available from: 10.1111/j.1472-4642.2007.00365.x.

[130] Bonebrake TC, Beissinger SR. Predation and infanticide influence ideal free choice by a parrot occupying heterogeneous tropical habitats. Oecologia. 2010;163:385–93. doi: 10.1007/s00442-010-1566-8 20135326 PMC2871107

[131] St‐Louis V, Pidgeon AM, Clayton MK, Locke BA, Bash D, Radeloff VC. Satellite image texture and a vegetation index predict avian biodiversity in the Chihuahuan Desert of New Mexico. Ecography. 2009;32(3):468–80. Available from: 10.1111/j.1600-0587.2008.05512.x.

[132] Enkerlin Hoeflich EC, Hogan KM. Red-crowned Parrot (*Amazona viridigenalis*). Birds of the World [Internet]. 2020. [cited 2022 Mar 15]. Available from: https://birdsoftheworld.org/bow/species/recpar/cur/introduction?login.

[133] Tamungang SA, Onabid MA, Awa T, Balinga VS. Habitat preferences of the Grey Parrot in heterogeneous vegetation landscapes and their conservation implications. Intl J Biodivers. 2016;2016:1–10. Available from: 10.1155/2016/7287563.

[134] Menkhorst PW, Loyn RH, Brown PB, editors. Management of the Orange-bellied Parrot. Proc of the Conference of Management and Conservation of Small Populations; Melborne, Australia: 1990 Sept 26–27.

[135] Burbidge AH, Blyth J, Danks A, Gillen K, Newbey B. Western Ground Parrot Interim Recovery Plan 1996–1999. Perth, Western Australia: Department of Conservation and Land Management; 1997. Report No.: Plan 6.

[136] Wood EM, Pidgeon AM, Radeloff VC, Keuler NS. Image texture predicts avian density and species richness. PLOS One. 2013;8(5):e63211:1–14. Available from: doi: 10.1371/journal.pone.0063211 23675463 PMC3651168

[137] Campos LFAS Teixeira BP, Efe MA. The importance of isolated patches for maintaining local bird biodiversity and ecosystem function: a case study from the Pernambuco Center of Endemism, Northeast Brazil. Iheringia. 2018;108:e2018021. Available from: 10.1590/1678-4766e2018021.

[138] Tucker MA, Alexandrou O, Bierregaard RO Jr, Bildstein KL, Böhning‐Gaese K, Bracis C, et al. Large birds travel farther in homogeneous environments. Glob Ecol Biogeogr. 2019;28(5):576–87. Available from: 10.1111/geb.12875.

[139] Jetz W, Thomas GH, Joy JB, Hartmann K, Mooers AO. The global diversity of birds in space and time. Nat. 2012;491(7424):444–8. Available from: doi: 10.1038/nature11631 23123857

[140] Brightsmith DJ, Hobson EA, Martinez G. Food availability and breeding season as predictors of geophagy in Amazonian parrots. Ibis. 2018;160(1):112–29. Available from: doi: 10.1111/ibi.12515

[141] Hill MJ. Vegetation index suites as indicators of vegetation state in grassland and savanna: An analysis with simulated SENTINEL 2 data for a North American transect. Remote Sens Environ. 2013;137(Oct):94–111. Available from: 10.1016/j.rse.2013.06.004.

[142] Masemola C, Cho MA, Ramoelo A. Sentinel-2 time series based optimal features and time window for mapping invasive Australian native Acacia species in KwaZulu Natal, South Africa. Intl J Appl Earth Observ Geoinfo. 2020;93(2020):e102207. Available from: 10.1016/j.jag.2020.102207.

[143] Morrison EB, Lindell CA, Holl KD, Zahawi RA. Patch size effects on avian foraging behaviour: implications for tropical forest restoration design. J Appl Ecol. 2010;47(1):130–8. Available from: 10.1111/j.1365-2664.2009.01743.x.

[144] Silva CM, Pereira JAC, Gusmões JDSP, Mendes BEP, Valente, Morgan AP, et al. Birds’ gap-crossing in open matrices depends on landscape structure, tree size, and predation risk. Perspect Ecol Conserv. 2020;18(2):73–82. Available from: 10.1016/j.pecon.2020.02.001.

[145] Kalodimos NP. The status and comparative nesting phenology of the Red-crowned Parrot on O’ahu, Hawaii. ’Elepaio. 2013;73(4):1–3.

[146] Opdam P, Wiens JA. Habitat loss and landscape management. In: Norris K, Pain DJ, editors. Conserving Bird Biodiversity: General Principles and Their Management. Conservation Biology. New York City, NY: Cambridge University Press; 2002.

[147] Martin PS, Robins CR, Heed WB. Birds and biogeography of the Sierra de Tamaulipas, an isolated pine-oak habitat. Wilson Bull. 1954;66(1):38–57.

[148] Nedd R, Light K, Owens M, James N, Johnson E, Anandhi A. A synthesis of land use/land cover studies: Definitions, classification systems, meta-studies, challenges and knowledge gaps on a global landscape. Land. 2021;10(9):1–30. Available from: 10.3390/land10090994.

[149] Chapman CA, Chapman LJ, Lefebvre L. Variability in parrot flock size: possible functions of communal roosts. Condor. 1989;91:842–7. Available from: 10.2307/1368068.

[150] Monterrubio-Rico TC, Cruz Nieto J, Enkerlin-Hoeflich E, Venegas Holguín D, Tellez García L, Marin Togo C. Gregarious nesting behavior of thick-billed parrots, (*Rhynchopsitta pachyrhyncha*) in aspen stands. Wilson J Ornithol. 2006;118:237–43. Available from: 10.1676/05-039.1.

[151] Gilardi JD, Munn CA. Patterns of activity, flocking, and habitat use in parrots of the Peruvian Amazon. Condor. 1998;100:641–53. Available from: doi: 10.2307/1369745

[152] Butler CJ. Feral parrots in the continental United States and United Kingdom: past, present, and future. J Avian Med Surg. 2005;19(2):142–9. Available from: 10.1647/183.

[153] Freile JF, Salas JA, Solano-Ugalde A, Navarrete R. Brotogeris versicolorus (Statius Müller, 1776) (Aves: Psittacidae): introduced established population in Ecuador. Check List. 2012;8:572–4. Available from: 10.15560/8.3.572.

[154] Morgan DHW. Feral Rose-ringed Parakeets in Britain. British Birds. 1993;86:561–4.

[155] Luna A, Romero-Vidal P, Hiraldo F, Tella JL. Cities may save some threatened species but not their ecological functions. PeerJ. 2018;6(e4908):1–22. Available from: 10.7717/peerj.4908. PMC6016529 29942675

[156] Rycken SJE, Warren KS, Yeap L, Donaldson R, Mawson P, Dawson R, et al. Forest specialist species in the urban landscape: do different levels of urbanization affect the movements of Forest Red-tailed Black Cockatoos (*Calyptorhynchus banksii naso*)? Avian Conserv Ecol. 2022;17(1):1–17. Available from: 10.5751/ACE-02061-170111.

[157] Wang S. Establishment of an introduced population of critically endangered Yellow-crested Cockatoo (*Cacatua sulphurea*) in Hong Kong. Doctoral Dissertation. Hong Kong University of Science and Technology; 2020.

[158] Robles JAMZ Sumagaysay SAGGS, Rose E Young BP, Oberio ZL. The relationship between urbanization indicators and the *Passer montanus* bird count in urban and urban sprawl areas in Iloilo and Bacolod, Philippines. Publisci. 2022;5(1):8–13.

[159] Lepczyk CA, Aronson MFJ, Evans KL, Goddard MA, Lerman SB, MacIvor JS. Biodiversity in the city: fundamental questions for understanding the ecology of urban green spaces for biodiversity conservation. Biosci. 2017;67(9):799–807. Available from: 10.1093/biosci/bix079.

[160] Gomez R, Guajardo L, Ely-Ledesma E. It is time to recognize the Rio Grande Valley as a rising borderland metropolis. The Urban Edge [Internet]. 2022 Jun 15. Available from: https://kinder.rice.edu/urbanedge/it-time-recognize-rio-grande-valley-rising-borderland-metropolis.

[161] De La Rosa P. As SpaceX ramps up activity in the Rio Grande Valley, local concerns grow. Texas Public Radio [Internet]. 2021 May 12. Available from: https://www.tpr.org/border-immigration/2021-05-12/spacex-rio-grande-valley-local-concerns-grow.

[162] Wattles J, Fisher K. SpaceX’s interplanetary rocket fires up engines in unprecedented test. CNN [Internet]. 2023 Feb 10. Available from: https://www.cnn.com/2023/02/09/business/spacex-static-fire-starship-super-heavy-scn.

[163] Sandström UG, Angelstam P, Mikusiński G. Ecological diversity of birds in relation to the structure of urban green space. Landsc Urban Plan. 2006;77(1–2):39–53. Available from: 10.1016/j.landurbplan.2005.01.004.

[164] Khera N, Mehta V, Sabata BC. Interrelationship of birds and habitat features in urban greenspaces in Delhi, India. Urban For Urban Green. 2009;8(3):187–96. Available from: 10.1016/j.ufug.2009.05.001.

[165] de Toledo MCB, Donatelli RJ, Batista GT. Relation between green spaces and bird community structure in an urban area in southeast Brazil. Urban Ecosyst. 2012;15(1):111–31. Available from: 10.1007/s11252-011-0195-2.

[166] Carbó-Ramírez P, Zuria I. The value of small urban greenspaces for birds in a Mexican city. Landsc Urban Plan. 2011;100(3):213–22. Available from: 10.1016/j.landurbplan.2010.12.008.

[167] Chamberlain DE, Gough S, Vaughan H, Vickery JA, Appleton GF. Determinants of bird species richness in public green spaces. Bird Study. 2007;54(1):87–97. Available from: 10.1080/00063650709461460.

[168] Dale S. Urban bird community composition influenced by size of urban green spaces, presence of native forest, and urbanization. Urban Ecosyst. 2018;21(1):1–14. Available from: 10.1007/s11252-017-0706-x.

[169] Champness BS, Palmer GC, Fitzsimons JA. Bringing the city to the country: relationships between streetscape vegetation type and bird assemblages in a major regional centre. J Urban Ecol. 2019;5(1):1–11. Available from: 10.1093/jue/juz018.

[170] Semeraro T, Scarano A, Buccolieri R, Santino A, Aarrevaara E. Planning of urban green spaces: An ecological perspective on human benefits. Land. 2021;10(2):105. Available from: 10.3390/land10020105.

[171] Jorgensen A, Gobster PH. Shades of green: measuring the ecology of urban green space in the context of human health and well-being. Nat Cultur. 2010;5(3):338–63. Available from: 10.3167/nc.2010.050307.

[172] Sulaiman S, Mohamad NHN, Idilfitri S. Contribution of vegetation in urban parks as habitat for selective bird community. Proc Soc Behav Sci. 2013;85:267–81. Available from: 10.1016/j.sbspro.2013.08.358.

[173] Mabb KT. Roosting behavior of the naturalized parrots in San Gabriel Valley, California. Western Birds. 1997;28:202–8.

[174] Guzmán JCC, Saldaña MES, De la Puente EG, Ontiveros JMP. Parrot Illegal Trade Decreases in Mexico. Washington, D.C.: Defenders of Wildlife; 2022.

[175] Rushing CS, Royle JA, Ziolkowski DJ Jr, Pardieck KL. Migratory behavior and winter geography drive differential range shifts of eastern birds in response to recent climate change. Proc Natl Acad Sci USA. 2020;117(23):12897–903. Available from: doi: 10.1073/pnas.2000299117 32457137 PMC7293646

[176] Radchuk V, Reed T, Teplitsky C, van de Pol M, Charmantier A, Hassall C, et al. Adaptive responses of animals to climate change are most likely insufficient. Nat Commun. 2019;10(1):3109. Available from: doi: 10.1038/s41467-019-10924-4 31337752 PMC6650445

[177] Weisser WW, Hauck TE. Animal-aided design–using a species’ life-cycle to improve open space planning and conservation in cities and elsewhere. J Urban Ecol. 2017;2017(June):1–14. Available from: 10.1101/150359.

[178] Fontana S, Sattler T, Bontadina F, Moretti M. How to manage the urban green to improve bird diversity and community structure. Landsc Urban Plan. 2011;101(3):278–85. Available from: 10.1016/j.landurbplan.2011.02.033.

[179] MacGregor-Fors I, Schondube JE. Gray vs. green urbanization: relative importance of urban features for urban bird communities. Basic Appl Ecol. 2011;12(4):372–81. Available from: 10.1016/j.baae.2011.04.003.

[180] Spotswood EN, Beller EE, Grossinger R, Grenier JL, Heller NE, Aronson MF. The biological deserts fallacy: cities in their landscapes contribute more than we think to regional biodiversity. Biosci. 2021;71(2):148–60. Available from: doi: 10.1093/biosci/biaa155 33613128 PMC7882369

[181] Thomson G, Newman P, Hes D, Bennett J, Taylor M, Johnstone R. Nature-Positive design and development: a case study on tegenerating Black Cockatoo habitat in urban developments in Perth, Australia. Urb Sci. 2022;6(3):1–22. Available from: 10.3390/urbansci6030047.

[182] Wiedenfeld DA, Alberts AC, Angulo A, Bennett EL, Byers O, Contreras‐MacBeath T, et al. Conservation resource allocation, small population resiliency, and the fallacy of conservation triage. Conserv Biol. 2021;35(5):1388–95. Available from: doi: 10.1111/cobi.13696 33484006 PMC8518633

[183] Miskelly C, Powlesland R. Conservation translocations of New Zealand birds, 1863–2012. Notornis. 2013;60(1):3–28.

