# Supplementary material for: Modelling Red–Crowned Parrot (Psittaciformes: Amazona viridigenalis [Cassin, 1853]) distributions in the Rio Grande Valley of Texas using elevation and vegetation indices and their derivatives
Source: PLoS One. 2023 Dec 6;18(12):e0294118. doi: 10.1371/journal.pone.0294118 (PMC10699612; doi:10.1371/journal.pone.0294118)
Supplement: S1 File — This file contains the supplementary materials associated with the manuscript entitled “Modelling Red-crowned Parrot (Psittaciformes: Amazona viridigenalis [Cassin, 1853]) distributions in the Rio Grande Valley of Texas using elevation and vegetation indices and their derivatives”. (DOCX) [file pone.0294118.s001.docx]

S1 File. Supplementary Materials

Resumen

Los loros tamaulipecos del Valle del Río Grande de Texas (Psittaciformes: *Amazona viridigenalis* [Cassin, 1853]) ocupan principalmente áreas urbanas con vegetación en lugar de áreas naturales. Investigamos la utilidad de los índices de vegetación no procesados y sus derivados, así como la elevación en el modelado de las distribuciones de hábitat de uso general, de sitios de anidación y de sitios de descanso de estas poblaciones. Se usó un algoritmo de selección de características para crear y seleccionar un conjunto de modelos MaxEnt mejor clasificados y de escala fina a partir de subconjuntos decorrelacionados de tamaño óptimo de cuatro a siete de 199 variables potenciales. Las variables se clasificaron post hoc según la frecuencia de aparición y la importancia de la permutación media en los modelos mejor clasificados. Nuestros modelos de conjunto predijeron con precisión las distribuciones de interés (x ̅ Área bajo la curva [AUC] = 0.904–0.969). Las variables mejor clasificadas para diferentes modelos de distribución de hábitat incluyeron: (1) uso general: porcentaje de cobertura de rangos preferidos de textura de entropía de valores del Índice de vegetación de diferencia normalizada (NDVI), texturas de entropía y contraste de NDVI y elevación; (2) sitio de anidación: texturas de entropía de NDVI y NDVI verde-azul, y porcentaje de cobertura del rango preferido de textura de entropía de valores de NDVI; (3) sitio de descanso: porcentaje de cobertura de los rangos preferidos de textura de entropía de los valores de NDVI, textura de contraste de NDVI y textura de entropía del índice de diferencia normalizado verde-rojo. La presencia del loro tamaulipeco del Valle del Río Grande de Texas se asoció con áreas urbanas con alta heterogeneidad y aleatoriedad en la distribución de la vegetación y/o sus características (p. ej., arreglo, tipo, estructura). El mantenimiento de los tipos de vegetación ya existentes y preferidos por el loro y su incorporación en nuevos desarrollos urbanos podría favorecer la persistencia de los loros tamaulipecos en el sur de Texas.

Results: Variable rankings for 12 top-selected models

The performance of variables comprising the 12 top-selected models employed in the creation of the final projections, which were randomly selected from the 250 top-performing models, is a subset of the performance of the comprehensive set of variables in the 250 top-performing models. Consequently, rankings of variables only appearing in the 12 top-selected models are less important than rankings of variables in the entire set of the 250 top-performing models for each distribution of interest.

Suitable general use habitat distribution

Fifty-nine unique variables appeared in the 12 top-selected models used to derive the projections of the general use habitat distribution (S1 Table). Eleven variables occurred in more than one of the 12 top-selected models (*i.e.*, *elevation*, *ndvi10*, *ndv102v990*, *bnd082v990*, *ndv102e990*, *sar082e99070*, *grn102v99pc70*, *grn102v99pc70*, *grn102v99pc70*, *ndv102e99pc80*, and *deltgrn10*_08; S1 Table). Thirteen variables in the 12 top-selected models used to create the projection of this distribution also occurred in the 12 top-selected models used to create the final projections of the predicted nest site habitat distribution (*i.e.*, *elevation*, *gbn08*_*50*_*990*, *grn10*_70_990, *gbn082m310*, *grn102m990*, *gbn082v990*, *bnd082e990*, *bnd082m31070*, *grn102v31070*, *grn102e31070*, *ndv082v31pc70*, *ndv102e99pc70*, and *deltgrn10*_*08*; S1 Table). Eight variables co-occurred in the 12 top-selected models used to create the projection of the predicted roost site habitat distribution (*i.e.*, *sar08*_*70*_*990*, *sar082m990*, *grn102c990*, *ndv082e990*, *gbn082c99070*, *bnd082m31pc70*, *sar082m99pc70*, and *grn102m31pc70*; S1 Table). Three features co-occurred in the 12 top-selected used to create the projections of the predicted general use, nest site, and roost site habitat distributions (*i.e.*, *grndi10*, *ndv102299pc85*, and *grn102e99pc80*; S1 Table).

Second-order Grey-level Co-Occurrence (GLCM) entropy texture of raw 2010 Normalized Difference Vegetation Index (NDVI) using a focal window of 990 m (*i.e.*, *ndv102e990*) had the highest mean permutation importance (*i.e.*, 81.6±0.7 in any of the 12 top-selected models. It also had the highest mean permutation importance (*i.e.*, 83.7) of any variable in any one of these models; this variable occurred in three of the 12 top-selected models (S1 Table). *Elevation* appeared most frequently in top-selected models (n = 6), and its mean permutation importance was 51.1±0.3. The most common types of variables that occurred in top-selected models were raw vegetation index textures, percent cover preferred vegetation index textures, and preferred vegetation index textures (S1 Table). Although raw vegetation index variables occurred in the 12 top-selected models three times, their mean permutation importance failed to exceed 1.5. Similarly, although a raw vegetation index difference type variable occurred in the 12 top-selected models twice, it did not have high mean permutation importance on either occasion. Percent cover preferred vegetation index textures, raw vegetation index textures, and preferred vegetation index textures were the most common variable type that had a mean permutation importance greater than 10 in top-selected models. Raw vegetation index textures were the most common top-performing variable type. Normalized difference vegetation index, Soil- and Atmospherically-Resistant Vegetation Index (SARVI), and Green-Red Normalized Difference Index (GRNDI) were the most common type of vegetation indices used to derive variables that appeared in the 12 top-selected models; however, variables derived from SARVI and GRNDI had relatively lower permutation importance (*i.e.*, 2.0 and 1.9, respectively) compared to variables derived from other vegetation indices. Normalized difference vegetation index-derived features were the most common of those with a mean permutation importance greater than 10 and variables derived using this vegetation index were among the most common that appeared in the 12 top-selected models. Second-order GLCM entropy and variance texture derived variables were the most common that appeared in the 12 top-selected models; however, variance texture derived variables only had a mean permutation importance greater than 10 on four occasions. Second-order GLCM entropy texture derived variables were the most common of those with a permutation importance higher than 10 and were among the highest performing texture-based variables that appeared in the 12 top-selected models. Non-texture type variables had a mean permutation importance of more than 10 on seven occasions; six of these were repeat appearances of *elevation*. *Elevation* was a top-performing variable each time it occurred in the 12 top-selected models. Of variables whose derivation required the use of a moving window analysis, only those that were derived using a focal window size of 990 m a mean permutation importance of greater than 10.

Suitable nest site habitat distribution

Forty-four unique variables made up the 12 top-selected models used to create our final projections of the predicted nest site habitat distribution (S1 Table). Eight variables occurred in more than one of these 12 top-selected models (*i.e.*, *elevation*, *grndi08*, *grn102m990*, *bnd082e310*, *grn102m99070*, *ndv082m99pc70*, *bnd082v99pc70*, and *ndv102e99pc70*). Seven variables co-occurred in the 12 top-selected models used to create the projection of the predicted roost site habitat distribution (*i.e.*, *gbndvi08*, *gbn082v310*, *grn102m99070*, *sar082v31070*, *ndv082m99pc70*, *ndv082c99pc85*, and *ndv102c99pc85*; S1 Table).

Percent cover of the preferred range of 2^nd^-order GLCM contrast texture of raw 2010 NDVI values (central 85%) derived using a focal window size of 990 m (*i.e.*, *ndv102c99pc85*) had the highest mean permutation importance (*i.e.*, 63.2±0.0; S1 Table). Percent cover of the preferred range of 2^nd^-order GLCM entropy texture of raw 2010 NDVI values (central 70%) derived using a focal window size of 990 m (*i.e.*, *ndv102e99pc70*) had the highest permutation importance (*i.e.*, 72.9) in any one of the 12 top-selected models. The variable that appeared in the 12 top-selected models most frequently was *elevation* (n=5); its mean permutation importance was 39.9±8.8. The most common feature types that occurred in 12 top-selected models were 2^nd^-order GLCM textures of raw vegetation indices, percent cover of preferred ranges of 2^nd^-order GLCM textures of vegetation index values, and 2^nd^-order GLCM textures of preferred/non-preferred ranges of raw vegetation indices. Percent cover of preferred ranges of 2^nd^-order GLCM textures of vegetation index values, 2^nd^-order GLCM textures of raw vegetation indices, and preferred ranges of 2^nd^-order GLCM textures of raw vegetation index values were the most common variable types that had a mean permutation importance greater than 10 (S2 Table). Raw vegetation indices occurred in the 12 top-selected models four times; however, the maximum mean permutation importance of any one appearance in the top-selected models was 6.6±0.0 (S1 Table). Similarly, a multitemporal raw vegetation index difference feature occurred in one of the 12 top-selected models, but it did not have a high mean permutation importance (*i.e.*, <1.0). Normalized difference vegetation index was the most common vegetation index used to derive variables in the 12 top-selected models (S2 Table). Second-order GLCM entropy, mean, and variance textures were the most common textures types used to derive variables that appeared in the 12 top-selected models; however, variance texture derived variables only had a mean permutation importance higher than 10 on three occasions. Entropy texture derived variables were the most common of those with a mean permutation importance higher than 10; they were also the most common of top-performing variable type. Non-texture type variables had a mean permutation importance greater than 10 on only five occasions; all were repeat occurrences of *elevation.* *Elevation* was the top-performing variable on one occasion. Variables derived using both focal window sizes appeared in top-selected models at the same rate overall (n=12) and among those that had a mean permutation importance of more than 10 (S2 Table).

Suitable roost site habitat distribution

Thirty-five unique features were used in the models selected for use to derive the projections for the roost site distribution feature subset ensembles (S1 Table). Nine features occurred in more than one subset (*i.e.*, *ndv08*_*50*_*310*, *bnd082m310*, *gbn082v310*, *ndv082e990*, *gbn082c99070*, *grn102c31070*, *ndv102m31pc70*, *bnd082m31pc70*, and *grn102e99pc80*).

Percent cover of the preferred range of 2^nd^-order GLCM contrast texture of raw 2010 NDVI values (central 85%) derived using a focal window size of 990 m (*i.e.*, *ndv102c99pc85*) had the highest mean permutation importance (*i.e.*, 82.2±0.0) of all those that were used to derive the 12 top-selected models, however, it only occurred in one feature subset (S1 Table). The 2^nd^-order GLCM entropy texture of raw 2008 NDVI derived using a focal window size of 990 m (*i.e.*, *ndv082e990*) had the highest permutation importance of a variable that appeared in any one of the 12 top-selected models (*i.e.*, 83.9); it occurred in twice in the 12 top-selected models. Percent cover of preferred ranges of the entropy of raw 2010 GRNDI values (*i.e.*, central 80%) derived using a focal window size of 990 m (*i.e.*, *grn102e990pc80*) occurred most frequently (n=3) in the 12 top-selected models; its mean permutation importance was 11.9±9.4. The most common feature types that occurred in the 12 top-selected models were percent cover of preferred ranges of 2^nd^-order GLCM textures of raw vegetation index values, 2^nd^-order GLCM textures of raw vegetation indices, and 2^nd^-order GLCM textures of preferred/nonpreferred ranges of vegetation index values (S2 Table). Raw vegetation index features occurred in the 12 top-selected models three times, however, the maximum mean permutation importance of any one of its appearances in any of the 12 top-selected models was 12.2±0.0 (S1 Table). *Elevation* and multitemporal vegetation index difference type features did not occur in any of the feature subsets used to derive the 12 top-selected models. The most common feature type with a mean permutation importance greater than 10 that appeared in the top-selected models was percent cover of preferred ranges of 2^nd^-order GLCM textures of raw vegetation index values. Green-red normalized difference index and NDVI derived features were the most frequent of features derived from vegetation indices in the 12 top-selected models overall. Green-red normalized difference vegetation index and NDVI derived features were the most common of those with a permutation importance greater than 10 (S2 Table). Second-order GLCM contrast and mean texture-based features were the most common textures used to derive variables that appeared in the 12 top-selected models. Contrast features were the most common of those with a mean permutation importance higher than 10. Non-texture derived variables had a mean permutation importance of more than 10 on two occasions; however, they were never top-performing feature types in any of the 12 top-selected models. Features derived using the different window sizes occurred at around the same overall rate in the 12 top-selected models (n=21); however, features derived using a 990 m window were more common than those derived with a 310 m window among features with a mean permutation importance of more than 10 and those that appeared as the top-performing feature in top-selected models (S2 Table).
