# Supplementary figures and images for: Modelling Red–Crowned Parrot (Psittaciformes: Amazona viridigenalis [Cassin, 1853]) distributions in the Rio Grande Valley of Texas using elevation and vegetation indices and their derivatives
Source: PLoS One. 2023 Dec 6;18(12):e0294118. doi: 10.1371/journal.pone.0294118 (PMC10699612; doi:10.1371/journal.pone.0294118)

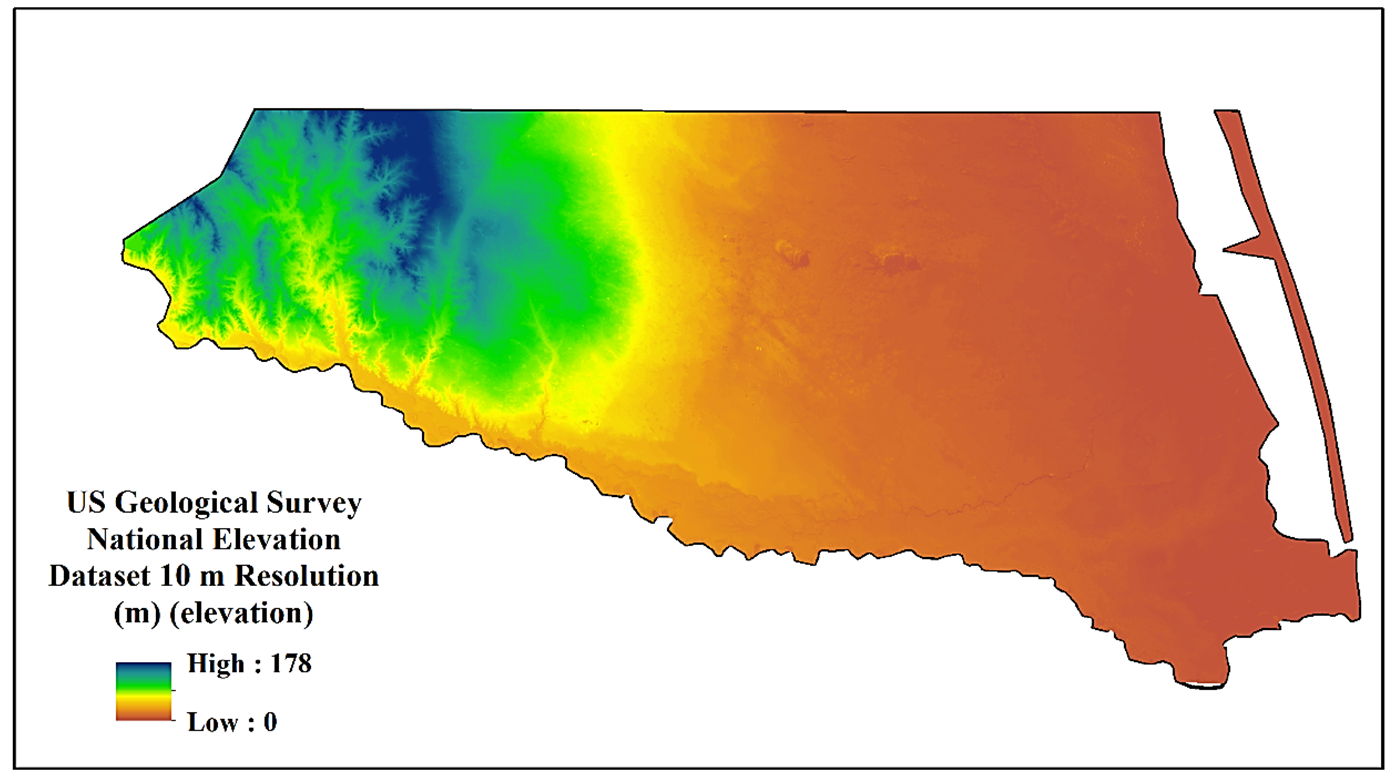

Supplement: S1 Fig — This variable, elevation, was derived using parts of the 10 m United States Geological National Elevation Survey dataset. (TIF) [file pone.0294118.s002.tif]

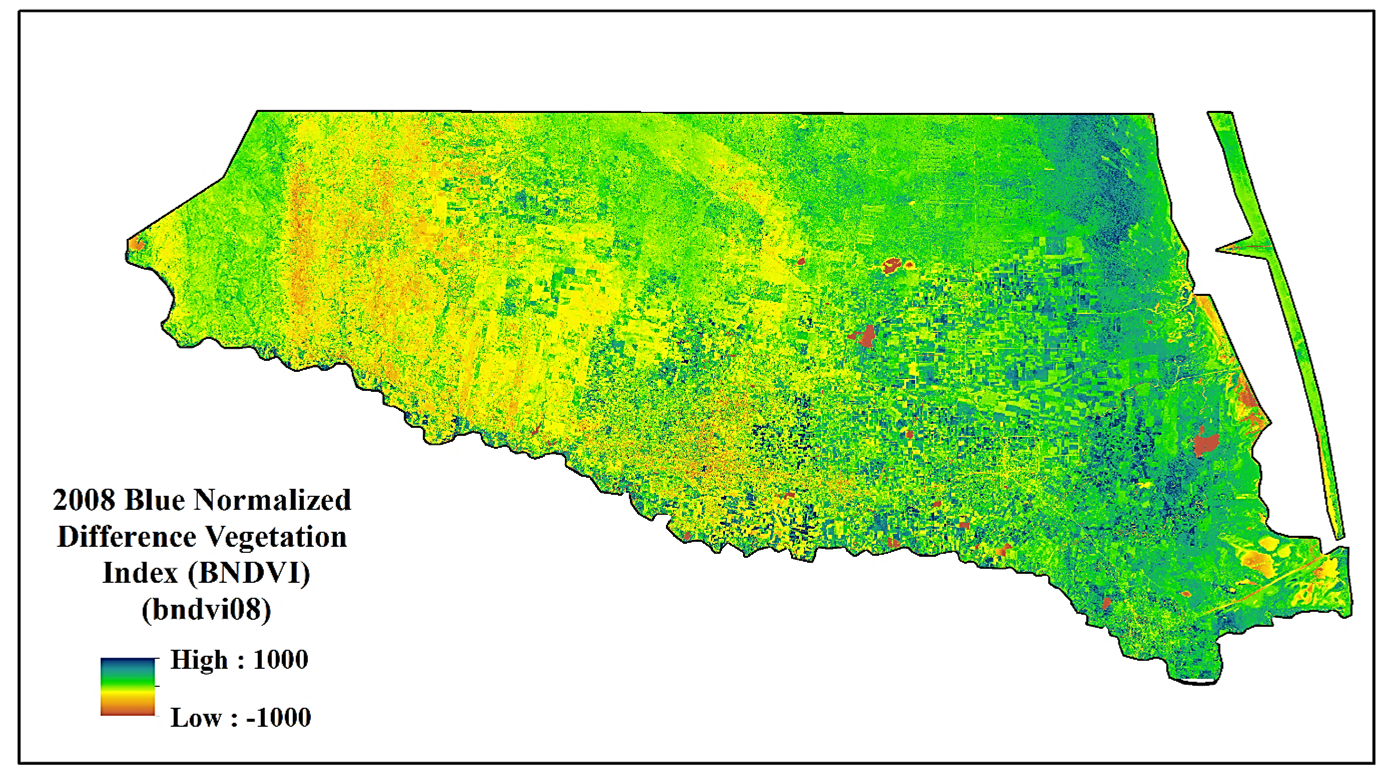

Supplement: S2 Fig — The variable raw 2008 Blue Normalized Difference Vegetation Index (BNDVI), bndvi08, was created by performing a simple calculation to the information contained in each of the three spectral bands of National Aerial Imagery Program (NAIP) images for 2008 (Table 1). (TIF) [file pone.0294118.s003.tif]

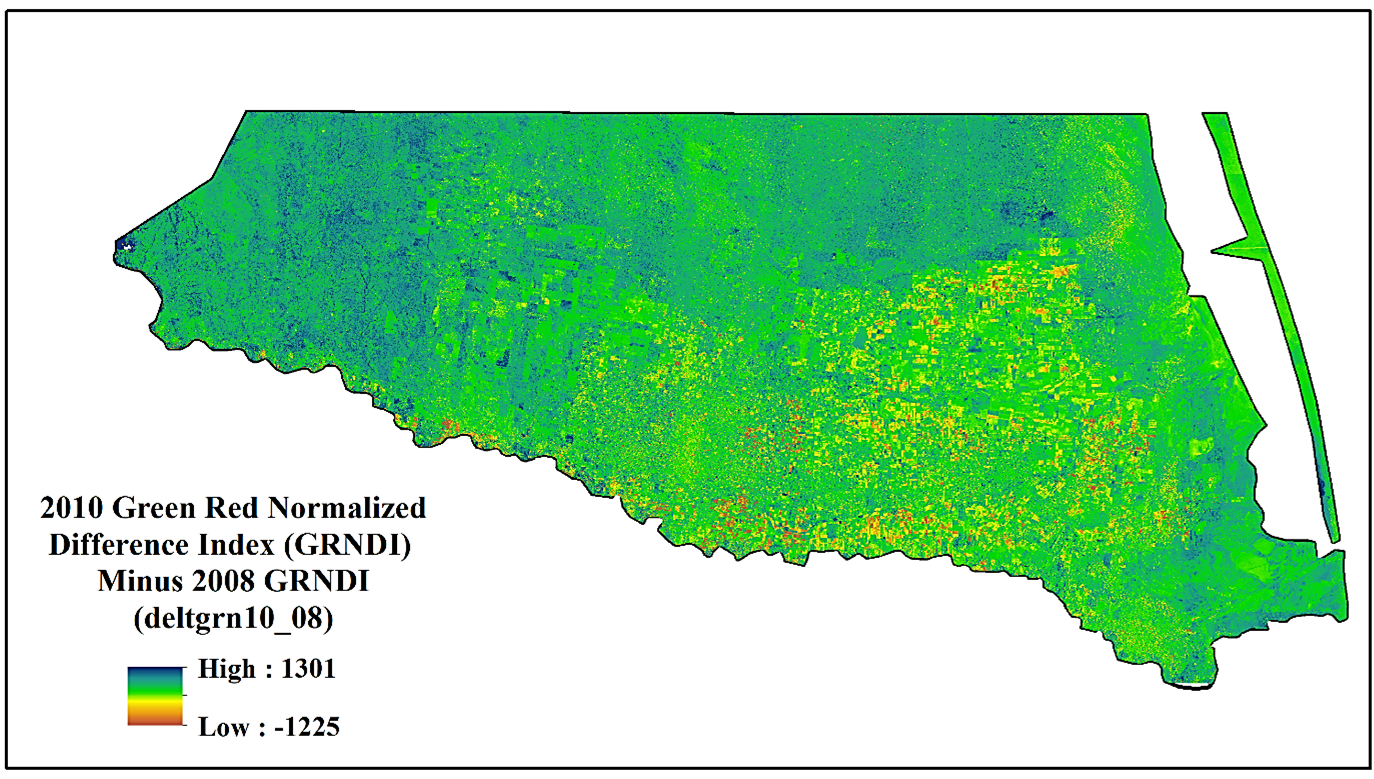

Supplement: S3 Fig — This variable, deltgrn10_08, was derived by subtracting raw 2008 Green Red Normalized Difference Index (GRNDI; grndi08) from raw 2010 GRNDI (grndi10). (TIF) [file pone.0294118.s004.tif]

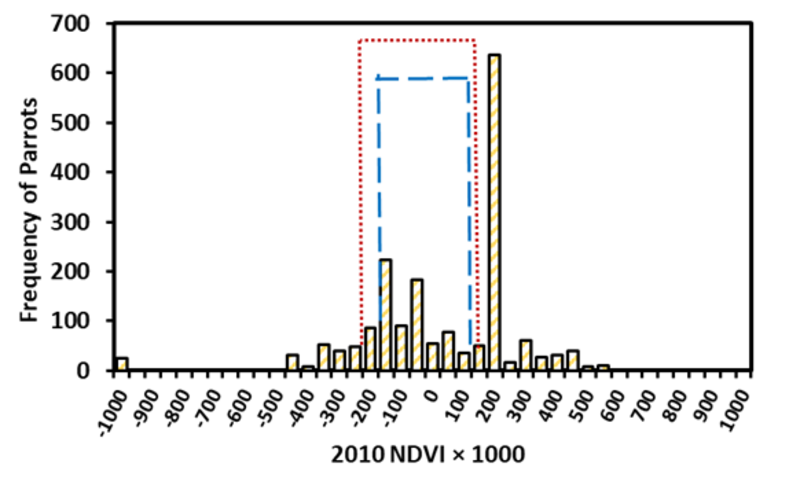

Supplement: S4 Fig — Blue dashed lines represent the central range (i.e., 50%) of raw 2010 Normalized Difference Vegetation Index (NDVI) values (i.e., -211 to 156) while red dotted lines represent the central range (i.e., 70%) of raw 2010 NDVI values (i.e., 191 to 155) of parrot presence points around the median raw 2010 NDVI value of 21. (TIF) [file pone.0294118.s005.tif]

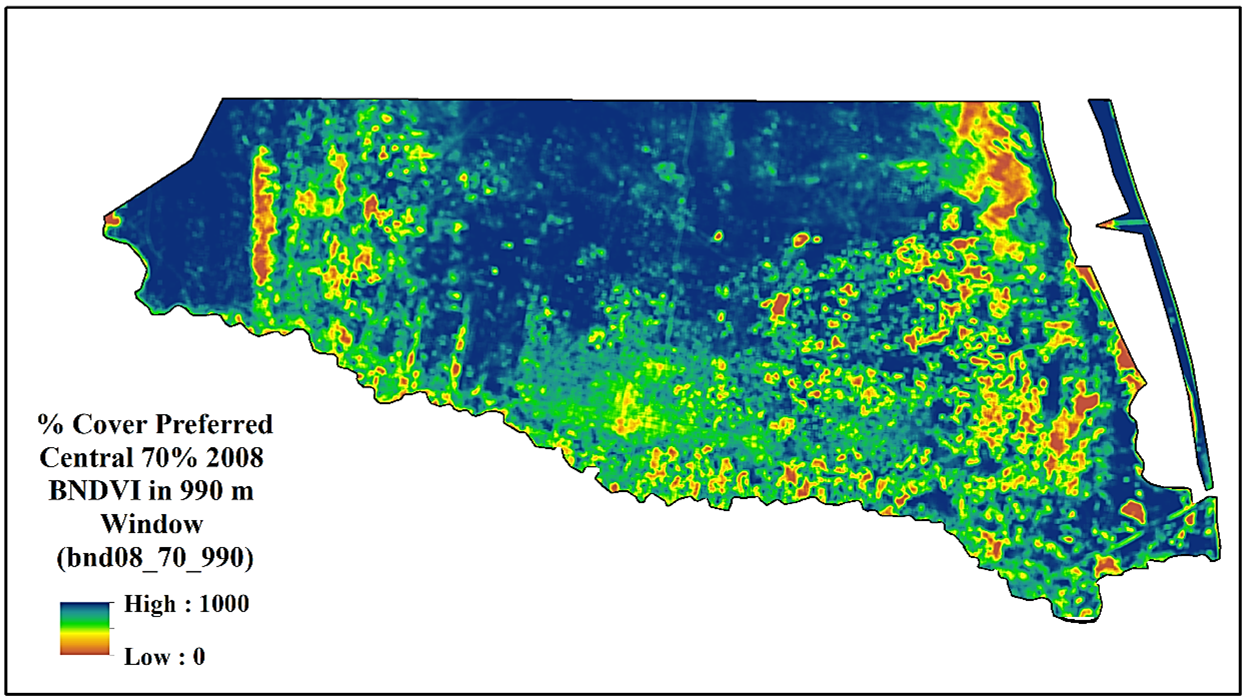

Supplement: S5 Fig — This variable, bndvi_70_990, depicts the percent cover of a preferred range of raw 2008 Blue Normalized Difference Vegetation Index (BNDVI; bndvi08) values (i.e., set of values associated with Red-crowned Parrot presence points about the median value). First, we calculated the preferred range of bndvi08 values (70%; see S4 Fig), which was then used to create a binary preferred/non-preferred raster. The final raster was created by applying a focal window analysis to the binary raster using a square 990 m focal window. (TIF) [file pone.0294118.s006.tif]

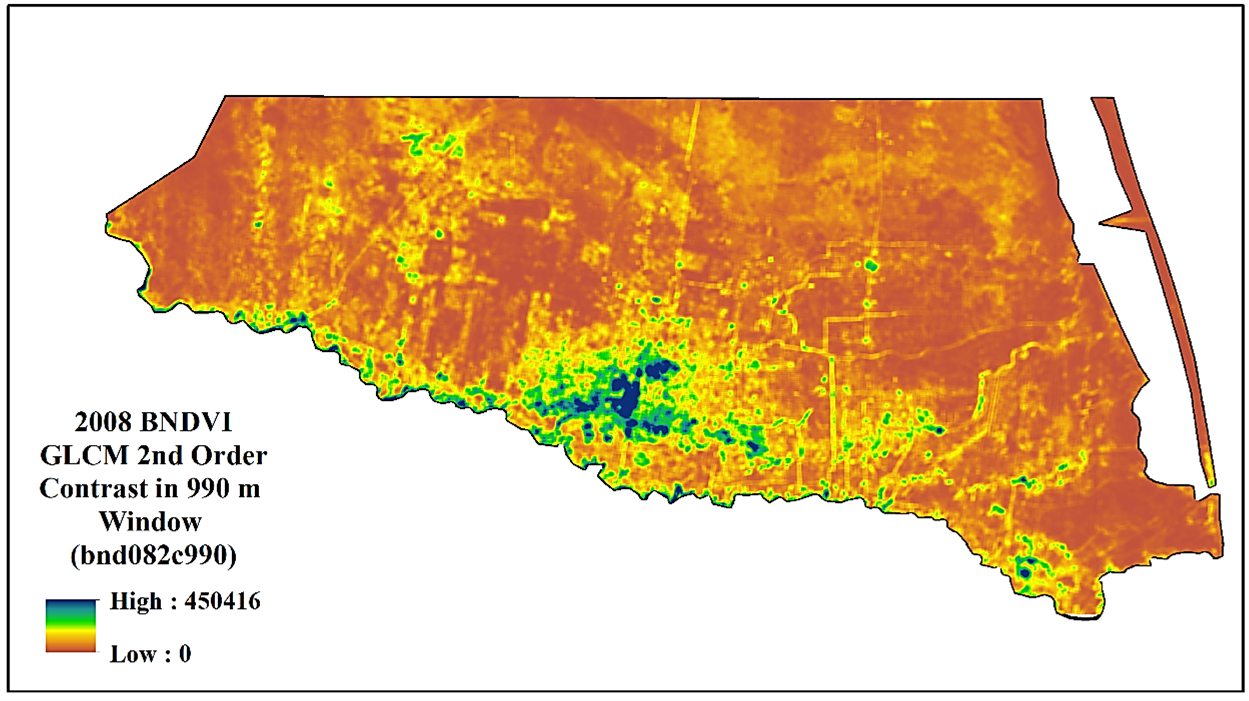

Supplement: S6 Fig — This variable, bnd082c990, represents the 2nd-order Gray Level Co-Occurrence Matrix (GLCM) contrast texture of raw 2008 Blue Normalized Difference Vegetation Index (BNDVI; bndvi08) values that was derived using a square 990 m focal window. (TIF) [file pone.0294118.s007.tif]

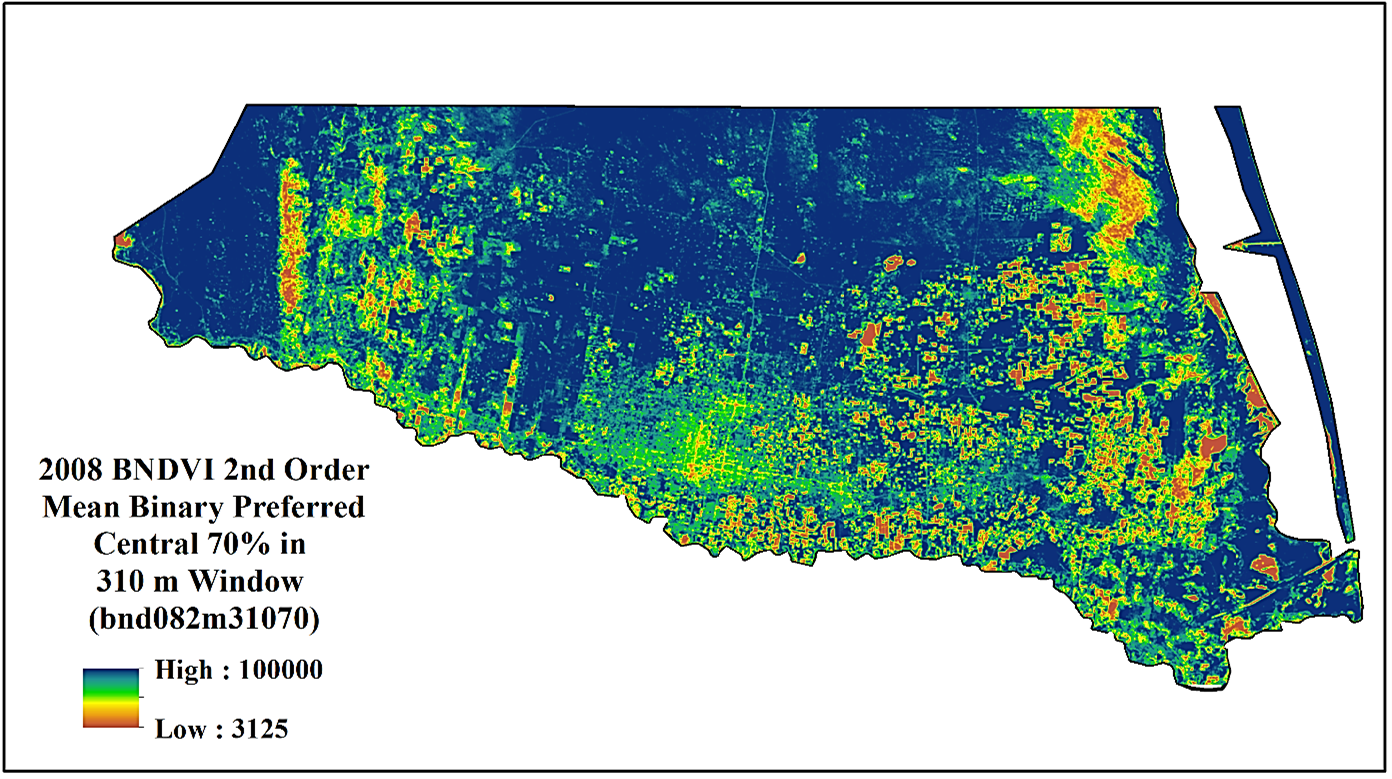

Supplement: S7 Fig — This variable, bnd08m70310, depicts the 2nd-order Grey Level Co-Occurrence Matrix (GLCM) mean texture of the binary preferred/nonpreferred range of raw 2008 Blue Normalized Difference Vegetation Index (BNDVI) values (i.e., central set of values associated with Red-crowned Parrot presence points about the median value). Deriving this variable involved (1) calculating the central range of raw 2008 BNDVI values (i.e., 70%; see S4 Fig), (2) creating a binary preferred/nonpreferred raster using the aforementioned ranges, and (3) calculating the 2nd-order GLCM mean texture for the binary raster using a square 990 m focal window. (TIF) [file pone.0294118.s008.tif]

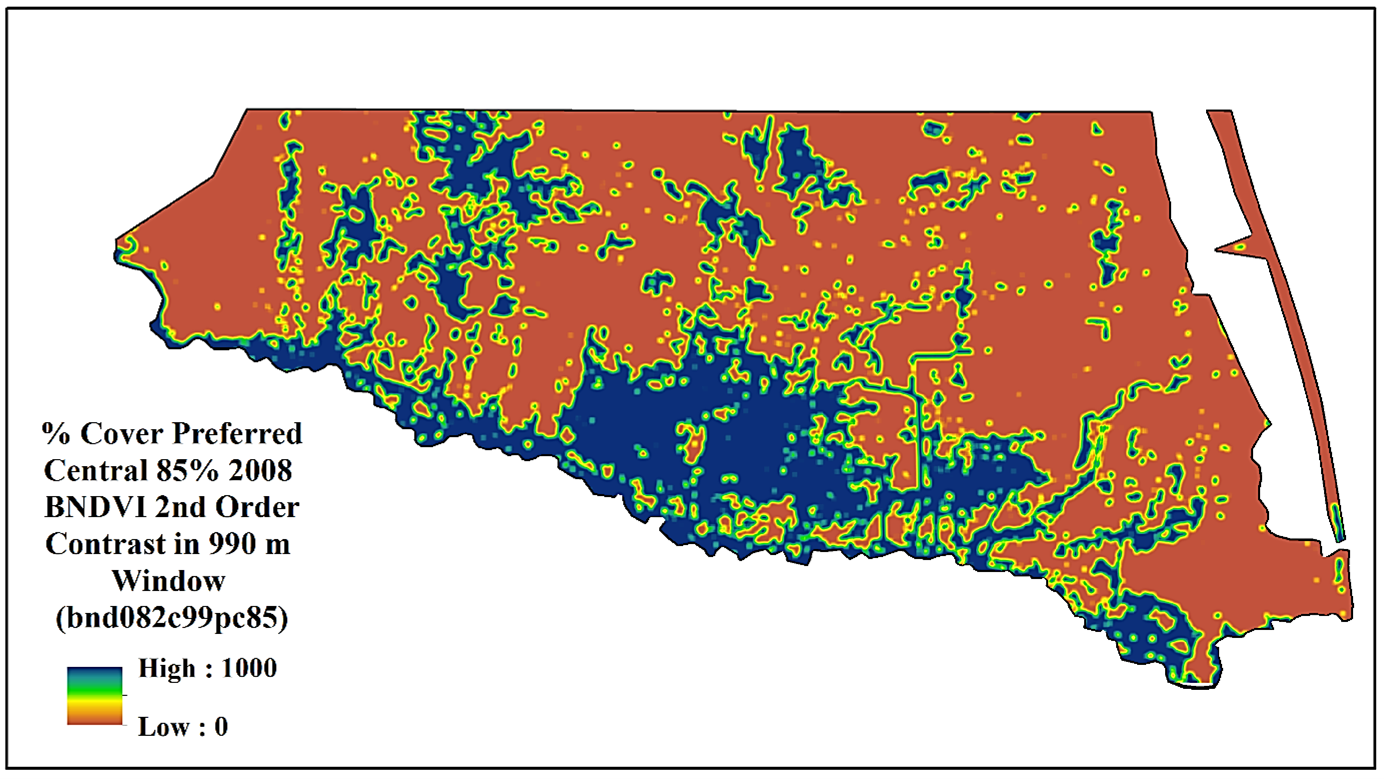

Supplement: S8 Fig — This variable, bnd082c99pc85, depicts the percent cover of preferred ranges of 2nd-order Grey Level Co-Occurrence Matrix (GLCM) contrast texture of raw 2008 Blue Normalized Difference Vegetation Index (BNDVI) values (i.e., set of values associated with Red-crowned Parrot presence points about the median value). Creating this variable involved (1) calculating the central range (i.e., 85%) of 2nd-order GLCM contrast texture of raw 2008 BNDVI values derived using a square 990 m focal window, (2) creating a binary preferred/non-preferred raster from this range, and (3) calculating the percent cover of preferred areas from the binary raster using a square 990 m focal window. (TIF) [file pone.0294118.s009.tif]

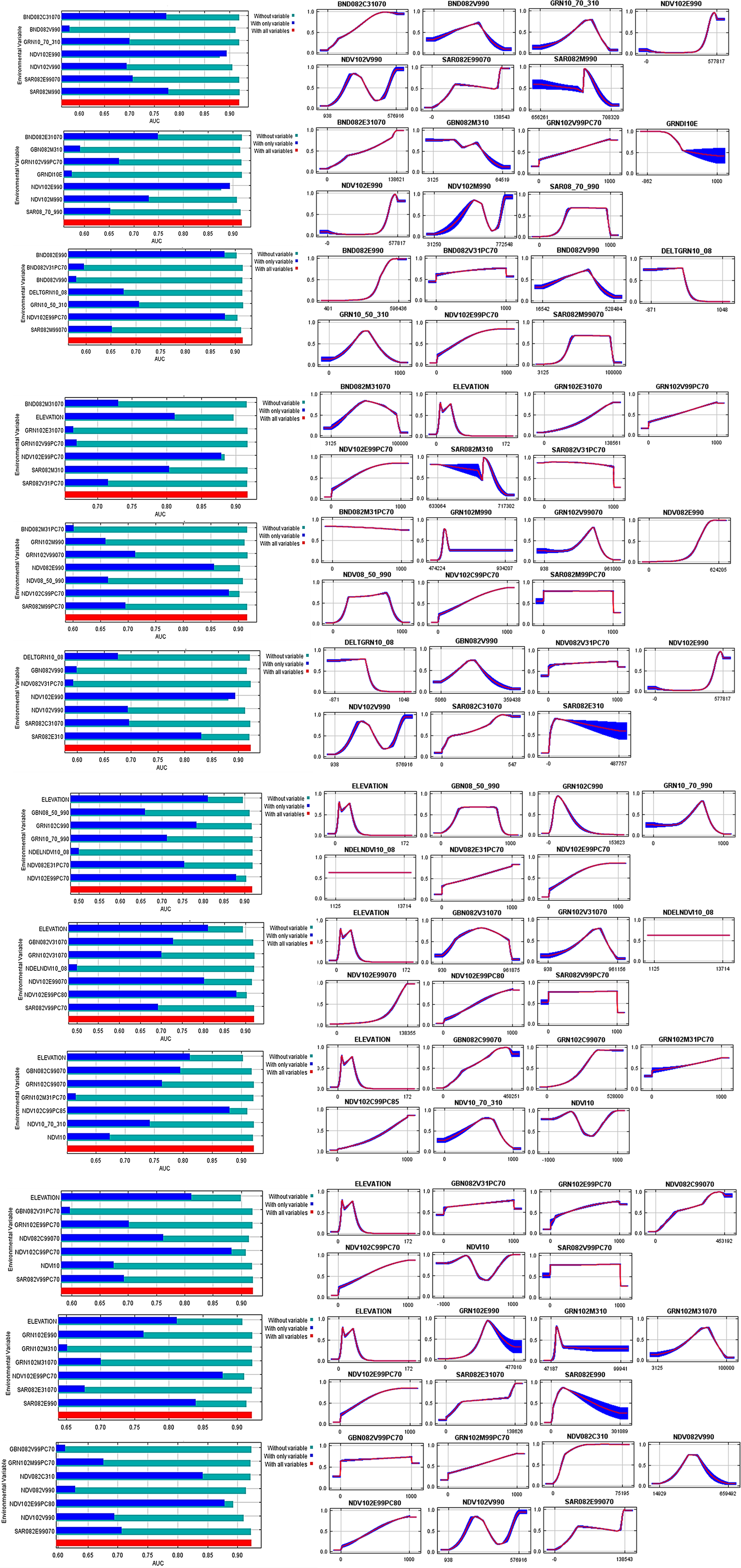

Supplement: S9 Fig — The jackknife of AUC for species graph (left) and response curve (right) results for each of the final models used to create the feature subset ensemble for the suitable general use habitat distribution. (TIF) [file pone.0294118.s010.tif]

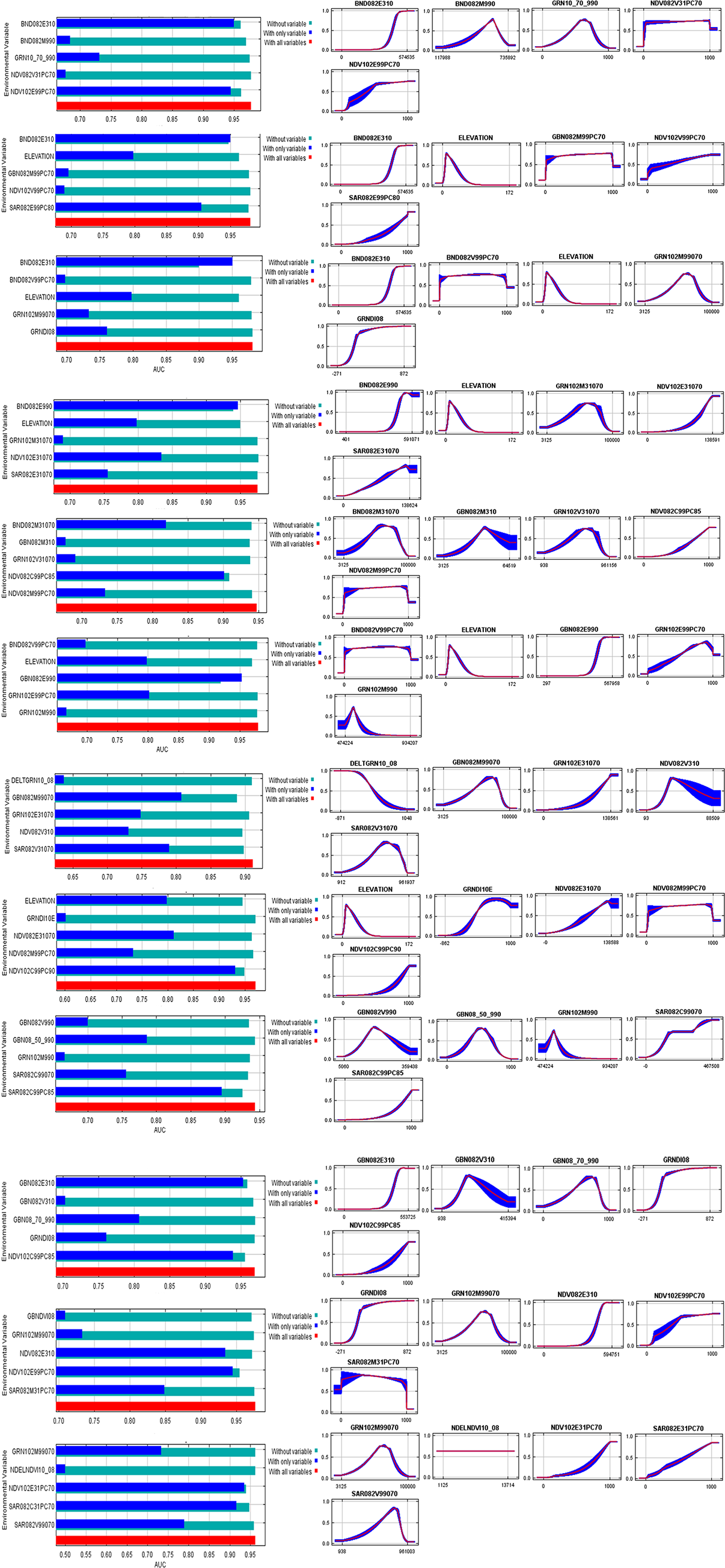

Supplement: S10 Fig — The jackknife of AUC for species graph (left) and response curve (right) results for each of the final models used to create the feature subset ensemble for the suitable nest site habitat distribution. (TIF) [file pone.0294118.s011.tif]

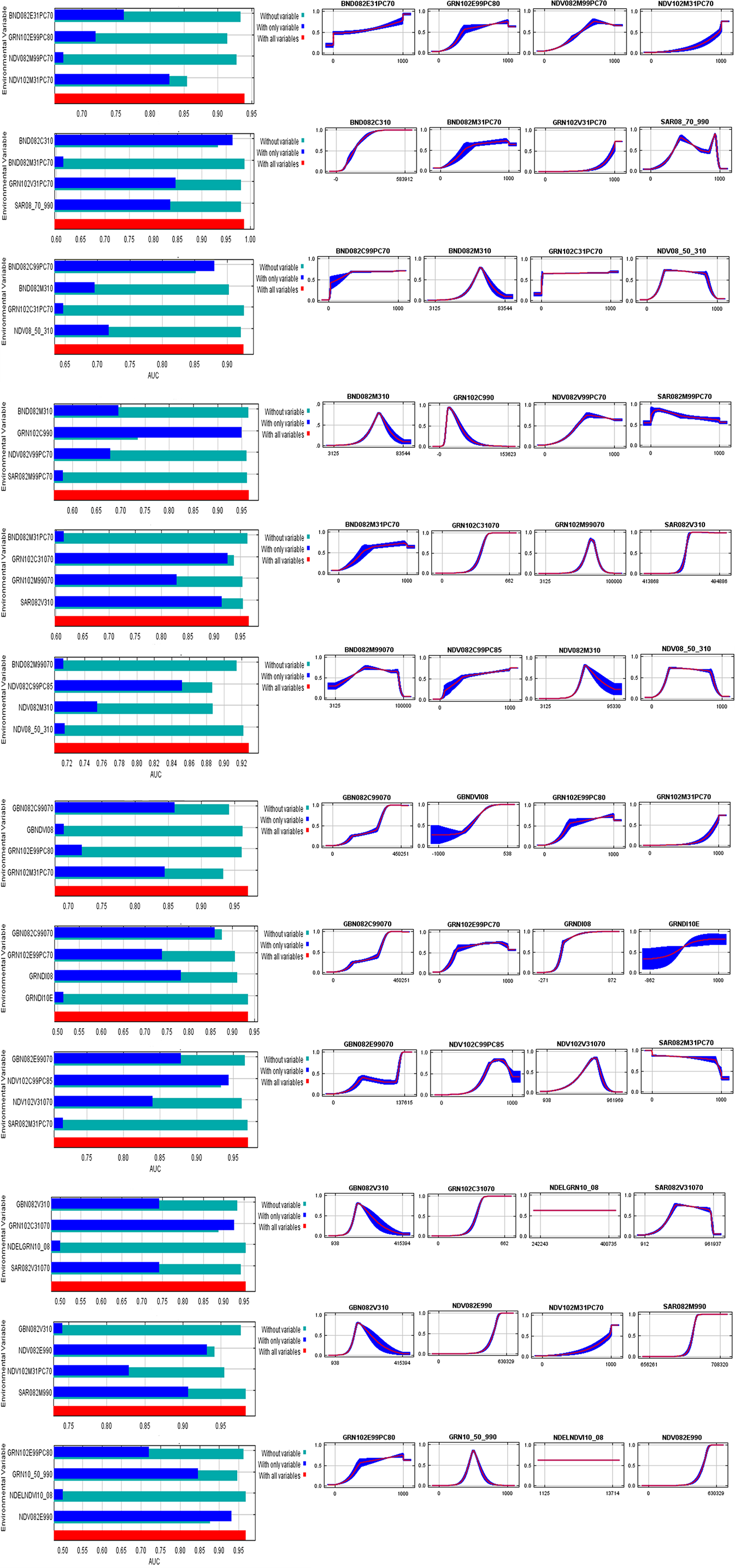

Supplement: S11 Fig — The jackknife of AUC for species (left) and response curve (right) results for each of the final models used to create the feature subset ensemble for the suitable roost site habitat distribution. (TIF) [file pone.0294118.s012.tif]
